# Facial recognition from DNA using face-to-DNA classifiers

**DOI:** 10.1038/s41467-019-10617-y

**Published:** 2019-06-11

**Authors:** Dzemila Sero, Arslan Zaidi, Jiarui Li, Julie D. White, Tomás B. González Zarzar, Mary L. Marazita, Seth M. Weinberg, Paul Suetens, Dirk Vandermeulen, Jennifer K. Wagner, Mark D. Shriver, Peter Claes

**Affiliations:** 1 Department of Electrical Engineering, ESAT/PSI, KU Leuven, Leuven, 3000 Belgium; 2 Medical Imaging Research Center, MIRC, UZ Leuven, Leuven, 3000 Belgium; 3 Centrum Wiskunde & Informatica, Science Park 123, 1098 XG Amsterdam, The Netherlands; 4 Department of Anthropology, Penn State University, University Park, PA 16802 USA; 5 Center for Craniofacial and Dental Genetics, Department of Oral Biology, University of Pittsburgh, Pittsburgh, PA 15219 USA; 6 Center for Translational Bioethics & Health Care Policy, Geisinger Health System, Danville, PA 17822 USA; 7 Murdoch Childrens Research Institute, Melbourne, 3052 VIC Australia; 8 Department of Biomedical Engineering, University of Oxford, Oxford, OX3 7DQ UK; 9 Department of Human Genetics, KU Leuven, Leuven, 3000 Belgium

**Keywords:** Quantitative trait loci, Ethics, Image processing, Genome-wide association studies

## Abstract

Facial recognition from DNA refers to the identification or verification of unidentified biological material against facial images with known identity. One approach to establish the identity of unidentified biological material is to predict the face from DNA, and subsequently to match against facial images. However, DNA phenotyping of the human face remains challenging. Here, another proof of concept to biometric authentication is established by using multiple face-to-DNA classifiers, each classifying given faces by a DNA-encoded aspect (sex, genomic background, individual genetic loci), or by a DNA-inferred aspect (BMI, age). Face-to-DNA classifiers on distinct DNA aspects are fused into one matching score for any given face against DNA. In a globally diverse, and subsequently in a homogeneous cohort, we demonstrate preliminary, but substantial true (83%, 80%) over false (17%, 20%) matching in verification mode. Consequences of future efforts include forensic applications, necessitating careful consideration of ethical and legal implications for privacy in genomic databases.

## Abstract (web summary)

Prediction of face from DNA followed by matching to facial images has been proposed for forensic applications. Here, Sero et al. present a different approach that can establish facial identity from DNA without directly predicting the face but is based on classifying given faces by individual DNA-encoded traits.

## Introduction

**Fig. 1:**
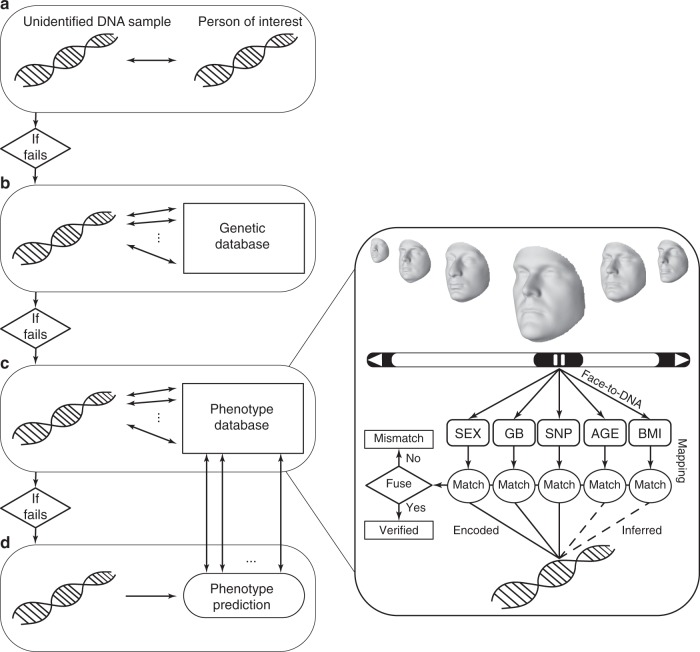
Flowchart representing the proposed paradigm in the context of existing DNA investigative tools. **a** Given an unidentified DNA sample, the first attempt is to match it to the DNA of a person of interest. **b** If the matching fails, then the same unidentified DNA sample is matched against DNA profiles of persons with known identity enrolled in the genetic database. **c** Our method could be of help if identification fails again. Each face-to-DNA classifier matches a face, in a gallery of faces (phenotype database), in terms of molecular features including sex, genomic background (GB), individual genetic loci (SNP), age, and body mass index (BMI) to a single probe DNA. Multiple, one per aspect, matching scores are fused together to provide an overall score, based upon which, it becomes possible to verify or reject a DNA profile against a face with known identity. **d** By using DNA phenotyping, predicted phenotypes could as well be matched against given phenotypes, but more likely lead to the last resort solution, namely showing it to the public and hoping that someone recognizes the individual. However, the current state of DNA phenotyping has not achieved this ability yet

DNA profiling, i.e. the identification of persons via DNA matching of an unidentified biological material (probe DNA) with biological material from persons of known identity, is considered the golden standard in forensic investigations^1–3^. Following Fig. 1, the identification fails if the DNA profile of the person of interest is unknown to the investigators, and if the database of candidate DNA profiles does not include the DNA sample that exactly matches the probe DNA. To help investigators out of an impasse, DNA phenotyping^1^, i.e. DNA-based prediction of phenotypes (hair color, eye color), and ancestry can be used to reduce the pool of candidates onto which to perform further investigations. Ultimately, the most desirable outcome from DNA phenotyping would be the prediction of facial shape from DNA for people to recognize. However, the human face is a complex and multipartite trait composed of distinct features (e.g., eyes, nose, chin, and mouth), whose development involves molecular and environmental interactions that are incompletely understood^4^. Furthermore, recent attempts to predict the face from DNA revealed that these renditions are driven by sex and ancestry only^5,6^. Therefore, the recovery of facial shape from DNA remains challenging.

In this work, we present a complementary avenue for support in current DNA investigations and propose to match a probe DNA profile against a database of known facial profiles. In contrast to DNA phenotyping, the idea is not to predict facial characteristics from DNA, but instead to predict DNA aspects from 3D facial shape using face-to-DNA classifiers; hence, all information is estimated from existing 3D facial images in a database. We apply this paradigm to two cohorts illustrating different recognition challenges. The first cohort is a heterogeneous mix of persons representing world-wide genetic diversity to recognize an individual’s genomic background in a genetically cosmopolitan sample. The second cohort is a homogenous European-derived sample that is used to investigate the contributions of individual genetic loci influencing facial variation, aiming at recognizing an individual within a population. Our preliminary results illustrate facial recognition power from DNA with strong contributions by genomic background, and more interestingly, by individual genetic loci discovered to be associated with facial variation in a genome-wide association scan (GWAS). We discuss how this work provides us with tools to establish human facial identity from DNA while avoiding some pitfalls of directly making DNA-to-face predictions. Furthermore, we underscore the need for further validation and proper safeguards, and bring to concern the privacy and data anonymity challenges for online genomic databases in personal genomics, personalized medicine, and genomic research.

## Results

### Data samples and partitioning

**Fig. 2:**
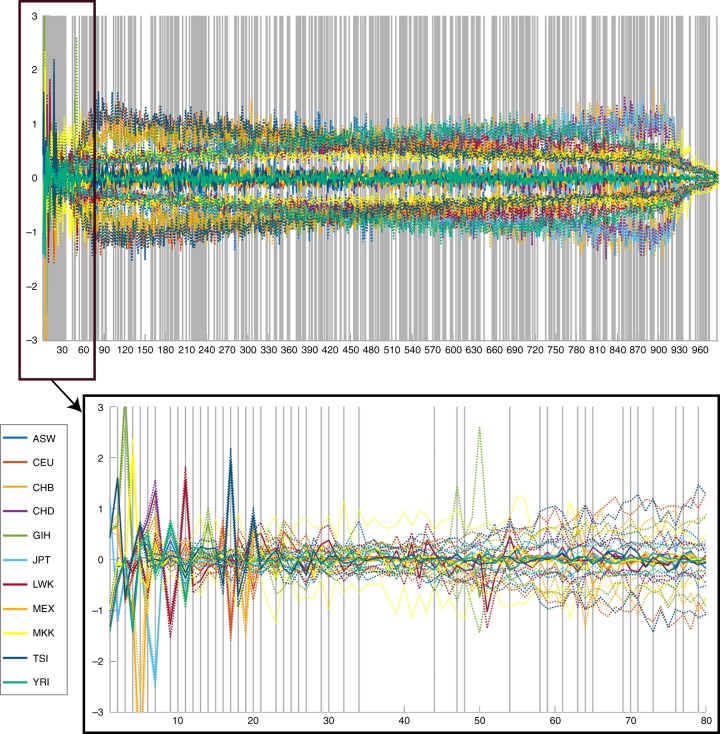
Global genomic diversity shown by a parallel coordinate plot. Each genomic principal component (PC) from the Hapmap dataset is a single parallel line, plotted against the median (solid lines) and upper/lower quartiles (dotted lines) coordinate values (vertical axis) for each of the 11 HapMap populations^10^ using different colors. Genomic PCs that tested significantly against facial variations are indicated with grey lines. The top genomic PCs, display strong between population differences, with population medians clearly either positive or negative. In contrast, in higher genomic PCs all population medians are close to zero, with population subgroups on either side (dotted lines). Interestingly, different populations structurally drive the between and within population differences along different genomic PCs. YRI: Yorubans from Ibadan; MKK: Masai from Kenya; LWK: Luhya from Kenya; CEU: Utah residents of Northern and Western European ancestry; TSI: Italians from Tuscany; CHB: Han Chinese from Beijing; JPT: Japanese from Tokyo; CHD: Han Chinese living in Denver; GIH: Gujarati Indians from Houston; MEX: Mexicans from the Southwest; ASW: African Americans from the Southwest

Our first study cohort (GLOBAL) consisted of *n* = 3,295 unrelated and genetically heterogeneous individuals recruited from a variety of sites worldwide (Supplementary Fig. 1). The main aim of this cohort was to identify the genomic background of a person in the context of genetic variation observed in diverse populations. Therefore, the genomic principal components (PCs) were the primary molecular features of interest (Fig. 2), which were modeled in the context of sex, age, and body mass index (BMI). Our second study cohort (EURO) was composed of *n* = 3,542 unrelated participants of European ancestry (Supplementary Fig. 2). The principal use of the EURO cohort was for testing recognition performance in a single relatively homogeneous population. Individual single nucleotide polymorphisms (SNPs) in genetic loci associated with facial variation were the main molecular features of interest and were modeled in the context of the first four genomic PCs of the EURO cohort, sex, age, and BMI.

Both cohorts, separately, were randomly partitioned into training, validation, and test sets. First, we created non-overlapping training and remaining sets. The training set, was used to identify associated molecular features and to subsequently train facial classifiers. The remaining set was further partitioned randomly into three non-overlapping folds: two folds combined constituted the validation set, one remaining fold constituted the test set. The validation set was used to learn how to fuse separate face-to-DNA classifiers into one matching score, and the test set was used to evaluate final recognition performances. This was done three times, such that each fold was used as test set once, while the other two folds as validation set. The partitioning used is a common strategy in machine learning to fit (train), select (validate) and evaluate (test) models and is diagrammed along with the methods work-flow in Supplementary Fig. 3.

### Facial phenotyping

**Fig. 3:**
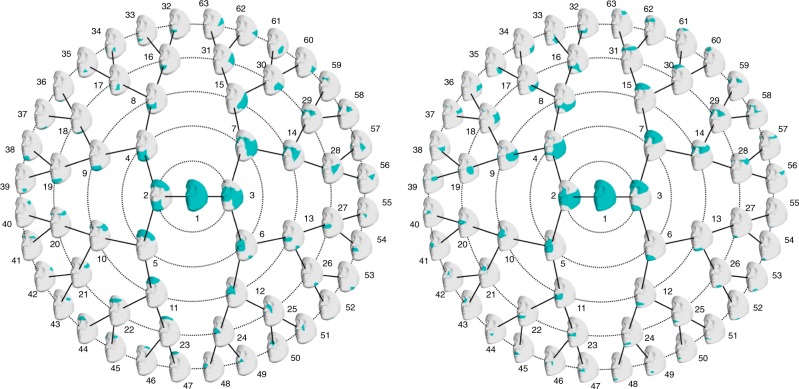
Hierarchical facial segmentations. Facial segments are colored in blue. (left) For the GLOBAL cohort, globally, the area including nose, eyes, cheeks, and upper lip was first separated from the rest of the face, and further partitioned into eyes and cheeks (quadrant 4, starting at segment 7), nose and lower-lateral facial area (quadrant 3, starting at segment 6). The remainder of the face was further partitioned into upper face (quadrant 2, starting from segment 5) and lower face (quadrant 1, starting at segment 4). (right) For the EURO cohort the nose, cheeks, lips were separated from the rest, and further decomposed into midface (quadrant 2, starting from segment 5), and cheeks and lower face (quadrant 1, starting from segment 4). The remainder of the face was composed of lower and upper face, which were further decomposed into upper face (quadrant 4, starting from segment 7), and lower face (quadrant 3, starting from segment 6)

Using a recently published facial phenotyping approach^7^, facial shape was divided into 63 global-to-local facial segments, for each cohort independently (Fig. 3). Between the two cohorts, we observed both similarities and differences in facial segments, which is not unexpected given that the different levels of population identity and diversity established different facial variations driving the segmentation.

By first applying a Generalized Procrustes Analysis (GPA) separately to the 3D points comprising each facial segment, followed by principal component analysis (PCA), facial shape features were obtained. As such, a shape-space for each facial segment is built independent from the other segments and their relative positions and orientations in lower-level (larger) segments. The number of PCs needed to adequately summarize shape variation for a given segment (Supplementary Fig. 4) was determined using parallel analysis^8^ (PA). As expected, we find that lower-level facial segments require more PCs, and that the PCs retained explain most of the total shape variance (GLOBAL cohort: median = 96%, min = 93%, max = 97%; EURO cohort: median = 94%, min = 89%, max = 97%). Finally, the multidimensional scores for the PCs constituted the facial shape features of each participant for each facial segment.

### Associated molecular features

Using the training set of each cohort only, we performed a series of association studies to test for significant relationships between the molecular features and the shape information contained in each of the 63 facial segments. All significantly associated molecular features and their facial effects are illustrated online^9^. As expected, we find strong statistical evidence in both cohorts for the effects of sex, age, and BMI on many facial segments and summarized in Supplementary Table 1. For all three aspects, the strongest statistical evidence was found in the full face, indicating largely integrated facial effects. Sex affected the masculinity/femininity of many facial segments, BMI affected large regions with underlying adipose tissues, and age affected facial structure due to degrading of skin elasticity.

In the GLOBAL cohort, we found that 382 of the 987 genomic PCs tested reached the false discovery rate (FDRd) corrected threshold (5.29 × 10^−4^). The statistical evidence of the 382 PCs are summarized in Supplementary Data 1. Supplementary Fig. 5 depicts the −log10 of the statistical evidence over all 63 facial segments combined for all 987 genomic PCs tested in a Manhattan plot-like fashion. The top 30 genetic PCs show strong statistical evidence. However, additional genomic PCs halfway and even in the tail end of the −log10 plot also showed strong statistical evidence. Furthermore, from Fig. 2, Supplementary Data 1 and the fact that we identified 382 genomic PCs indicates that we detected effects of genetic variation both between and within the 11 populations documented in the HapMap project^10^ (both well-documented recent admixtures and still cryptic patterns of substructure) that is contributing to facial shape. Therefore, we prefer to refer to this collection of associated PCs as genomic background instead of genomic ancestry because (1) genomic ancestry is typically obtained using a supervised approach and therefore biased and restricted to the subjective labels used to learn ancestry groups, and (2) we are most likely capturing within population stratification differences as well that are not ancestral. Furthermore, it avoids the need to specifically (and subjectively) label ancestral gene pools with the names of either historic or modern populations.

In the EURO cohort, only the first and the fourth genomic PCs showed statistically significant facial effects (*p* = 1.14 × 10^−41^ and *p* = 2.11 × 10^−8^, respectively). In a genome-wide association scan, we found a total of 2232 SNPs among 32 separate genetic loci that reached the FDRd threshold of 7.7 × 10^−8^ (Supplementary Figure 5). For each locus, we defined the peak SNP reported in Table 1, Supplementary Table 2 and illustrated in detail online^9^. Most genetic loci affected the nose, such as rs2980419 affecting complete nose structure; in contrast, rs4916071 affects the nose tip only. Many other genetic loci affected the chin area. Most parts of the face were affected by at least one locus, in only three occasions (rs7966105, rs13290470, rs200100774) the full facial segment was involved.

**Table 1:** Properties of 32 genetic loci identified in a GWAS on the EURO training data set

Locus	SNP	a	A	*p*-value	MAF	#SNPs	Class. Mod.
1p32.2	rs2404983	G	A	3.06E-08	0.076	6	D
1p32.1	rs4916071	A	G	2.06E-16	0.482	172	D, R
1p12	rs200100774	A	G	1.39E-10	0.165	126	D
1p12	rs61808932	C	T	5.92E-22	0.245	166	D, R
1q31.3	rs949977	G	C	2.36E-09	0.314	58	D
1q31.3	rs2821107	T	A	2.52E-22	0.209	287	D, R
2q31.1	rs970797	T	G	4.58E-11	0.432	2	D, R
2q36.1	rs1370926	G	C	1.61E-11	0.228	14	D
3q21.3	rs2955084	T	A	3.68E-10	0.078	2	D
3q21.3	rs2977562	G	A	1.80E-15	0.243	316	D
4q31.3	rs10020603	T	C	4.45E-18	0.195	9	D
4q31.3	rs17299889	A	G	4.67E-09	0.356	4	D
4q34.1	rs1059045	T	C	7.23E-13	0.466	24	D, R
6p21.1	rs227832	C	T	8.44E-14	0.257	214	D, R
6p21.1	rs9395084	C	T	5.58E-09	0.407	118	D
6p21.1	rs73735344	A	G	2.69E-12	0.153	112	D
6q23.2	rs402020	T	C	5.20E-12	0.308	22	D, R
7p21.1	rs1178103	G	T	8.24E-09	0.198	2	D
7q21.3	rs10238953	G	A	1.48E-31	0.138	229	D, R
7q21.3	rs2272224	C	T	1.17E-11	0.280	30	D, R
8p23.1	rs2980419	A	T	5.11E-08	0.478	1	D, R
9p22.2	rs13290470	G	A	2.53E-08	0.359	1	D, R
11p11.2	rs150863859	G	C	4.50E-08	0.113	2	D
11q22.3	rs7930466	G	A	5.80E-08	0.080	2	D
11q23.2	rs7925936	C	T	8.38E-09	0.169	2	D
12q21.31	rs7966105	A	G	8.18E-09	0.271	85	D
13q12.11	rs2985662	C	A	5.40E-08	0.360	1	D
14q12	rs143974562	T	C	3.10E-08	0.133	1	D
17q24.3	rs72866756	A	G	3.21E-14	0.366	163	D, R
17q24.3	rs11871949	C	T	4.63E-13	0.445	35	D, R
20p11.22	rs2424392	C	T	5.85E-09	0.234	24	D
20p11.22	rs6035946	A	T	1.53E-08	0.314	2	D

#SNPs number of SNPs reaching the FDRd *p*-value threshold (7.7 × 10E–8) within the same locus; Class. Mod. Classification model(s) deployed, can be dominant (D), recessive (R), or both (D, R) expressing the model of inheritance of each individual SNP used in the face-to-DNA classifiers

*SNP* single nucleotide polymorphism, peak SNP of the locus, *a* minor allele, *A* major allele, *MAF* minor allele frequency

### Face-to-DNA classifier, matching, and fusing

A face-to-DNA classifier labels given faces into possible categories of a molecular feature. Continuous variables were converted into two-class variables. Class balances in both cohorts are given in Supplementary Table 1 and Supplementary Data 1. For each significantly associated molecular feature, a classifier was learned using the training set of each cohort. For a given face outside the training set, the classifier generated probabilities of belonging to each of the two classes. Given the corresponding class label of a particular molecular feature from the probe DNA profile, the probability of the matching class was used as a matching score for that molecular feature. This expressed how well a given face matched a specific aspect of the probe DNA-profile. For example, if the probe DNA-profile shows male biological sex, a male face will generate a high matching score. In total, 382 and 50 classifiers were trained in the GLOBAL and EURO cohort, respectively.

For a face in a dataset, the outcome of all classifiers was a vector of matching scores, one for each molecular feature estimated. Subsequently, this vector of matching scores was combined, using a classification-based score fuser^11^, into one score, that reflects, how well the face matches overall to the probe DNA-profile. The more molecular features matching from the face to the probe DNA profile, the higher the overall score and, thus, more likely is it that the facial image matches the probe DNA profile. The fusing of scores for sex and age as a simple (*n* = 2) example is illustrated in Supplementary Fig. 6. From the validation set, vectors of matching scores (one for each molecular feature, thus n-dimensional vectors) of all individuals against each other, where each individual in turn is the probe, were obtained. Therefore, everyone’s facial image, when mapped against its own DNA-profile, generated a genuine matching vector. In contrast, imposter instances were generated when a facial image was matched against another person’s DNA-profile. The result is an n-dimensional space with genuine and imposter labeled instances from the validation dataset. Subsequently, the fuser learned from this labeled data how to discriminate genuine instances from imposter ones. When applied to a new matching score vector (e.g., from the test dataset), the fuser generated a posterior probability of being genuine, thus providing an overall matching score between a given facial image and a probe DNA profile.

### Facial identification and verification

**Fig. 4:**
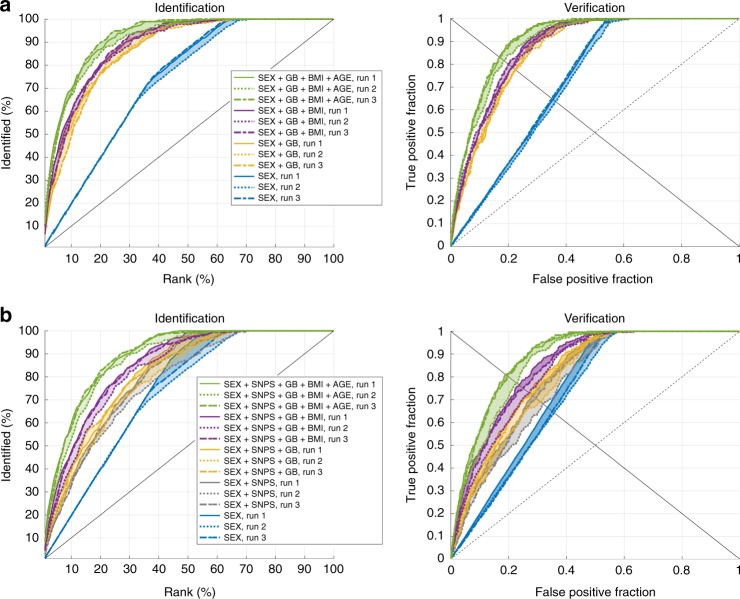
Biometric results. Identification and verification results **a** for the GLOBAL and **b** for the EURO cohort, respectively, by accumulating molecular features (Sex, genomic background (GB), body mass index (BMI), Age and single nucleotide polymorphisms (SNPS)). It is observed that the accumulation of molecular features clearly improves recognition power. Using the average of the three runs, for the GLOBAL cohort, in the identification setup, 36, 187, and 239 out of 275 test faces are identified within the top 1%, top 10%, and top 20% candidates, respectively. Likewise, in the EURO cohort; 22, 167, and 238 out of 296 test faces were identified perfectly within the top 1%, the top 10%, and top 20% candidates, respectively. In the verification setup, 228 out of 275 genuine matches (true positives) and 62,389 out of 75,167 imposters (true negatives) were correctly identified in the GLOBAL cohort; while, 237 out of 296 genuine matches and 69,542 out of 86,927 imposters were correctly recognized in the EURO cohort. Different runs on three non-overlapping test datasets are plotted as solid, dotted and dash-dotted lines, respectively

We tested our ability to classify faces in the context of multiple molecular features using a biometric identification and verification setup on the test dataset of both cohorts. Furthermore, we compared our approach of face-to-DNA classifiers against a DNA-phenotyping approach^6^ based on DNA-to-face regressions followed by face-to-face matching (Supplementary Note 1, Supplementary Figs. 8–12, Supplementary Tables 3–6). In the biometric identification setup (Fig. 4, Supplementary Fig. 7), the performance was evaluated using cumulative match characteristic (CMC) curves for combined and individual molecular features, respectively. High identification rates and rapid relative increases in the CMC indicate better performance. In the biometric verification setup, the performance was evaluated using receiver operating characteristic curve (ROC) analyses (Fig. 4, Supplementary Fig. 7) for combined and individual molecular features, respectively. Numeric results are given in Tables 2 and 3 for the GLOBAL and EURO cohort, respectively.

**Table 2:** Average identification and verification results for the GLOBAL cohort

GLOBAL Cohort	EER	*σ*	AUC	*σ*	R1	*σ*	R10	*σ*	R20	*σ*
SEX	35.7	0.7	72.3	0.9	1.46	0.00	19.66	0.04	39.81	0.34
GB	31.1	0.9	77.3	0.4	4.61	2.63	36.41	1.96	56.55	1.61
BMI	37.1	0.8	67.6	0.9	1.34	0.21	18.08	0.52	35.80	0.86
AGE	39.4	0.7	66.8	2.1	1.46	0.00	19.66	0.04	38.23	1.02
SEX + GB	22	0.7	86.2	0.2	7.53	1.18	52.67	3.93	76.58	0.45
SEX + GB + BMI	20.8	0.8	87.5	0.4	8.86	2.01	58.62	1.20	79.98	0.98
SEX + GB + BMI + AGE	17.5	0.9	90.2	0.8	12.01	1.25	67.84	1.48	87.38	2.20
X = [SEX, AGE, BMI, GB]	19.8	1.5	88.2	0.9	11.65	1.80	65.66	2.09	84.10	1.11

The results for a DNA-to-Face prediction strategy from a set of predictors X are given in the last row. All values are given in percentage. Random performance is given as EER = 0.5, AUC = 0.5, R1 = 1%, R10 = 10%, R20 = 20%. % refers to the percentage of individuals in the gallery (*n* = 275, test dataset)

*EER* verification equal error rate, *AUC* verification area under the curve, *R1* rank 1% identification rate, *R10* rank 10% identification rate, *R20* rank 20% identification rate, *σ* standard deviation, *GB* genomic background, *BMI* body mass index

**Table 3:** Average identification and verification results for the EURO cohort

EURO Cohort	EER	*σ*	AUC	*σ*	R1	*σ*	R10	*σ*	R20	*σ*
SEX	34.7	1.2	73.5	1.3	1.35	0.00	19.64	0.04	39.96	0.08
SNPS	40.2	2.1	63.6	1	2.48	0.98	21.90	1.08	38.49	0.59
GB	42.7	1.8	60.7	0.5	1.35	0.00	17.05	2.53	30.93	1.42
BMI	38.5	1.9	66.1	1.1	1.24	0.19	18.17	0.41	34.88	1.24
AGE	38.9	1.8	67.1	1	1.35	0.00	19.53	0.18	37.92	0.74
SEX + SNPS	29.9	1.8	79	1.6	3.72	1.21	34.09	1.87	56.21	2.55
SEX + SNPS + GB	28.6	1.9	80.8	1.6	5.19	0.70	38.60	3.02	58.92	3.21
SEX + SNPS + GB + BMI	25.2	1.8	84	1.5	5.30	0.51	45.26	3.51	69.87	3.78
SEX + SNPS + GB + BMI + AGE	20.6	2.1	88	1.4	8.92	0.97	56.66	3.45	81.27	1.67
SEX + GB + BMI + AGE	22.1	1.7	86.3	1.3	5.31	0.01	50.23	3.72	75.51	1.55
X = [SEX, AGE, BMI, GB]	25.6	0.8	81.8	0.6	5.64	1.41	44.36	2.34	68.62	2.54
X = [SEX, AGE, BMI, GB, SNPS]	25.4	0.8	82.5	0.5	6.32	0.52	48.20	0.80	70.88	0.29

The results for a DNA-to-Face prediction strategy from a set of predictors X are given in the last two rows. All values are given in percentage. For the EURO cohort, the performances of the DNA-to-Face prediction and Face-to-DNA classification setups are shown with and without the contribution of the SNPs from Table 1. Random performance is given as EER = 0.5, AUC = 0.5, R1 = 1%, R10 = 10%, R20 = 20%. % refers to the percentage of individuals in the gallery (*n* = 295, test dataset)

*EER* verification equal error rate, *AUC* verification area under the curve, *R1* rank 1% identification rate, *R10* rank 10% identification rate, *R20* rank 20% identification rate, *σ* standard deviation, *GB* genomic background, *BMI* body mas index

For both cohorts, the observations were consistent across the three test folds. The results for sex, age, and BMI were similar, and reflected a good sensitivity of faces as a function of these aspects. However, each of these features separately lack the specificity needed to identify individuals. Strong increases in specificity were achieved only in the combination of different estimated molecular features. The performances of all estimated molecular features from 3D facial shape combined were substantial in both cohorts, but were slightly better in the GLOBAL cohort. For the GLOBAL cohort, the performance of the 382 genomic PCs is quite impressive and illustrates that diversity in genomic background is a primary driver of facial variation, and thus well estimated from 3D facial shape. The cumulative contributions of the genomic PCs generated a strong increase in performance when added to sex. The further addition of BMI and age, separately, showed lower yet further increase in performance. For the EURO cohort, the performance of the two associated genomic PCs fused was the least informative, but still better than chance alone. We expected some genomic background diversity in this cohort, but not to the same extent as the GLOBAL cohort. More interestingly, the performance of the 32 peak SNPs is notable and clearly shows an increase in performance (equal error rate (EER) down by 5 %) when included with sex in the model. We tested additional genomic PCs (beyond the first four) in the EURO cohort, and observed a reduced performance, but with consistent improvements when adding the individual SNPs (Supplementary Note 2, Supplementary Table 7).

In comparison to DNA-to-face regressions, our results show an improvement in identification and verification performances. More importantly, in contrast to our results, individual genetic loci did not contribute following the DNA phenotyping strategy. This is also conform the observations made in recent attempts to predict the face from DNA^5,6^.

## Discussion

The identification of a person based on an unidentified DNA sample can be performed via DNA profiling: the unidentified DNA sample is matched against the DNA of a person of interest and if a match is observed then a probability of identity can be established, whereas a non-match indicates non-identity^12^. However, the identification fails if the DNA profile of the person of interest is not available. As an alternative, predicting phenotype from genotype data and then match this predicted phenotype to other phenotypes (DNA phenotyping^1^), can also be used to perform recognition^13^. However, DNA phenotyping for complex traits is difficult due to the effects of multiple loci, unmeasured or unknown non-genetic effects, and genetic and epigenetic interactions, many of which are largely unknown. Additionally, the phenotypic complexity of facial morphology has typically been oversimplified during genetic mapping efforts^7^. Therefore, any attempt to recover the complete facial structure from DNA remains challenging. Here, we perform facial recognition from DNA by directly matching given faces to a probe DNA-profile. This approach represents an additional and complementary venue that can be used as further support in DNA-based investigations. Computationally, in contrast to the challenging task of genome-based phenotype prediction, our paradigm is embedded in facial image classification, which is an active area of research in machine learning (i.e. 3D face based prediction of sex^14^, age^15^, ancestry^16^, and sexual orientation^17^).

Established biometric authentication systems are built upon primary identifiers, i.e. biological features (voice patterns, hand/finger/face/ear geometry, iris, retina, fingerprints, gait, handwriting, DNA) that precisely determine the identity of an individual. Any feature that gives insufficient information to individuate people, yet improves the identification rates if employed along with primary identifiers, is referred to as a soft biometric feature^18^ (sex, ancestry, age, height, weight, and eye/skin/hair color). In this context, our study proposes a paradigm that matches between two primary but different identifiers, DNA and facial shape, through a set of soft biometrics. SNP genotypes are essentially added to the list of soft biometrics and genomic background, is used as an elaborated proxy for ancestry. Note that, additional DNA-inferable features (skin pigmentation, hair and eye color^1^) can also be incorporated when using facial image texture in addition to shape. Our results clearly indicate that the accumulation of multiple simple molecularly derived features increases recognition specificity. This is an aspect of biometrics that traces back to the inception of the field and was already understood in the early system of Alphonse Bertillon^18^.

The ability to identify a DNA profile against a dataset of phenotypes with known identities was first explored by Lippert et al.^6^. In their work multiple phenotypes (facial shape/color, sex, age, height, weight, BMI, skin/eye color, ancestry, voice) were estimated from DNA profiles and subsequently matched against corresponding phenotypes with known identities. In both our work and that of Lippert et al., the aim is to create a common embedding for both genotype and phenotype information, and therefore bridging the gap. Once created, distances in the embeddings are defined to perform identification. In contrast to our work, the embedding in the work of Lippert et al. is mainly at the level of multiple phenotypes predicted from genotypes and not at the level of multiple genotypes predicted from a single facial phenotype. However, of particular interest was the comparison against DNA-based facial phenotyping followed by face-to-face matching. Our results indicate a better performance following face-to-DNA classifiers only with the added contribution of individual genetic loci in the EURO cohort.

When putting our results in perspective, the performances are lower than the state-of-art methods for matching the same kind of primary identifier (face-to-face, fingerprint-to-fingerprint, ear-to-ear). When comparing faces directly, biometric performances reported are typically higher than those reported here^19^. However, in more challenging scenarios, such as facial recognition across different facial expressions, base-line facial recognition systems perform worse^20^. The verification results from both cohorts are comparable to human recognition performance on challenging scenarios in video (e.g., recognizing individuals in sequences where faces are not displayed in standardized frontal images) and dissimilar image qualities in still-face pairs (e.g., two images displaying the same person with different clothing/hairstyle under different ambient constraints) described in the work of Phillips et al.^21^. Furthermore, when compared to the performance of a group of examiners with rigorous training in forensic face recognition^22^ under real-case scenarios, our system achieves comparable area under the curve values. Finally, a recent tool for iris recognition from a mobile device^23^ also reported lower recognition performances than those reported here. In conclusion, there is room for improvement, but our results are already tangible in the context of other biometric authentication scenarios.

We report slightly better performances for the GLOBAL cohort in comparison to the EURO cohort. In both cohorts, it is expected that individuals who are less typical to others in the cohorts will be identified more easily, a concept inherent to recognition tasks^24^. However, directly comparing the results is futile since they represent different recognition challenges. A higher genomic diversity in the gallery, as in the GLOBAL cohort, against which identity is sought, leads to an easier recognition task, but does not necessarily imply a better ability to recognize an individual. In other words, genomic diversity implicitly increases the number of subgroups in a group of people living together and leads to an increased recognition power, but at the level of subgroups, not at the level of the individual. The recognition is more challenging if, for example, a European person compares herself/himself to a group of European faces rather than to a diverse group of people. In fact, by design, recognition in the GLOBAL cohort is limited to an individual’s population background. Therefore, only the identity of a group of individuals as sharing a similar background is obtained. In contrast, the EURO cohort is more locally scaled in genomic ancestry, and tested our ability to identify an individual within a single population. Therefore, the focus was on investigating the degree to which genetic loci affecting facial shape within Europeans can individuate faces. Of strong interest is that we performed a GWAS and used 32 specific genetic loci identified. However, the results are still preliminary and far from perfect. Although many individuals are identified within a top 10% of a sorted gallery, performances on the single best match (rank 1), remain limited. Also, the EERs under verification indicate clear room for improvement. Future efforts include collecting larger cohorts in a GWAS as well as finding rare variants using family-based linkage analysis and other methods based on tracking co-segregation.

It is unequivocally both irresponsible and scientifically unjustified to make any attempt to recover ungiven facial shape from DNA in a real-life forensic scenario unless the system has undergone rigorous scientific validation and peer review. Based on previous work^5^ and the results reported here, facial predictions from DNA remain purely sex, ancestry-driven and individual genetic loci failed to improve the results convincingly. Similar results were reported by Lippert et al.^6^, and similarly do not provide an accurate facial prediction at the level of an individual^25^. Another complication of DNA-based facial phenotyping is that its forensic success depends on the ability of the image to elicit recollections from persons who know the subject or reports of encounters from persons who meet the subject. Furthermore, if a single predicted facial image is produced, observers may give too much credit to the single facial image presented, which might dangerously focus an investigation on either a person who looks too average to be true or, worse yet, creates a specific face who looks just like some subset of people or perhaps even like a single person in the sample that was used to build the prediction model. The latter is an algorithmic bias that can occur when some populations are underrepresented during model building.

The proposed methodology works on databases of facial images only, not augmented with other meta data (sex, or even genetic data). Therefore, random image databases, as pulled from the internet e.g., are becoming of use. The molecular features used here are deducted from the facial shapes only, in contrast to the work of Lippert et al.^6^. Moreover, recent devices (the Microsoft^TM^ Kinect, Microsoft^TM^ Surface, the latest iPhone^TM^), are providing the means to capture 3D facial data. Interest of the lay-public in 3D imaging/printing is pushing these new devices to be used in user authentication and distributed via social networks. Furthermore, a substantial body of research in computer vision is focused on 3D facial reconstruction from 2D images^26^. Therefore, many reference images are available in governmental databases (e.g., driver’s licenses, passports) that can be tested against a probe DNA. The result would be a sorted database of facial images by the similarity of their predicted molecular features. Therefore, hundreds of (somewhat equally matching) actual facial images can be presented. Doing so should more clearly expose variability (thus system error) in the matches achieved, and, thus inform the user regarding the performance of the algorithm on a case by case basis.

Considering our results in Fig. 4, the best match always reaches low recognition rates and better performances are tangible only at rank 20. With these preliminary outcomes, we observe that the best match is hardly achievable. Therefore, we strongly advise against using the single “best” match as an exact image of the person of interest, as this will be most likely inaccurate. Such an interpretation would only be meaningful in an exceptionally accurate system and quite possibly only if the person of interest is in the facial database being queried, which is unknown. In contrast, presenting multiple images will communicate variability in accuracy which might help avoid prematurely targeting a single individual and “keeping an open mind” regarding the specific details of the facial appearance of the person of interest. To avoid potential algorithmic biases, one must properly represent all minority groups in the databases being queried. Such biases are also known for face-to-face recognition algorithms and require appropriate investigations^27,28^. Another point that may raise concern here, refers to the human capacity to discriminate individuals based solely on minute differences among a vast group of faces. When untrained individuals with no professional experience with face recognition and machines, are asked to respond how likely two faces from a pair of images/videos were of the same person^21^, it was observed that a computer can handle millions of face pairs, while an individual can rate maximum 250 face pairs. Compared to the study of Phillips et al.^21^, our problem is amplified in that identity has to be established from a list of hundreds of somewhat similar faces that match to a probe DNA profile. However, because the minute differences among these faces represent the variability of the system, it is important to describe their commonalities. Arguably, however, absent research, likely by psychologists, it remains to be seen how the use of such a system outside the current laboratory setting can be potentially transferred into the real world.

Future investigations involve: First, in the GLOBAL cohort, we are limited by the ancestry variation captured by our reference data (HapMap 3 Project^10^). Therefore, it is of interest to explore other datasets as a reference to establish genomic background at worldwide (e.g. the 1000 Genome Project^29^) along with more locally scaled variations (e.g. the POPRES^30^ dataset and the Genographic Project^31^ for a within Europe scaled ancestry reference). Furthermore, the identification of an individual within a single population requires further investigation on other population samples. Second, the loci and SNPs extracted from our European based GWAS, were extracted by association only. It is of interest to expand on these results with functional analysis to identify causal variants in the loci detected, as a replacement for the SNPs currently used and defined as peak SNPs. Furthermore, future investigations are in order to test the effects of the genetic loci identified in non-European populations. Third, in light of growing concerns about researchers inadvertently integrating racial bias into machine learning^32^, we control for racial bias by 1) looking at a specific homogenous population only, and 2) by using ancestry defined by the HapMap 3 Project, in which different population samples were balanced. Fourth, for both the cohorts studied, we used self-reported information on age and body size (height and weight, which were used to calculate BMI) as proxies for DNA-inferred values. In Supplementary Note 3 and Supplementary Fig. 13, we report observations on simulated DNA-predicted age and BMI; however, we highlight that these DNA-based predictions are still imperfect. Fifth, additional discussions include the challenges of our proof of concept in the context of monitoring and adjusting Artificial Intelligence (AI) systems, especially when these are implemented in resource-poor settings of local and state governments. Despite the growing interest and developments in AI by experts on high-quality datasets, the use and understanding of AI in practice remains daunting and challenging. Finally, an important challenge in forensics involves the ability to use our paradigm based on often limited and contaminated DNA material.

Some forensic and criminal justice challenges raised elsewhere with regard to DNA phenotyping^33^ apply similarly to matching given faces to DNA. Our results have no value to prosecutors as inculpatory evidence (i.e., all of its value in forensics/intelligence is during the investigatory stage). Furthermore, these methods also raise undeniable risks of further racial disparities in criminal justice that warrant caution against premature application of the techniques until proper safeguards are in place (we expand on these challenges in Supplementary Note 4). Additionally, several interests (journal publishers, federal funding agencies), are promoting the broader sharing of genomic data among researchers across institutes and for uses other than for which they were initially collected. To this end, human participants’ data sharing protocols de-identify the participant data. However, conditioned on future efforts, our work provides a means by which de-identified genomic data can be reconnected to the participants, when they are in facial image datasets that can be accessed, using the information about genotype contained in the human face. Therefore, the work reported here further underscores the importance of continued deliberation and additional ethical, legal, and social implications (ELSI) research in this area, as elaborated on in Supplementary Note 5.

In conclusion, we propose a facial recognition system from DNA that avoids the need to predict an ungiven face from DNA. Unsupervised genomic PCs showed substantial recognition power on the level of population background. More interestingly, is a significant contribution from individual genetic loci identified in a facial GWAS. However, our results are preliminary and on well-defined data cohorts. Future improvements are required, before individuals can be identified uniquely. Furthermore, this work underscores the need for (A) rigorous scientific validation and critique; (B) public input on the societal merits of the tool and indicate strong trust and support for its use; (C) assessments by relevant technical and ELSI experts regarding the individual and collective implications, and (D) implementation of adequate legal and regulatory safeguards.

## Methods

### Data cohorts

For the GLOBAL cohort, we selected *n* = 3,366 individuals with genotype data and 3D facial images for analysis. These were collected with informed consent as part of several studies based at The Pennsylvania State University and sampled in the following locations: State College, PA (IRB #44929 & #4320); New York, NY (#45727); Urbana-Champaign, IL (#13103); Cape Verde; Dublin, Ireland; Rome, Italy; Warsaw, Poland, and Porto, Portugal (#32341); and Twinsburg, OH (#2503). The individuals were genotyped on the 23andMe v3 and v4 arrays (23andMe, Mountainview, CA) and Illumina HumanHp200v1 BeadChip (Illumina Inc., San Diego, CA) platforms. We selected a core set of 118,420 LD-pruned autosomal SNPs (window size = 50 kbp, SNPs = 5, VIF = 2), which represent the intersection across all platforms, to characterize the genetic structure of the sample using principal components analysis (PCA). A genomic PCA space was first defined by a set of 11 reference populations from the publicly available HapMap 3 dataset (YRI: Yorubans from Ibadan; MKK: Masai from Kenya; LWK: Luhya from Kenya; CEU: Utah residents of Northern and Western European ancestry; TSI: Italians from Tuscany; CHB: Han Chinese from Beijing; JPT: Japanese from Tokyo; CHD: Han Chinese living in Denver; GIH: Gujarati Indians from Houston; MEX: Mexicans from the Southwest; ASW: African Americans from the Southwest). The multivariate genetic background space was constructed by carrying out PCA on the genotypes of *n* = 988 unrelated HapMap individuals for the same *n* = 118,420 SNPs mentioned above. Into this space, we projected the *n* = 3,366 individuals from our dataset, as illustrated in Supplementary Fig. 1. This ensures that the shape of the PCA space is only defined by individuals from the HapMap dataset, which can be reproduced independently. This dataset further included self-reported information on age and body characteristics (height and weight). Sex was determined using X-chromosome homozygosity/heterozygosity. The youngest participant is a 17-year-old boy and the oldest a man of 88-years-old; the average age is 28 years. The mean body mass index is 24.65 kg m^−2^. A total of *n* = 3,295 individuals were retained after reducing the cohort for missing self-reported information or because of 3D image mapping artifacts. The majority are females (71%).

A single EURO cohort was obtained by merging two datasets of participants sharing common European ancestry: The Pittsburgh (PITT) sample and the Penn State (PSU) sample^7^. The PITT sample comprised of 2,449 unrelated participants of European ancestry and median age of 23 years. The PSU sample comprised of 2,059 unrelated participants of European ancestry and median age of 22 years. In this work, specifically, we retained a total of *n* = 1,793 and *n* = 1,749 participants of the PITT and PSU sample, respectively, after removing individuals younger than 18 years of age, or missing information on sex, age, height, weight, or with 3D image mapping artifacts. Age and body characteristics (height and weight) were self-reported. Sex was determined using X-chromosome homozygosity/heterozygosity. The majority are females (64%), and the oldest participant is an 82-year-old woman; the average age is 28 years. The mean body mass index is 24.90 kg m^−2^. A total of *n* = 3,542 individuals were available after merging both samples.

For the PITT sample, participants, were genotyped on the Illumina (San Diego, CA) OminExpress + Exome v1.2 array plus 4,322 investigator-chosen SNPs included to capture variation in specific regions of interest based on previous studies of the genetics of facial variation. For the PSU sample, participants sampled from 2006–2012 (IRB #32341) were genotyped on the Illumina Human Hp200c1 BeadChip (Illumina Inc., San Diego, CA). Participants sampled from 2013–2016 (IRB #44929, #13103, #2503, and #4320) were genotyped on the 23andMe v3 and v4 arrays (23andMe, Mountainview, CA). Standard data cleaning and quality assurance procedures were performed^34^. All samples were evaluated for concordance of genetic and reported sex, evidence of chromosomal aberrations, biological relatedness across study participants, ancestry, genotype call rate, and batch effects. SNPs were evaluated for call rate, discordant genotype calls between duplicate samples, Mendelian errors in HapMap control parent-offspring trios, deviation from Hardy-Weinberg genotype proportions, and sex differences in allele frequency and heterozygosity.

Genotype data in the PITT and PSU samples, separately, were imputed using the 1000 Genomes Project Phase 3 reference panel^29^. SHAPEIT2 was used for pre-phasing haplotypes and IMPUTE2 was used to impute nearly 35 M variants. SNP-level (INFO score > 0.5) and genotype per participant-level (genotype probability > 0.9) filters were used to omit poorly-imputed variants. A further reduction of SNPs with MAF <5% was performed, before aligning allele encodings in both datasets with the 1000 Genomes Project and to merge them into a single cohort of European ancestry.

Population structure was assessed using PCA of approximately 100 K autosomal SNPs chosen for call rate (>95%), MAF (>5%), and LD (pairwise r2 < 0.1 across variants in a sliding window of 10 Mb). Tests of genetic association between the first 20 PCs and all SNPs confirmed that PCs did not represent local variation at specific genetic loci. Based on Supplementary Fig. 2, four PCs were sufficient to capture population structure within this European-derived cohort towards the purpose of a GWAS.

The data in both cohorts was partitioned into training, validation and test sets and used throughout the methods as schematically represented in Supplementary Fig. 3.

### Facial phenotyping

Three-dimensional images composed of surface and texture maps were taken using the 3dMD Face (3dMD, Atlanta, GA) or Vectra H1 (Canfield Scientific, Parsippany, NJ) 3D photography systems. Participants were asked to hold their faces with a neutral expression and close their mouth for the picture. Intensively sampled morphometric descriptions of facial shape are obtained from 3D facial images as homologous spatially-dense (*n* = 7,150) quasi-landmark (QL) configurations^7^. Note that, by homologous, we mean that each quasi-landmark occupies the same position on the face relative to all other quasi-landmarks for all individuals. Hence, this approach provides detail on the more overt as well as the subtler facial aspects and is independent of a potential ‘facial perception’ bias (e.g., concentration of landmarks on perceptually salient features of the face). A Generalized Procrustes Analysis (GPA) was applied to correct for changes in position, orientation and scale of both the original and reflected configuration combined. After Procrustes superimposition, a single shape can be decomposed into its asymmetric and bilaterally symmetric component. The average of an original and its reflected configuration form the symmetric component while the difference between the two configurations constitutes the asymmetric component. Although of interest, in this work we currently ignore variations in facial asymmetry and use the symmetric component only.

The division into segments of a biological organism according to its function, embryological origin and anatomy, is a well-known concept in evolutionary and developmental biology under the heading of modularity and integration^35^. We applied the same principle to the facial complex, which is composed of multiple subunits (for example, eyes, mouth, nose, chin, cheeks) integrated to function as a whole. In our study, 3D spatial covariation between each pair of QLs across each dataset was used to guide a facial division into regions; therefore, a morphologically-driven segmentation was implemented^7^. Starting from the superimposed and symmetrized QL configurations, the RV coefficient between each pair of QLs was computed to construct a squared similarity matrix as input to a hierarchical spectral clustering. The output were facial segments, where each segment had a cluster label and the QLs within a segment were assigned the same cluster label. This was done in a hierarchical manner, such that first the full face was split in two segments, and subsequently each of these was again split in two segments. This process was repeated five times generating a total of 63 facial segments hierarchically linked to one another. We refer to the number of repeats as levels. Each level has 2^level^ modules. For example, level 0 corresponds to one cluster which is the complete face. Level 1 has 2 clusters, level 2 has 4 clusters, and so on.

In order to capture shape features of the facial complex, PCA was performed constructing a shape-space for each facial segment. At each level, for each segment, all QLs having the same cluster label are subjected to a new GPA. Therefore, a shape-space for each facial segment is constructed independently of the other segments and its relative positioning within the full face. After GPA, each facial segment was spanned by an orthogonal basis using PCA combined with parallel analysis^8^ to determine the number of significant PCs. Therefore, each segment generated multi-dimensional (one for each PC retained) shape features for all participants. Open-source software for the complete facial phenotyping pipeline is available, see Code Availability.

### Statistical study detecting molecular factors of 3D shape

We implemented a series of association studies to investigate the effects of molecular features and to select them accordingly. Since each facial segment was represented by multiple dimensions of variation (PCs), the association studies were conducted with a multivariate Canonical Correlation Analysis (CCA). In brief, CCA extracts the linear combination of PCs from a facial segment that has maximal correlation with the molecular feature under investigation. Significance testing was based on Rao’s F-test approximation (right-tail, one-sided). We used the function canoncorr from Matlab^TM^ 2016b. Sex, age, BMI and 987 genomic PCs in the GLOBAL cohort and sex, age, BMI, four genomic PCs and *n* = 5,383,799 individual SNPs in the EURO cohort were investigated against each of the 63 facial segments. Genomic PCs were investigated as binary variables (cfr. variable conversion). SNPs were investigated following a GWAS paradigm on the EURO cohort, under the additive genetic model (AA = 0, Aa = 1, aa = 2), after correcting for confounding variables including sex, age, BMI, four (continuously coded) genomic PCs, and the dataset (PITT or PSU) identifier.

Given the burden of multiple comparisons in both cohorts, separately, we computed a false discovery rate (at a level of 0.05) adjusted significance threshold (FDRd) of Benjamini & Yekutieli^36^, that is accurate for any test dependency structure. A molecular feature was selected for subsequent analysis and classification if at least one out of the 63 facial segments reached the FDRd threshold.

In the EURO cohort, we observed 2,232 FDRd significant SNPs across 32 loci using a 500 kb window and linkage disequilibrium (LD) > 0.5. For each locus, a peak SNP was defined as the SNP generating the highest association (lowest p-value) in any of the 63 facial segments. Genes 500 kb up- and downstream of the peak SNPs were identified using the Table Browser of the UCSC Genome Browser (http://genome.ucsc.edu/cgi-bin/hgTables).

### Face-to-DNA classifier, matching and fusing

We transformed continuous variables into binary variables according to a threshold T. Sex is a categorical variable (+1 indicates a female, −1 male), while BMI, age, and genomic PCs are continuous variables. The choice of T reflected the type of information a binary value captured about the corresponding continuous molecular feature. For age, T was set to 30 for both cohorts. For BMI, the threshold was set to the median value 23.62 kg m^−2^ and 23.78 kg m^−2^ for the GLOBAL and EURO cohort, respectively. As genomic background was built using PCs on the HapMap data, T was set to zero.

GWAS identified peak SNPs were three-class variables given as AA = 0, Aa = 1, aa = 2, with A the major allele and a the minor allele (as determined based on our EURO cohort data), meaning homozygous major allele, heterozygous, and homozygous minor allele, respectively and reflecting the additive model of inheritance. Missing genotypes were ignored during the GWAS, but were assigned to the homozygous major allele class prior to subsequent analysis. The three-class variable was then converted into two binary variables following ordinal categories: the first codes 0 for AA, and 1 to Aa and aa, practically mimicking the dominant model of inheritance; the second assigns 0 to AA and Aa, 1 to aa, expressing the recessive model of inheritance. Both new two-class variables were tested for association again, and were used subsequently if at least one facial segment reached the arbitrary threshold for selecting and concatenating facial shape features of *p* ≤ 5 × 10^−5^ in the EURO cohort.

First note that multi-class variables do not necessarily have to be converted to two-class variables, but it is common practice to do so since most classifier implementations, including the support vector machines (SVM) used in this work, are designed for two opposing classes only. Other classifiers, like a linear discriminant classifier can handle multiple classes simultaneously, but were not investigated as such in this work. Alternatively, regression techniques can be used as well, but again were not investigated in this work. Also note, that the conversion of continuous information to a two-class variable is crude, and that more information could be gained by defining multiple classes across the continuous domain. For genomic background, the results did not improve substantially by adding multiple categories along each genomic PC. In this work, we downscale the emphasis on age and BMI compared to the other molecular features used, and did not further optimize the categorization of these aspects as such. However, we expect further improvements to be gained when a finer resolution of age and BMI comparison is in place.

A face-to-DNA classifier labels faces into categories of a molecular feature, such as sex (two-class variable; male versus female), genomic PCs (continuous variables), and individual SNPs (three-class genotype variables) or categories of DNA-inferable features such as age and BMI (continuous variables). Briefly, a face-to-DNA classifier labels new instances, knowing the class labels of training observations with measurements on different variables, called predictors or features. In this work, the input to a classifier were concatenated shape features from selected facial segments and the output a genotypic labelling as well as the probabilities of belonging to each of the opposing genotypic classes (p_1_ and p_2_ with p_1_ = 1-p_2_). For each identified genotypic factor, facial segments were selected according to a threshold of *p* ≤ 1 × 10^−3^ and *p* ≤ 5 × 10^−5^ in the GLOBAL and EURO cohort, respectively. These thresholds were defined as slightly less stringent, but still selective, to the FDRd thresholds that were computed for each cohort separately. A face-to-DNA classifier was then built using the function fitcsvm from Matlab^TM^ 2016b which trains a SVM for two-class classification. Briefly, a SVM finds the hyperplane that best separates the observations into classes. We trained the classifier with a Gaussian kernel using internally defined parameters in fitcsvm and observation weights as a function of class balance, to deal with possible data imbalances. The Matlab^TM^ function predict was used for new instances and generated the most likely class label as well as posterior class probabilities.

For the GLOBAL cohort we investigated a range of arbitrary thresholds to select facial segments and observed only minor fluctuations in the results. We also investigated a range of alternative classifiers to SVM that are implemented and readably available in Matlab^TM^ as well as different strategies in Matlab^TM^ to set the hyper-parameters in SVM classifiers. No real improvements were observed. All alternative results are presented in Supplementary Data 2.

Given a probe DNA-profile with specific class labels for each molecular feature, posterior class probabilities of faces classified were selected accordingly. In other words, a vector of matching scores, one for each molecular feature, was obtained for each face being classified against the probe DNA-profile. The vector of scores per participant were fused into a single overall matching score in order to perform the recognition analyses. Score fusion represents a key step in every multi-biometric system since the final decision is made upon the fused matching score. In multi-biometric systems, the score fusing is a technique that combines the matching scores from different sources to compensate for the limitations in performance of individual matchers^37^. As each molecular feature has a specific level of discrimination power, they will give a different contribution to the recognition problem. Instead of deterministically defining weights, or calculating the fused score with the sum-, max-, min-, product-rules, we learned their contribution using a classifier-based score fusion technique^11^. Scores from multiple facial matchers were treated as feature vectors, the corresponding labels as true labels; a classifier was constructed to discriminate genuine (correct face matched against DNA-profile) and impostor (incorrect face matched against DNA-profile) score vectors. Among a variety of classifiers implemented in Matlab^TM^ 2016b, we used a Naive Bayes model since it gave the best results (Supplementary Data 1). Shortly, all Naive Bayes classifiers assume that the value of a particular feature is independent of the value of any other feature, given the class variable. The fitcnb Matlab^TM^ function was used to build the Naive Bayes classifier and the command predict to generate genuine/imposter probabilities. The higher the genuine probability used as overall matching score, the more likely a face-to-DNA match is correct, the better the overall match of the face against probe DNA-profile.

In the biometric identification setup, a one-to-many comparison of probe DNA with multiple facial candidates in a gallery was performed. The identity was established by looking at the best matching candidates after sorting the gallery from highest to lowest overall matching scores. The performance was evaluated using CMC curves, which plot the cumulative identification rate as a function of rank, which is simply the position of the true candidate in the sorted gallery list. Identification performance is typically summarized with rank x% identification rate, reflecting the percentage of recognition results that are within the top x% of the sorted gallery. High identification rates and rapid relative increases in the CMC indicate better performance.

In the biometric verification setup, a one-to-one comparison with a single facial candidate is performed. The identity is verified if the overall matching score is deemed high enough. The performance was evaluated using ROC analyses. For a range of thresholds on the overall matching score, the true positive fraction (TPF) is plotted against the false positive fraction (FPF). Performance measures that are typically reported are the area under the curve (AUC) and the equal error rate (EER), which is the point on the ROC where the fractions of false accept and reject are equal. Lower EER and higher AUC scores indicate better performance. Note that this setup is the preferred evaluator for recognition that allows for a comparison of results across multiple studies^38^.

Data and open-source software to run face-to-DNA classifier, matching and fusing, and the biometric analyses on both cohorts is available, see Data and Code Availability.

### Ethics statement

We have complied with all relevant ethical regulations for work with human participants and informed consent was obtained. Institutional review board (IRB) approval was obtained at each recruitment site and all participants gave their written informed consent prior to participation; for children, written consent was obtained from a parent or legal guardian. For the Pittsburgh sample the following local ethics approvals were obtained: University of Pittsburgh IRB #PRO09060553 and #RB0405013; UT Health Committee for the Protection of Human Subjects #HSC-DB-09-0508; Seattle Children’s IRB #12107; University of Iowa Human Subjects Office/IRB #200912764 and #200710721. For the Penn State sample, the following local ethics approvals were obtained: State College, PA (IRB #44929 & #4320 New York, NY (IRB #45727); Urbana-Champaign, IL (IRB #13103); Dublin, Ireland; Rome, Italy; Warsaw, Poland; and Porto, Portugal (IRB #32341); and Twinsburg, OH (IRB #2503). To perform the analysis on the data local ethics approval at the KU Leuven was also obtained: Ethische Commisie Onderzoek UZ/KU Leuven (EC #S60568).

### Reporting summary

Further information on research design is available in the Nature Research Reporting Summary linked to this article.

## Supplementary information

Reporting Summary

Supplementary Information

Peer Review File

Description of Additional Supplementary Files

Supplementary Data 1

Supplementary Data 2

## Data Availability

The datasets generated during and/or analyzed during the current study are available in figshare with the identifier [10.6084/m9.figshare.7649024]. This includes a single zip file with all the phenotype/genotype data needed to replicate the results in this work, without the need to access the raw images or genomic information. This further includes two pdf files with additional illustrations for the genetic associations to facial shape. HapMap genomic data is available at https://www.genome.gov/10001688/international-hapmap-project/ All of the genotypic markers for the Pittsburgh Dataset are available to the research community through the dbGaP controlled access repository [http://www.ncbi.nlm.nih.gov/gap] at accession number: phs000949.v1.p1. The raw source data for the phenotypes—the 3D facial surface models in.obj format—are available through the FaceBase Consortium [www.facebase.org]. Access to these 3D facial surface models requires proper institutional ethics approval and approval from the FaceBase data access committee. Additional details can be requested from S.M.W [smwst46@pitt.edu]. The participants comprising the Penn State University dataset were not collected with broad data sharing consent. Given the highly identifiable nature of both facial and genomic information and unresolved issues regarding risk to participants, we opted for a more conservative approach to participant recruitment. Broad data sharing of these collections would thus be in legal and ethical violation of the informed consent obtained from the participants. This restriction is not because of any personal or commercial interests. Additional details and a more confined sharing can be requested from M.D.S [mds17@psu.edu].

## Code availability

The spatially-dense facial mapping software, is available free of use for academic purposes. This software referred to as MeshMonk^39^ is hosted at https://github.com/TheWebMonks/meshmonk. All custom Matlab^TM^ implementations and scripts, used in this work for the hierarchical spectral clustering based facial phenotyping, the construction of shape PCs, the statistical analysis, the classification as well as the regression methodology, the score fusing and biometric evaluations demonstrated on the de-identified datasets and 3D shape features used in this work are available in figshare with the identifier [10.6084/m9.figshare.7649024]. These implementations have been tested using Matlab^TM^ versions 2016b, 2017a&b, 2018a and are available under the Apache 2.0 License

## References

[1] Kayser M (2015). Forensic DNA phenotyping: predicting human appearance from crime scene material for investigative purposes. Forensic Sci. Int. Genet..

[2] Gill P, Jeffreys AJ, Werrett DJ (1985). Forensic application of DNA ‘fingerprints’. Nature.

[3] Roewer L (2013). DNA fingerprinting in forensics: past, present, future. Investig. Genet..

[4] Roosenboom J, Hens G, Mattern BC, Shriver MD, Claes P (2016). Exploring the Underlying genetics of craniofacial morphology through various sources of knowledge. Biomed. Res. Int..

[5] Claes P, Hill H, Shriver MD (2014). Toward DNA-based facial composites: preliminary results and validation. Forensic Sci. Int. Genet..

[6] Lippert C (2017). Identification of individuals by trait prediction using whole-genome sequencing data. Proc. Natl Acad. Sci. USA.

[7] Claes P (2018). Genome-wide mapping of global-to-local genetic effects on human facial shape. Nat. Genet..

[8] Hayton JC, Allen DG, Scarpello V (2004). Factor retention decisions in exploratory factor analysis: a tutorial on parallel analysis. Organ. Res. Methods.

[9] Sero, D. et al. Facial recognition from DNA using face-to-DNA classifiers. figshare, 10.6084/m9.figshare.7649024 (2019). 10.1038/s41467-019-10617-y PMC6560034 31186421

[10] Gibbs RA (2003). The international HapMap project. Nature.

[11] Ross, A. A., Jain, A. K. & Nandakumar. *Handbook of Multibiometrics* (Springer-Verlag, Boston 2006).

[12] Kayser M, Schneider PM (2009). DNA-based prediction of human externally visible characteristics in forensics: motivations, scientific challenges, and ethical considerations. Forensic Sci. Int. Genet..

[13] Erlich Y, Narayanan A (2014). Routes for breaching and protecting genetic privacy. Nat. Rev. Genet..

[14] O’Toole AJ, Vetter T, Troje NF, Bülthoff HH (1997). Sex classification is better with three-dimensional head structure than with image intensity information. Perception.

[15] Xia B, Ben Amor B, Daoudi M (2017). Joint gender, ethnicity and age estimation from 3D faces: an experimental illustration of their correlations. Image Vis. Comput.

[16] Huang D (2014). Local circular patterns for multi-modal facial gender and ethnicity classification. Image Vis. Comput.

[17] Skorska MN, Geniole SN, Vrysen BM, McCormick CM, Bogaert AF (2015). Facial structure predicts sexual orientation in both men and women. Arch. Sex. Behav..

[18] Dantcheva A, Elia P, Ross A (2015). What else does your biometric data reveal? A survey on soft biometrics. Trans. Inf. Forensics Secur. Inst. Electr. Electron. Eng..

[19] Abate AF, Nappi M, Riccio D, Sabatino G (2007). 2D and 3D face recognition: a survey. Pattern Recognit. Lett..

[20] Smeets D, Claes P, Hermans J, Vandermeulen D, Suetens P (2012). A comparative study of 3-D face recognition under expression variations. IEEE Trans. Syst. Man, Cybern. Part C (Appl. Rev..

[21] Phillips PJ, O’Toole AJ (2014). Comparison of human and computer performance across face recognition experiments. Image Vis. Comput.

[22] White D, Phillips PJ, Hahn CA, Hill M, O’Toole AJ (2015). Perceptual expertise in forensic facial image comparison. Proc. Biol. Sci..

[23] Galdi C, Dugelay J-L (2017). FIRE: fast Iris REcognition on mobile phones by combining colour and texture features. Pattern Recognit. Lett..

[24] Hill H (2011). How different is different? Criterion and sensitivity in face-space. Front. Psychol..

[25] Erlich, Y. Major flaws in Identification of individuals by trait prediction using whole-genome. Preprint at https://www.biorxiv.org/content/10.110 (2017).

[26] Jackson, A. S., Bulat, A., Argyriou, V. & Tzimiropoulos, G. Large pose 3D face reconstruction from a single image via direct volumetric CNN regression. In *Proc.**2017 IEEE International Conference on Computer Vision (ICCV)* 1031–1039, 10.1109/ICCV.2017.117 (IEEE, 2017).

[27] Broad Ellen. Who gets held accountable when a facial recognition algorithm fails? http://ellenbroad.com/facial-recognition-who-gets-held-accountable/ (2017).

[28] Klare, B. F., Burge, M. J., Klontz, J. C., Bruegge, R. W. V. & Jain, A. K. Face recognition performance: role of demographic information. *IEEE Trans. Inf. Forensics Secur*. **2**, 1789–1801 (2012).

[29] Gibbs RA (2015). A global reference for human genetic variation. Nature.

[30] Nelson MR (2008). The Population Reference Sample, POPRES: a resource for population, disease, and pharmacological genetics research. Am. J. Hum. Genet..

[31] Behar DM (2007). The genographic project public participation mitochondrial DNA database. PLoS Genet..

[32] Turner Lee N (2018). Detecting racial bias in algorithms and machine learning. J. Inf., Commun. Ethics Soc..

[33] Wagner, J. K. DNA, racial disparities, and biases in criminal justice: searching for solutions. Alb. LJ Sci. & Tech., 27, p.95. (2017) (Albany Law Journal of Science and Technology).

[34] Laurie CC (2010). Quality control and quality assurance in genotypic data for genome-wide association studies. Genet. Epidemiol..

[35] Klingenberg CP (2008). Morphological integration and developmental modularity. Annu. Rev. Ecol. Evol. Syst..

[36] Benjamini, Y. & Yekutieli, D. The control of the false discovery rate in multiple testing under dependency. *Ann. Stat*. **29**, 1165–1188 (2001).

[37] Nandakumar K, Chen Yi, Dass SC, Jain AK (2008). Likelihood ratio-based biometric score fusion. IEEE Trans. Pattern Anal. Mach. Intell..

[38] Phillips PJ (2012). The good, the bad, and the ugly face challenge problem. Image Vis. Comput..

[39] White JD (2019). MeshMonk: open-source large-scale intensive 3D phenotyping. Sci. Rep..

