# Supplementary material for: Facial recognition from DNA using face-to-DNA classifiers
Source: Nat Commun. 2019 Jun 11;10:2557. doi: 10.1038/s41467-019-10617-y (PMC6560034; doi:10.1038/s41467-019-10617-y)
Supplement: Supplementary file 2 — Supplementary Information [file 41467_2019_10617_MOESM2_ESM.pdf]

Facial Recognition from DNA using face-to-DNA classifiers

Sero et al.

Supplementary Information

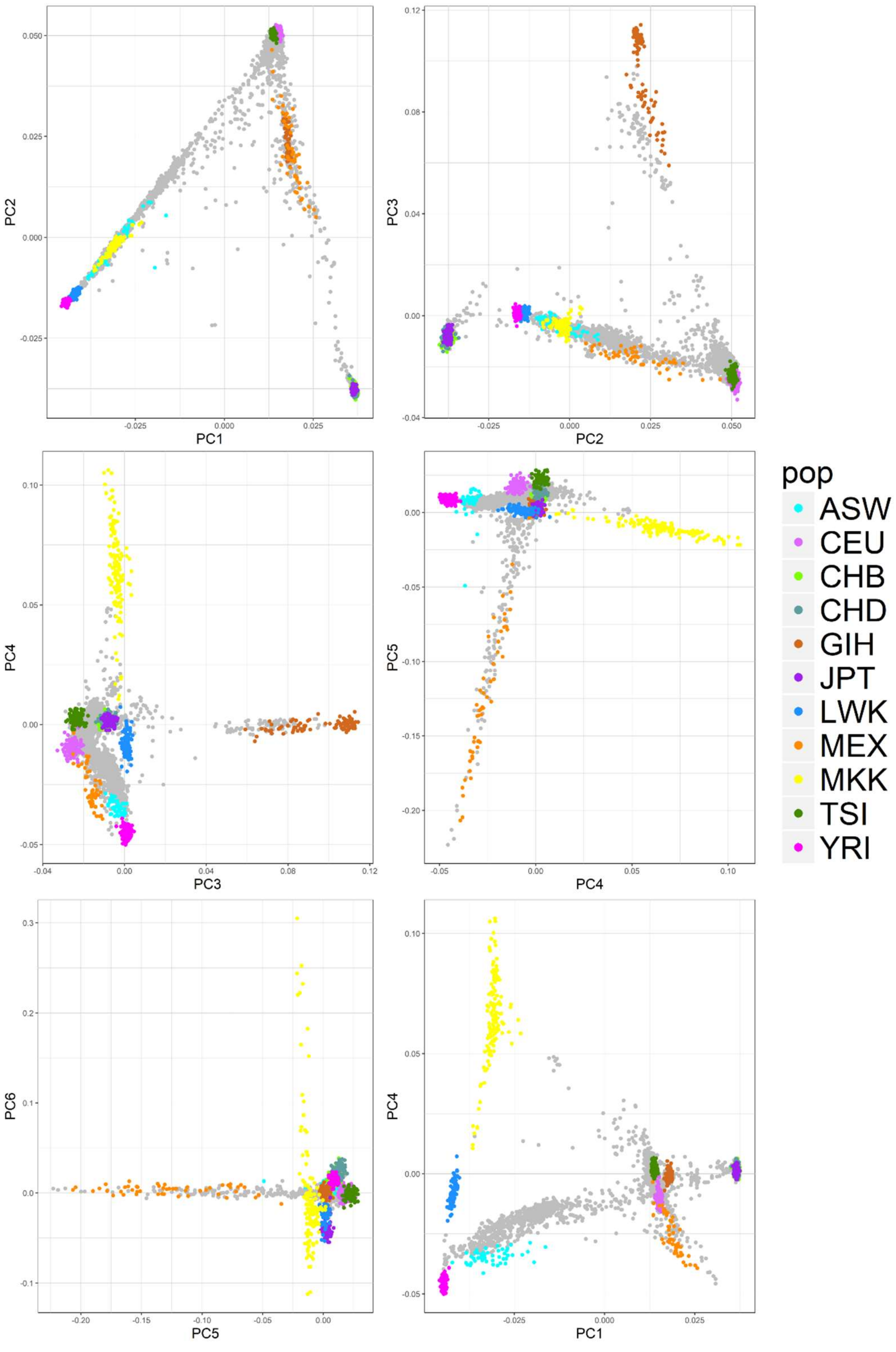

Supplementary Figure 1. HapMap principal component (PC) plots showing genetic structure in the GLOBAL cohort (grey points). Samples from the HapMap project[1] are colored. It is observed, that the GLOBAL cohort is genetically diverse and highly admixed. YRI: Yorubans from Ibadan; MKK: Masai from Kenya; LWK: Luhya from Kenya; CEU: Utah residents of Northern and Western European ancestry; TSI: Italians from Tuscany; CHB: Han Chinese from Beijing; JPT: Japanese from Tokyo; CHD: Han Chinese living in Denver; GIH: Gujarati Indians from Houston; MEX: Mexicans from the Southwest; ASW: African Americans from the Southwest.

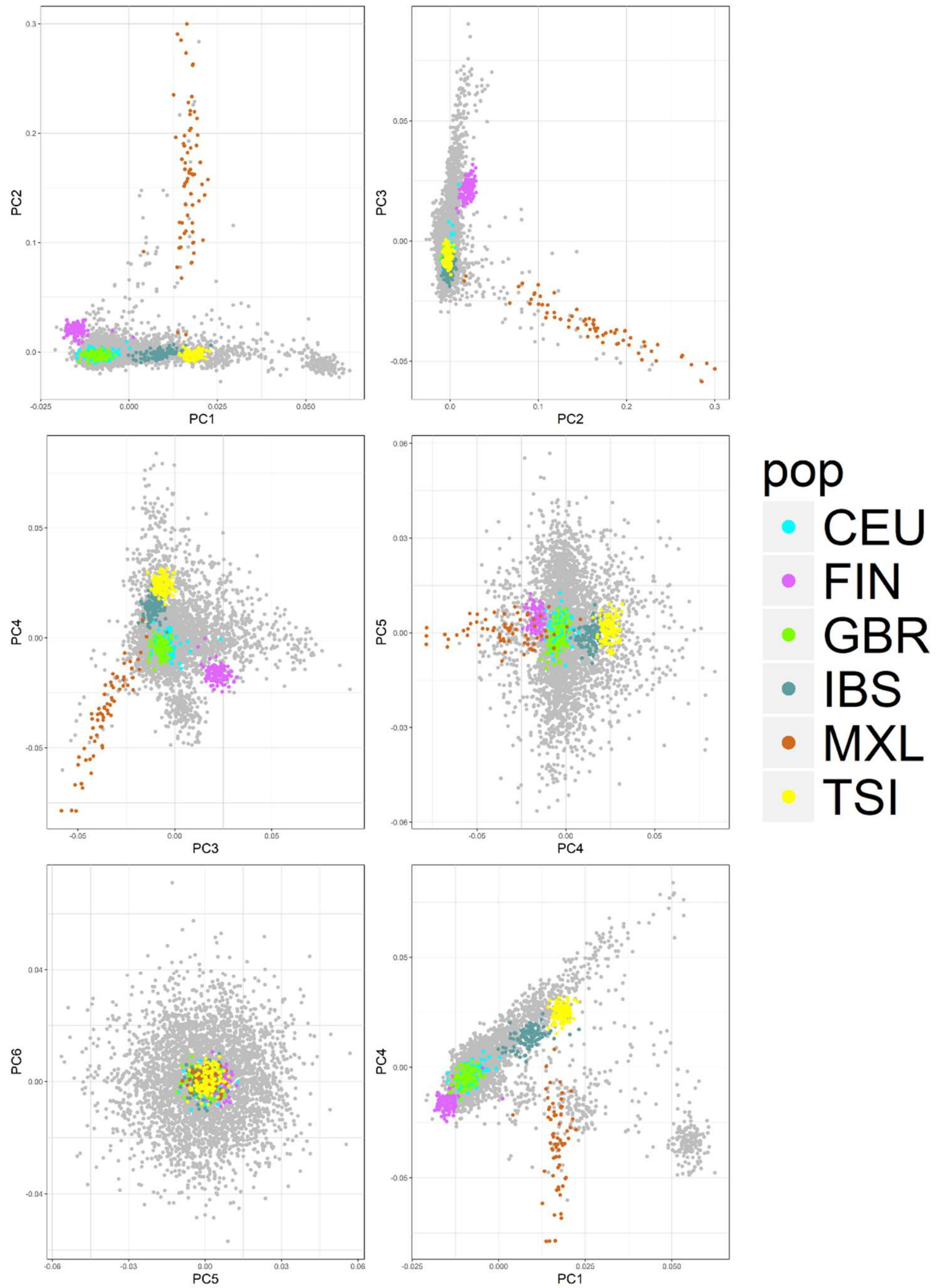

Supplementary Figure 2. EURO cohort principal component (PC) construct (PC1 up until PC6) with relevant 1000 Genome population samples projected in. Plot showing genetic structure of the EURO cohort (grey points) alongside samples of European ancestry from the 1000 genomes project (colored points) projected into the space. CEU: Utah residents with Northern and Western European ancestry; FIN: Finnish from Finland; GBR: British from England and Scotland; IBS: Iberians from Spain; TSI: Italians from Tuscani; MXL: Mexicans from Los Angeles, USA. The MXL were included to tease apart the Native American ancestry component in Hispanic individuals in the EURO cohort. Note, the 1000 Genome samples, are used solely to illustrate here and have not been used throughout the analysis in the main manuscript. Bottom right panel, PC1 vs PC4, which are the two dimensions that tested significantly against facial variations in the EURO cohort, and that therefore drive the genomic background results for this cohort. Note that PC1 and PC4, are not just driven by Hispanic individuals in contrast to PC2.

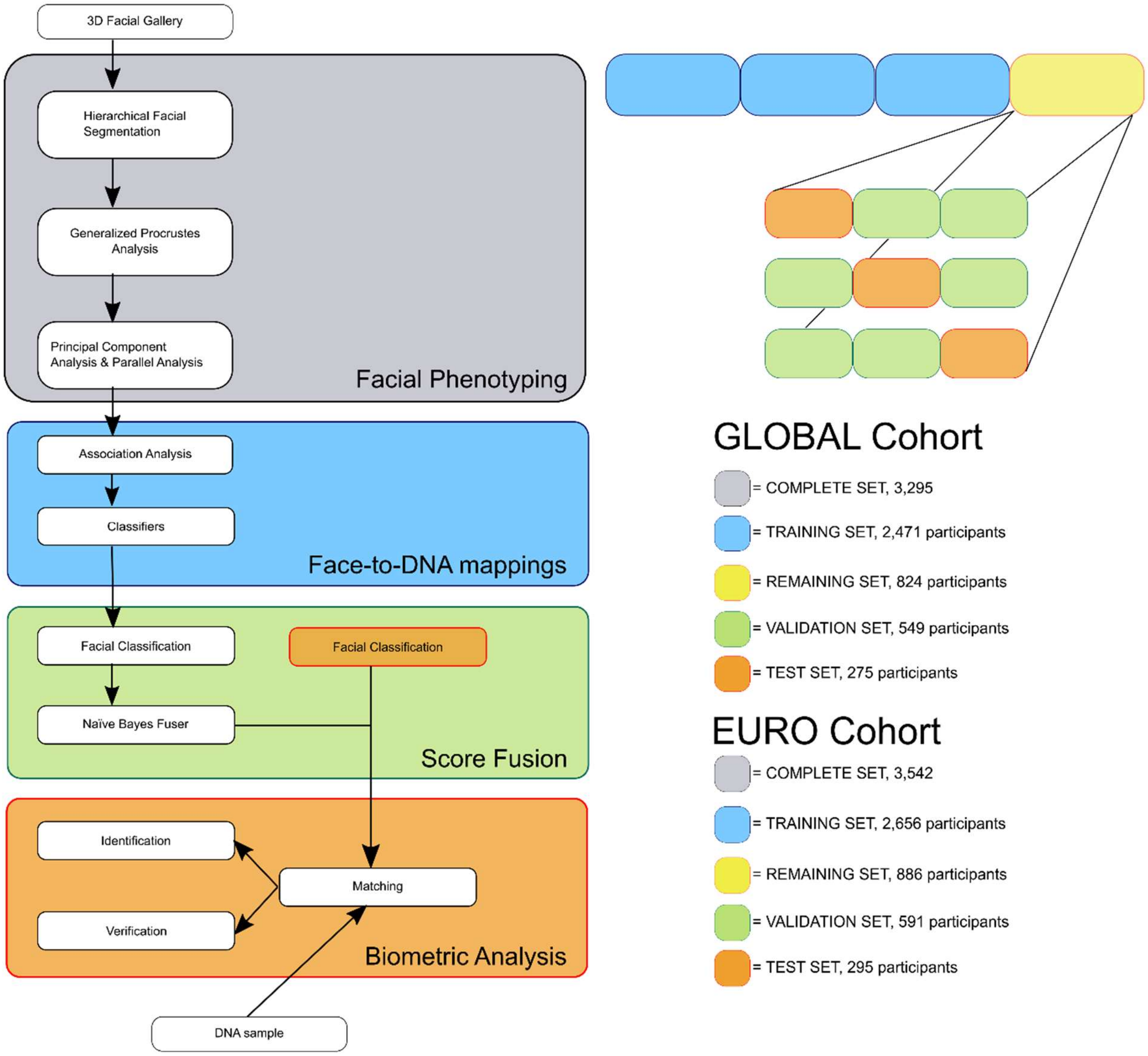

Supplementary Figure 3. Schematic overview of methods and data partitioning. Left, starting from the top: the complete set of participants was used for a hierarchical phenotyping approach that segments 3D facial shape into multiple layers of information; subsequently, facial features were obtained by first applying Generalized Procrustes Analysis separately to the quasi-landmarks comprising each facial segment, followed by PCA. Using the training set only, we performed a series of association studies to test the possible link of genetic features with the shape information. Subsequently, face-to-DNA classifiers were built and each of them labelled facial images into possible categories of a specific genetic feature of interest (sex, Age, body mass index, genetic principal components, and individual single nucleotide polymorphisms). Multiple matching scores for different associated genetic features were combined into a single overall matching score using a classification-based multi-biometric fusing system: the validation set was used to train the fuser, which was then applied to new test instances. Finally, we performed standard biometric analysis comprising identification and verification setup in order to test our ability to classify faces in the context of multiple genetic features. Right, each cohort was randomly partitioned into training, validation, and test sets. First, we created non-overlapping training and remaining sets. The training set, being the majority (75%) of the data. The remaining set was further partitioned randomly into three non-overlapping folds: two folds combined constituted the validation set, and the third remaining fold constituted the test set. This was done three times, such that each fold was used as test set once, while the other two folds were used as validation set. This generated three runs of results

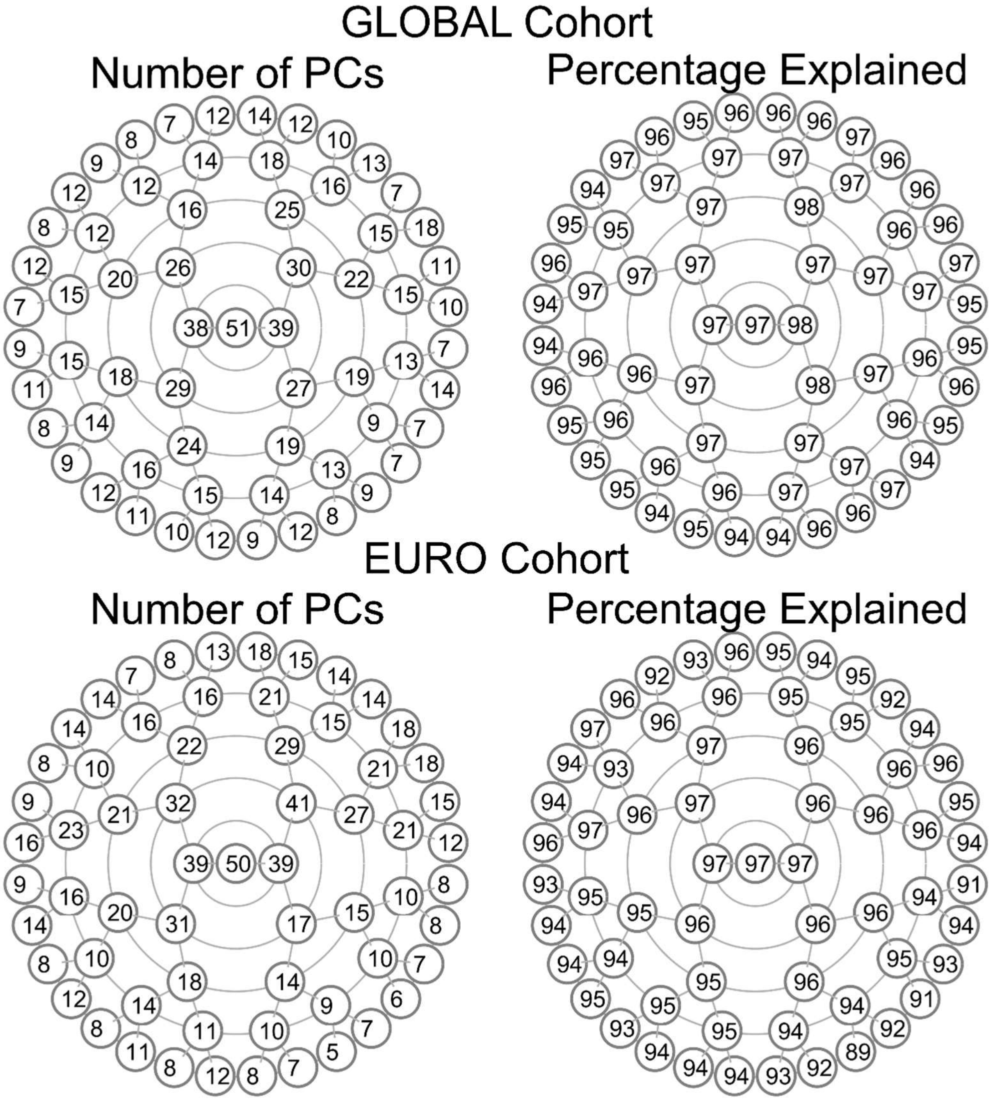

Supplementary Figure 4. Number of principal components (PCs) and variation explained per facial segment.

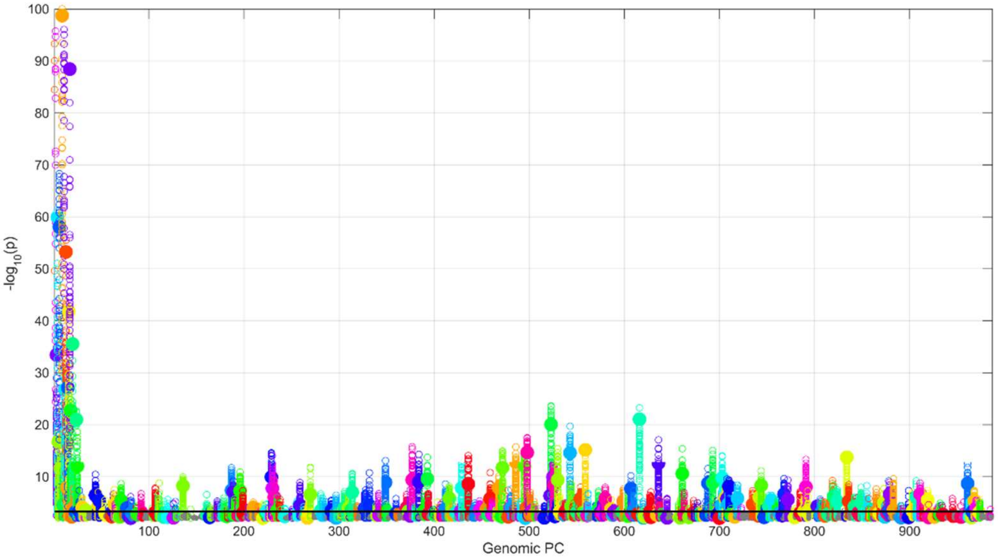

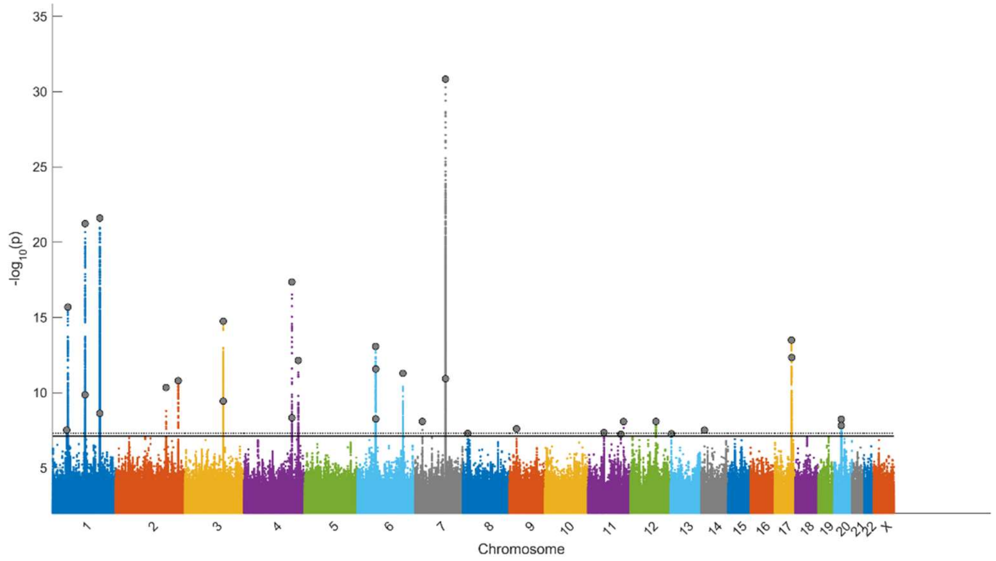

Supplementary Figure 5. Manhattan plots. Top, –log10 of the statistical evidence (p-value) over all 63 facial segments combined for all 987 genomic principal components tested in a Manhattan plot-like fashion. Bigger solid dots represent the –log10(p-value) for the full facial segment. The colors are random and have no specific meaning. Bottom, Manhattan plot of all 63 facial segments combined illustrating the chromosomal position of the 32 loci (grey dots highlight Peak SNPs, see methods for their definition) discovered in the EURO cohort. The bottom horizontal line (thick grey) represents the FDRd threshold ($p \leq 7.7 \times 10^{-8}$), and the top horizontal line (thin dotted grey) represents genome-wide significance ($p \leq 5 \times 10^{-8}$). The correspondence of the 32 loci is indicated by color, ordered by chromosome number, left to right.

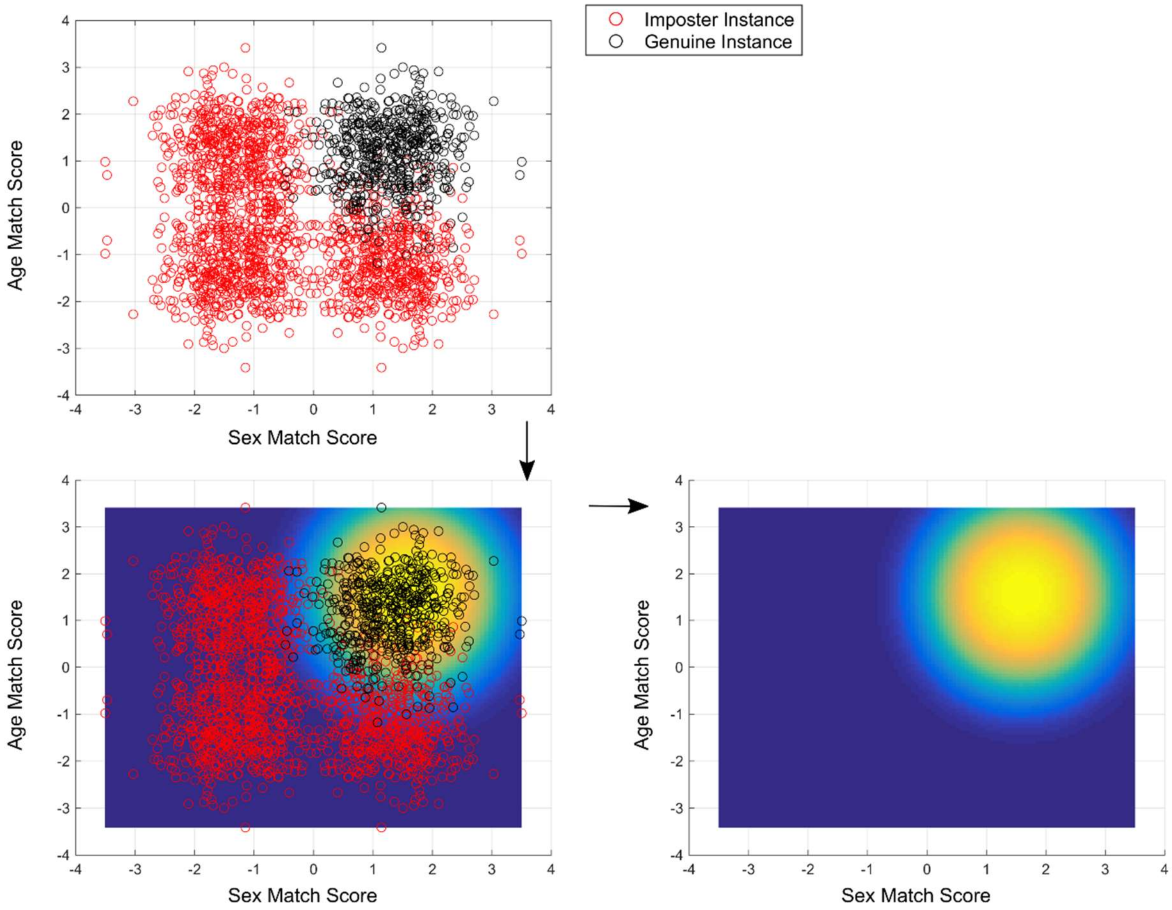

Supplementary Figure 6. Illustration of fusing matching scores. Pairwise face-to-DNA matches from the validation datasets generate two-dimensional matching vectors, one dimension for sex (x-axis) and another for age (y-axis). If a face is matched to its own DNA profile, a genuine instance (black labels) is obtained. If a face is matched to another person's DNA profile, an imposter instance (red labels) is created. If a red label overlaps with a black label in the two-dimensional scoring space, the red label is removed, prior to learning a naïve Bayes classifier. Genuine instances occupy a specific subspace (upper left quadrant, where both sex and age match (higher positive value), and the naïve Bayes classifier, explicitly delineates this subspace, by generating a higher probability value for instances lying in this subspace (blue represents a low probability (p=0) and yellow represents a high probability (p=max) of being a genuine instance). Adding remaining aspects increases the dimensionality of the scoring space, and separates genuine from imposter matches more specifically.

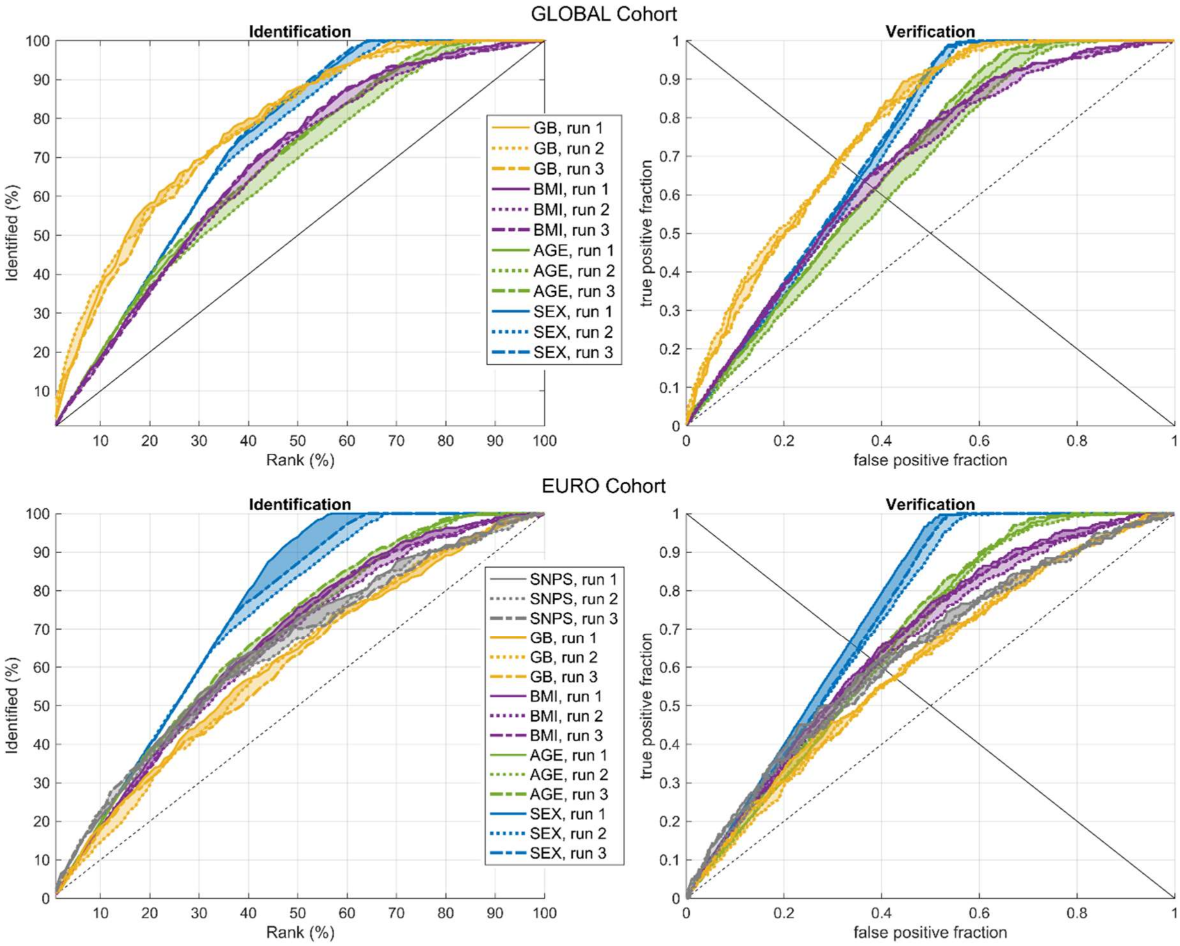

Supplementary Figure 7. Biometric evaluation per molecular aspect. Cumulative matching characteristic (CMC) curves (Left) and Receiver operating characteristic (ROC) curves (right) of each genetic aspect separately for the GLOBAL and EURO cohort, respectively. Genetic aspects for the GLOBAL cohort are sex, genomic background (GB), body mass index (BMI) and age. The curve for genomic background refers to the 382 genomic principal components (PCs). The list of genetic aspects for the EURO cohort include sex, individual single nucleotide polymorphisms (SNPs) (32), genomic background (2 PCs), BMI and age.

Supplementary Table 1. Summary results of sex, age and BMI for both cohorts. CC: canonical correlation; CC2: canonical correlation squared; DF1: degrees of freedom numerator; DF2: degrees of freedom denominator; -1: fraction of people assigned the value -1; 1: fraction of people assigned the value 1.

	PROPERTIES					EFFECT		FACE-TO-DNA CLASSIFIER			
**ASPECT**	**CC**	**CC2**	**DF1**	**DF2**	**p-value**	**quadrant**	**segment**	**threshold**	**unit**	**-1**	**1**
**GLOBAL COHORT**											
SEX	0.88	0.77	51	2419	0	0	1	0	a.n.	0.34	0.66
AGE	0.78	0.61	51	2419	0	0	1	30	years	0.75	0.25
BMI	0.56	0.32	51	2419	3.8E-161	0	1	23.62	kg/m^2	0.50	0.50
**EURO COHORT**											
SEX	0.89	0.79	50	2605	0	0	1	0	n.a.	0.35	0.65
AGE	0.69	0.48	50	2605	0	0	1	30	years	0.73	0.27
BMI	0.57	0.32	50	2605	1.1E-183	0	1	23.78	kg/m^2	0.50	0.50

Supplementary Table 2. Additional details of 32 genetic loci identified using a GWAS on the EURO training data set. SNP, single nucleotide polymorphism; Chr., Chromosome; CC, canonical correlation; CC2 canonical correlation squared; DF1 degrees of freedom numerator; DF2 degrees of freedom denominator. Quadrant and Module number as given in Figure 3 of the facial segment associated most strongly.

Locus	Chr.	Position	SNP	Candidate Gene	Quadrant	Module	CC	CC2	DF1	DF2
1p32.2	1	57048961	rs2404983	*PLPP3*	2	41	0.15	0.02	14	2637
1p32.1	1	61020499	rs4916071	intergenic	2	41	0.20	0.04	14	2640
1p12	1	119564215	rs200100774	*WARS2*	0	1	0.23	0.05	50	2565
1p12	1	119643820	rs61808932	*WARS2*	4	31	0.24	0.06	21	2617
1q31.3	1	197343295	rs949977	*CRB1*	3	24	0.15	0.02	10	2639
1q31.3	1	197343950	rs2821107	*CRB1*	3	12	0.23	0.05	14	2614
2q31.1	2	177111819	rs970797	*MTX2* (or *HOXD* cluster)	2	5	0.20	0.04	31	2624
2q36.1	2	223030502	rs1370926	*PAX3*	2	41	0.17	0.03	14	2624
3q21.3	3	127961305	rs2955084	*EEFSEC*	2	5	0.20	0.04	31	2600
3q21.3	3	128106267	rs2977562	*EEFSEC* (or *DNAJB8*)	2	11	0.20	0.04	18	2596
4q31.3	4	154820806	rs10020603	*RNF175* (or *TLR2*)	2	5	0.24	0.06	31	2604
4q31.3	4	154831619	rs17299889	*RNF175* (or *TLR2*)	2	5	0.19	0.04	31	2612
4q34.1	4	174462975	rs1059045	*NBLA00301* (or *HAND2*)	3	12	0.19	0.03	14	2534
6p21.1	6	44681257	rs227832	*BX647715* (or *SUPT3H*)	2	43	0.18	0.03	12	2643
6p21.1	6	45220175	rs9395084	*SUPT3H* (or *RUNX2*)	2	5	0.19	0.04	31	2603
6p21.1	6	45256286	rs73735344	*SUPT3H* (or *RUNX2*)	2	43	0.17	0.03	12	2599
6q23.2	6	133609328	rs402020	*EYA4*	4	30	0.18	0.03	15	2588
7p21.1	7	18741367	rs1178103	*HDAC9*	2	41	0.16	0.02	14	2629
7q21.3	7	96124975	rs10238953	*C7orf76 (*or *SHFM1)*	3	24	0.25	0.06	10	2616
7q21.3	7	96308943	rs2272224	*SHFM1*	3	13	0.18	0.03	15	2629
8p23.1	8	8114141	rs2980419	*FAM86B3P (*or *SGK223)*	2	5	0.19	0.03	31	2595
9p22.2	9	16619529	rs13290470	*BNC2*	0	1	0.22	0.05	50	2605
11p11.2	11	47385400	rs150863859	*SPI1*	1	39	0.16	0.03	16	2589
11q22.3	11	103900016	rs7930466	*PDGFD*	3	25	0.14	0.02	9	2642
11q23.2	11	113875575	rs7925936	*HTR3A (*or *ZBTB16)*	1	38	0.15	0.02	9	2521
12q21.31	12	85577001	rs7966105	*LRRIQ1*	0	1	0.22	0.05	50	2601
13q12.11	13	22449588	rs2985662	*LINC00424*	2	45	0.15	0.02	11	2615
14q12	14	30426467	rs143974562	*PRKD1*	3	13	0.16	0.03	15	2565
17q24.3	17	69128981	rs72866756	*BC039327*	2	10	0.20	0.04	20	2624
17q24.3	17	70029448	rs11871949	*D43770 (or AK094963)*	2	10	0.20	0.04	20	2600
20p11.22	20	21628942	rs2424392	*PAX1 (*or *Nkx2_2as)*	2	40	0.15	0.02	9	2634
20p11.22	20	21758674	rs6035946	*PAX1*	1	36	0.16	0.02	14	2575

Supplementary Note 1: Supplementary analysis on DNA facial phenotyping

DNA facial phenotyping followed by face-to-face matching provides an alternative method to establish the identity of a probe DNA with unknown identity against facial images with known identity. Such a strategy was recently reported in the work of Lippert et al.[2] in which multiple phenotypes (facial shape and color, sex, age, height, weight, BMI, skin and eye color, ancestry and voice) are estimated from DNA profiles and subsequently matched against corresponding phenotypes in a database with known identities. Therefore, of particular interest in comparison to our work is the performance of DNA based facial phenotyping followed by face-to-face matching in a biometric evaluation.

DNA facial phenotyping: Following the work of Lippert et al., we implemented a series of facial prediction models to reconstruct the full facial shape of individuals (facial segment 1 in Figure 3), represented as shape PCs (GLOBAL cohort: 51 shape PCs, EURO cohort: 50 shape PCs, see Supplementary Figure 4), from a set of predictors (Sex, Age, BMI, genomic PCs and/or individual SNPs). Age, BMI, and genomic PCs were not converted to binary variables and were used unconverted as continuous predictors. For each of the shape PCs, using the training set of each cohort, a regularized linear regression model using a 10-fold cross-validation and the elastic net method with Alpha = 0.75 (Matlab^{TM} function: lasso) was trained. The 10-fold cross-validation served to tune the Lambda regularization parameter in the regression model. Facial predictions for the validation and/or test sets of each cohort were subsequently obtained by applying the regression models for each of the shape PCs, the combination of which constituted a multidimensional facial prediction. Note that in the work of Lippert et al. the results reported were based on a ridge instead of a lasso regression. However, they investigated several regression models, including a Lasso regression, with little impact on the results for facial prediction.

Face-to-Face matching: Matching scores between faces predicted and faces observed in the larger dataset were obtained by a cosine distance between facial shape PCs represented as vectors and by supervised metric learning resulting in a weighted distance between facial shape PCs. For the latter, the validation set of each cohort was used to determine an optimal weight for each of the shape PCs using Linear Discriminant Analysis (LDA) that maximizes the separation of supervised genuine versus imposter face-to-face pairwise combinations. Subsequently, the matching scores between faces predicted and faces observed in the test set of each cohort were subjected to the biometric identification and verification tasks.

In this supplement, we first use the EURO cohort with sex, age, BMI and the first and fourth Genomic PC as predictors for facial shape, to investigate the two different face-to-face distances as matching scores in a biometric evaluation, followed by the effect of increasing the amount of genomic PCs and adding individual genetic loci as predictors for facial shape. Then we report on the results for the GLOBAL cohort.

Comparison of cosine and weighted distance for face-to-face matching: The cosine distance and a metric learning based (using YASMET instead of LDA) weighted distance were also compared in the work of Lippert et al., with the latter outperforming the former. In the work of Lippert et al., shape PCs were analyzed together with color PCs and values of sex, age and ancestry predicted from faces. Therefore, we analyzed both types of distances as matching scores

for shape PCs only and for shape PCs augmented with the predictions of sex, age, BMI and genomic PCs from faces using the respectively trained facial classifiers in this work. The results for the cosine distance and weighted distance are given in Supplementary Figure 8 and Supplementary Figure 9, respectively. The summary statistics for both distances, across the three test sets are given in Supplementary Table 3.

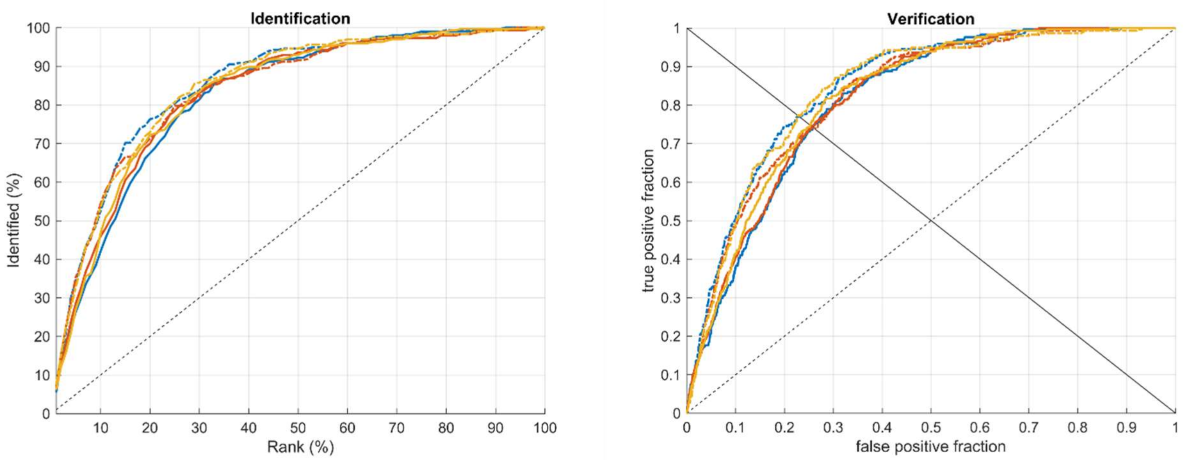

Supplementary Figure 8. Identification and verification results for the EURO cohort using the cosine distance as face-to-face matching score. Solid lines, results for shape principal components (PCs) only. Dash-dotted lines, results for shape PCs augmented with estimations of sex, age, body mass index, and genomic PCs from the face. Blue color, test dataset 1, orange color, test dataset 2, yellow color, test dataset 3.

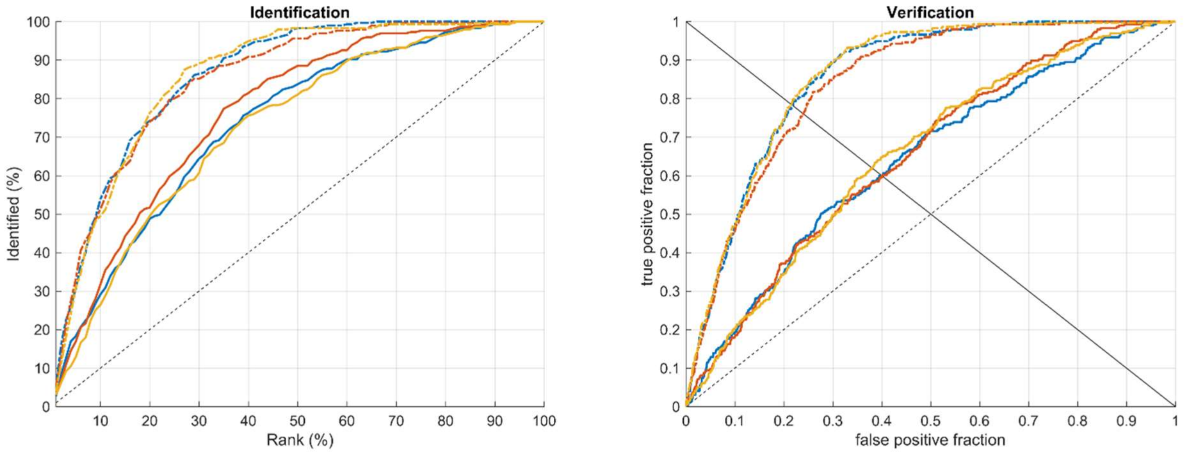

Supplementary Figure 9. Identification and Verification results EURO cohort using the weighted distance as face-to-face matching score. Solid lines, results for shape PCs only. Dash-dotted lines, results for shape PCs augmented with estimations of sex, age, BMI, and genomic PCs from the face. Blue color, test dataset 1, orange color, test dataset 2, yellow color, test dataset 3. PCs, principal components; BMI, body mass index.

information	EER	σ	AUC	σ	R1 (%)	σ	R10 (%)	σ	R20 (%)	σ
Cosine shape PCs	0.256	0.008	0.818	0.006	5.643	1.408	44.356	2.341	68.623	2.543
Cosine shape PCs + Sex, Age, BMI, GB	0.239	0.018	0.840	0.010	7.111	0.350	53.498	0.926	73.479	2.547
Metric learning shape PCs	0.394	0.011	0.648	0.006	2.934	0.193	29.117	2.658	49.998	1.503
Metric learning shape PCs + Sex, Age, BMI, GB	0.224	0.014	0.853	0.009	4.740	0.669	51.580	2.212	74.832	1.253

Supplementary Table 3. Average identification and verification results over the three test runs. EER, verification equal error rate; AUC, verification area under the curve; R1, rank 1% identification rate; R10 rank 10% identification rate; R20 rank 20% identification rate; σ standard deviation. Random performance is given as EER=0.5, AUC=0.5, R1=1%, R10 = 10%, R20 = 20%. % refers to the percentage of individuals in the gallery (EURO = 295, the test datasets). PCs, principal components; BMI, body mass index; GB, genomic background or genomic PCs.

When using shape PCs only, it is observed that the cosine distance is significantly better than the weighted distance. When using shape PCs augmented with predictions of sex, Age, BMI and genomic PCs, it is observed that the performance of both distances increases. The performance of the weighted distance increases substantially, and is now slightly better than the cosine distance, which is conform the results reported in Lippert et al. Based on these results, and since we are interested in comparing our approach using face-to-DNA classifiers only against DNA-to-face regressions only, the cosine distance between shape PCs is opted for.

European genomic PCs: In our work, the investigation of genomic PCs in the EURO cohort was mainly restricted to those selected following a GWAS paradigm to control for population stratification. Therefore, we mainly investigated the first four genomic PCs, of which only the first and the fourth showed a good association to facial shape. However, for regression based facial predictions as done in the work of Lippert et al., up to 1000 genomic PCs were incorporated for a heterogeneous dataset like our GLOBAL cohort. Therefore, we investigated if working with 1000 genomic PCs in the EURO cohort potentially improves the regression based facial predictions. The results are visualized in Supplementary Figure 10 and the summary statistics across the three test sets are given in Supplementary Table 4.

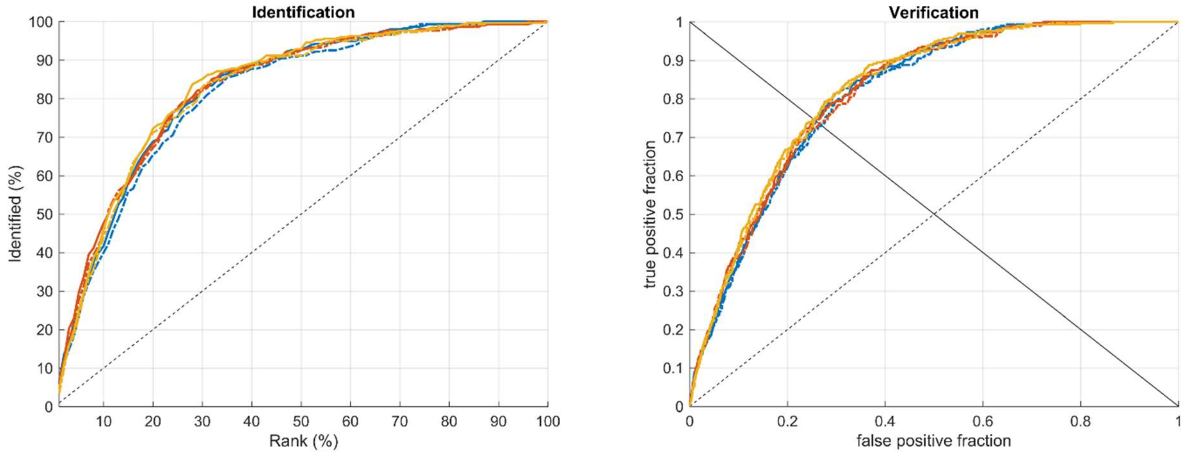

Supplementary Figure 10. Identification and Verification results EURO cohort using two sets of predictors. Solid lines, results for X = [Sex, Age, BMI, Genomic $PCs_{1,4}$]. Dash-dotted lines, results for X = [Sex, Age, BMI, Genomic PCs_{1-1000}]. Blue color, test dataset 1, orange color, test dataset 2, yellow color, test dataset 3. PCs, principal components; BMI, body mass index;

information	EER	σ	AUC	σ	R1 (%)	σ	R10 (%)	σ	R20 (%)	σ
X = [Sex,Age,BMI,Genomic $PC_{1,4}$]	0.256	0.008	0.818	0.006	5.643	1.408	44.356	2.341	68.623	2.543
X = [Sex,Age,BMI,Genomic PC_{1-1000}]	0.264	0.005	0.813	0.005	5.306	1.098	43.901	3.313	67.832	2.460

Supplementary Table 4. Average identification and verification results over the three test runs. EER, verification equal error rate; AUC, verification area under the curve; R1, rank 1% identification rate; R10 rank 10% identification rate; R20 rank 20% identification rate; σ standard deviation. Random performance is given as EER=0.5, AUC=0.5, R1=1%, R10 = 10%, R20 = 20%. % refers to the percentage of individuals in the gallery (EURO = 295, the test datasets). PCs, principal components; BMI, body mass index;

These results indicate that adding more genomic PCs in the EURO cohort does not improve or change the results compared to using only the ones (Genomic PC 1 and 4) we selected in our work as strongly associated to facial shape. In contrast to the GLOBAL cohort, the EURO cohort is a homogenous population sample, and global ancestry estimations using genomic PCs are therefore mainly restricted to deal with population stratification.

Individual genetic loci: The main contribution of our work using face-to-DNA classifiers involves the ability to incorporate individual genetic loci throughout the genome in improving the biometric outcomes. Doing so, moves from the identification of an individual's population background to the identification of an individual within a single homogenous population. Here we investigated if such an improvement could also be obtained using the regression based DNA-to-Face prediction strategy, by adding the 32 Peak SNPs under the additive genetic model (AA=0, Aa=1, aa=2) from our GWAS as additional predictors for facial shape PCs. The results are visualized in Supplementary Figure 11 and the summary statistics across the three test sets are given in Supplementary Table 5.

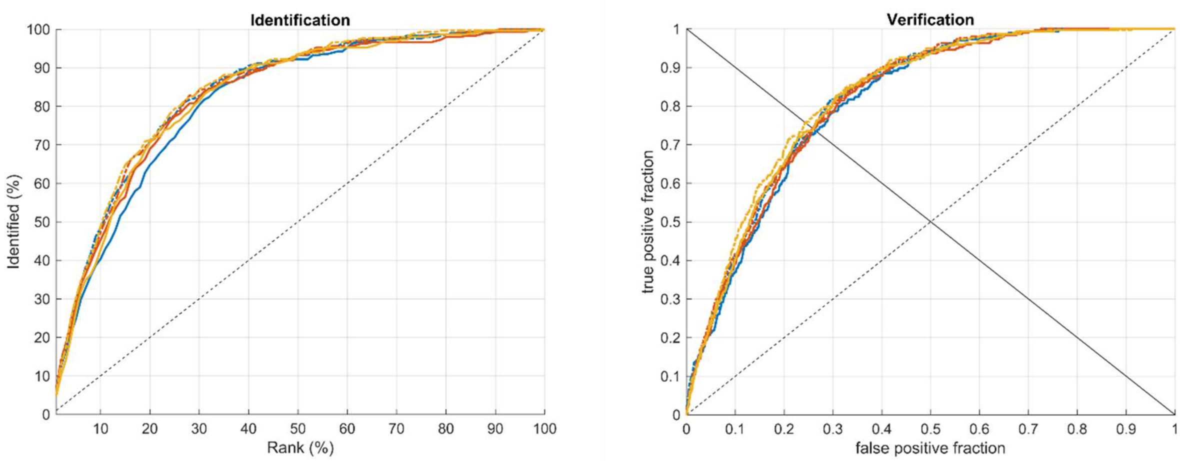

Supplementary Figure 11. Identification and verification results EURO cohort using two sets of predictors. Solid lines, results for X = [Sex, Age, BMI, Genomic $PCs_{1,4}$]. Dash-dotted lines, results for X = [Sex, Age, BMI, Genomic $PCs_{1,4}$, $SNPs_{1-32}$]. Blue color, test dataset 1, orange color, test dataset 2, yellow color, test dataset 3. PCs, principal components; BMI, body mass index;

information	EER	σ	AUC	σ	R1 (%)	σ	R10 (%)	σ	R20 (%)	σ
X = [Sex,Age,BMI,Genomic $PC_{1,4}$]	0.256	0.008	0.818	0.006	5.643	1.408	44.356	2.341	68.623	2.543
X = [Sex,Age,BMI,Genomic $PC_{1,4}$, $SNPs_{1-32}$]	0.254	0.008	0.825	0.005	6.320	0.516	48.195	0.796	70.881	0.291

Supplementary Table 5. Average identification and verification results over the three test runs. EER, verification equal error rate; AUC, verification area under the curve; R1, rank 1% identification rate; R10 rank 10% identification rate; R20 rank 20% identification rate; σ standard deviation. Random performance is given as EER=0.5, AUC=0.5, R1=1%, R10 = 10%, R20 = 20%. % refers to the percentage of individuals in the gallery (EURO = 295, the test datasets). PCs, principal components; BMI, body mass index; SNPs single nucleotide polymorphisms.

These results indicate that the addition of individual genetic loci in the prediction of faces from DNA do not contribute differentially over all biometric metrics. This is in contrast to the consistent contribution of individual SNPs on all biometric metrics as reported in Table 3 of the main manuscript. Furthermore, compared to the results reported in Table 3 of the main manuscript based on the same selection of predictors, those in Supplementary Table 5 are substantially lower. We do note that this is the case for the regression based facial prediction as implemented here. Since, in our work using face-to-DNA classifiers, the genetic loci identified contribute to the overall performance, it remains possible that yet more advanced and future facial prediction models can incorporate these loci as well. However, to date, and to the best of our knowledge on related work, such results have not been achieved yet. The same applies for the classifiers used in this work, more advanced and future facial classifiers can improve on our results, making this work a baseline for future work on DNA-to-face prediction and face-to-DNA classification for biometric scenarios.

GLOBAL cohort: Similar to the EURO cohort, we investigated the influence of using the 382 selected genomic PCs associated to facial shape against using all 987 genomic PCs available. The results are visualized in Supplementary Figure 12 and the summary statistics across the three test sets are given in Supplementary Table 6.

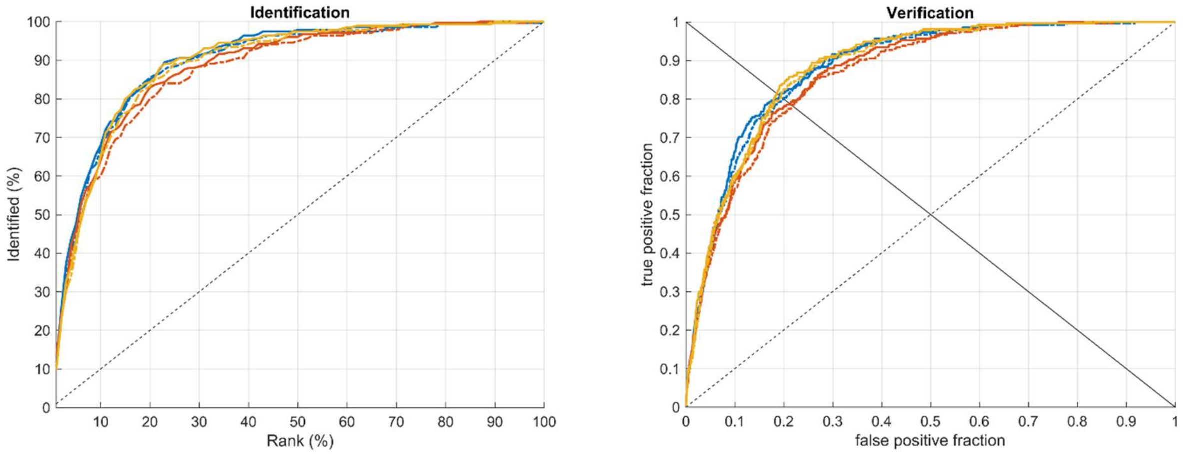

Supplementary Figure 12. Identification and verification results GLOBAL cohort using two sets of predictors. Solid lines, results for X = [Sex, Age, BMI, Genomic PCs_{1-382}]. Dash-dotted lines, results for X = [Sex, Age, BMI, Genomic PCs_{1-987}]. Blue color, test dataset 1, orange color, test dataset 2, yellow color, test dataset 3. PCs, principal components; BMI, body mass index.

information	EER	σ	AUC	σ	R1 (%)	σ	R10 (%)	σ	R20 (%)	σ
X = [Sex,Age,BMI,Genomic $PC_{1\text{-}382}$]	0.198	0.015	0.882	0.009	11.648	1.800	65.655	2.088	84.102	1.105
X = [Sex,Age,BMI,Genomic $PC_{1\text{-}987}$]	0.203	0.012	0.875	0.011	10.436	0.536	64.200	3.765	82.889	2.742

Supplementary Table 6. Average identification and verification results over the three test runs. EER, verification equal error rate; AUC, verification area under the curve; R1, rank 1% identification rate; R10 rank 10% identification rate; R20 rank 20% identification rate; σ standard deviation. Random performance is given as EER=0.5, AUC=0.5, R1=1%, R10 = 10%, R20 = 20%. % refers to the percentage of individuals in the gallery (GLOBAL = 275, the test datasets). PCs, principal components; BMI, body mass index.

Similar to the EURO cohort, these results indicate that the use of selected genomic PCs associated to facial shape is as good as using all genomic PCs available. Compared to the results reported in Table 2 of the main manuscript, the performances are along the same line, but lower than using face-to-DNA classifiers.

Supplementary Note 2: Supplementary analysis on genomic PCs in the EURO cohort

In our work, the investigation of genomic PCs in the EURO cohort was mainly restricted to those selected following a GWAS paradigm to control for population stratification. Therefore, we mainly investigated the first four genomic PCs, of which only the first and the fourth showed a good association to facial shape. Here, we investigated the contribution of additional genomic PCs in the EURO cohort, each time using the genomic PCs only and using the genomic PCs augmented with the 32 SNPs to see if the contribution of the SNPs is lost when adding more genomic PC based face-to-DNA classifiers. Additional genomic PCs were selected by 1) looking beyond the first 4 genomic PCs, and 2) lowering the selection threshold from $5x10^{-5}$ to $5x10^{-4}$, $5x10^{-3}$, and $5x10^{-2}$. The biometric performances are listed in Supplementary Table 7.

Genomic PCs	Threshold	Nr	EER	σ	AUC	σ	R1 (%)	σ	R10 (%)	σ	R20 (%)	σ
1 to 4	5e-05	2	0.427	0.003	0.607	0.005	1.354	0.003	17.046	2.526	31.153	1.407
1 to 1000	5e-05	5	0.427	0.008	0.615	0.005	1.580	0.706	17.157	1.582	32.844	0.622
1 to 1000	5e-04	5	0.427	0.009	0.615	0.005	1.580	0.706	17.271	1.721	32.731	0.471
1 to 1000	5e-03	204	0.445	0.018	0.577	0.014	2.032	0.590	14.785	1.188	27.537	2.878
1 to 1000	5e-02	204	0.446	0.028	0.577	0.014	2.032	0.590	14.446	2.400	27.537	2.333
Genomic PCs + SNPs												
1 to 4	5e-05	2	0.375	0.010	0.671	0.008	2.709	0.898	25.623	3.350	40.972	1.802
1 to 1000	5e-05	5	0.373	0.015	0.673	0.008	2.935	0.707	25.848	2.017	41.875	1.097
1 to 1000	5e-04	5	0.374	0.014	0.673	0.008	2.935	0.707	25.848	2.017	41.762	1.257
1 to 1000	5e-03	204	0.395	0.004	0.645	0.008	3.386	0.344	22.686	0.301	36.457	1.947
1 to 1000	5e-02	204	0.395	0.004	0.644	0.008	3.048	0.682	22.460	1.256	36.682	1.699

Supplementary Table 7: Average identification and verification results over the three test runs for different selections of genomic PCs with or without SNPs in the EURO cohort. Genomic PCs, the amount of genomic PCs investigated; Threshold, the threshold applied to select genomic PCs; Nr, the amount of genomic PCs selected; EER, verification equal error rate; AUC, verification area under the curve; R1, rank 1% identification rate; R10 rank 10% identification rate; R20 rank 20% identification rate; σ standard deviation. Random performance is given as EER=0.5, AUC=0.5, R1=1%, R10 = 10%, R20 = 20%. % refers to the percentage of individuals in the gallery (EURO = 275, the test datasets). PCs, principal components; SNPs, single nucleotide polymorphisms

Three main observations are made from these results. First, at the threshold of $5x10^{-5}$ (as applied in the manuscript) the results are close to the same when investigating the first four genomic PCs only. Second, the incorporation of additional genomic PCs, by lowering the selection threshold decreases the performance notably. This further confirms that sufficient statistical association of molecular features to facial shape is required in the proposed paradigm. Third, in each scenario of different genomic PCs, the added SNPs improve the performances consistently.

Supplementary Note 3: Simulated DNA-inferred age and BMI

Age estimation from DNA methylation markers is of fundamental relevance in forensics and has witnessed an exponential spread of interest. In order to simulate DNA predicted age, we refer to the paper of Xu et al.[3] as they achieved the least mean absolute deviation value (MAD) for age prediction from DNA methylation markers. They reported a MAD = 2.8 year from real chronological age. In order to reach this value, we computed the MAD value between self-reported age and age corrupted by increasing noise levels until it reached the value reported in Xu et al.

Body mass index (BMI) is a non-invasive and inexpensive tissue mass measure in an individual. It is an important risk indicator for obesity and related diseases and most importantly, its prediction from the genotype leads to new insights compared to the measured BMI. Guo et al.[4] reported a prediction standard error between genetically predicted versus measured BMI of 0.0266. We added this value to measured BMI in order to simulate genetically predicted BMI from the self-reported height and weight values.

Subsequently, we used simulated DNA predicted age and BMI values from noise injected self-reported values, reaching the prediction accuracies reported in the literature, prior to grouping into upper/lower classes. Recognition performances for age and BMI under various levels of noise injection are presented in Supplementary Figure 13, where we observed only small declines in performance using the reported levels of DNA-based prediction accuracies. This was mainly due to the fact that we turned these continuous variables into a "cruder" two-class classification, where deviations on predicted values only affected a small portion of the individuals at the boundary between both classes.

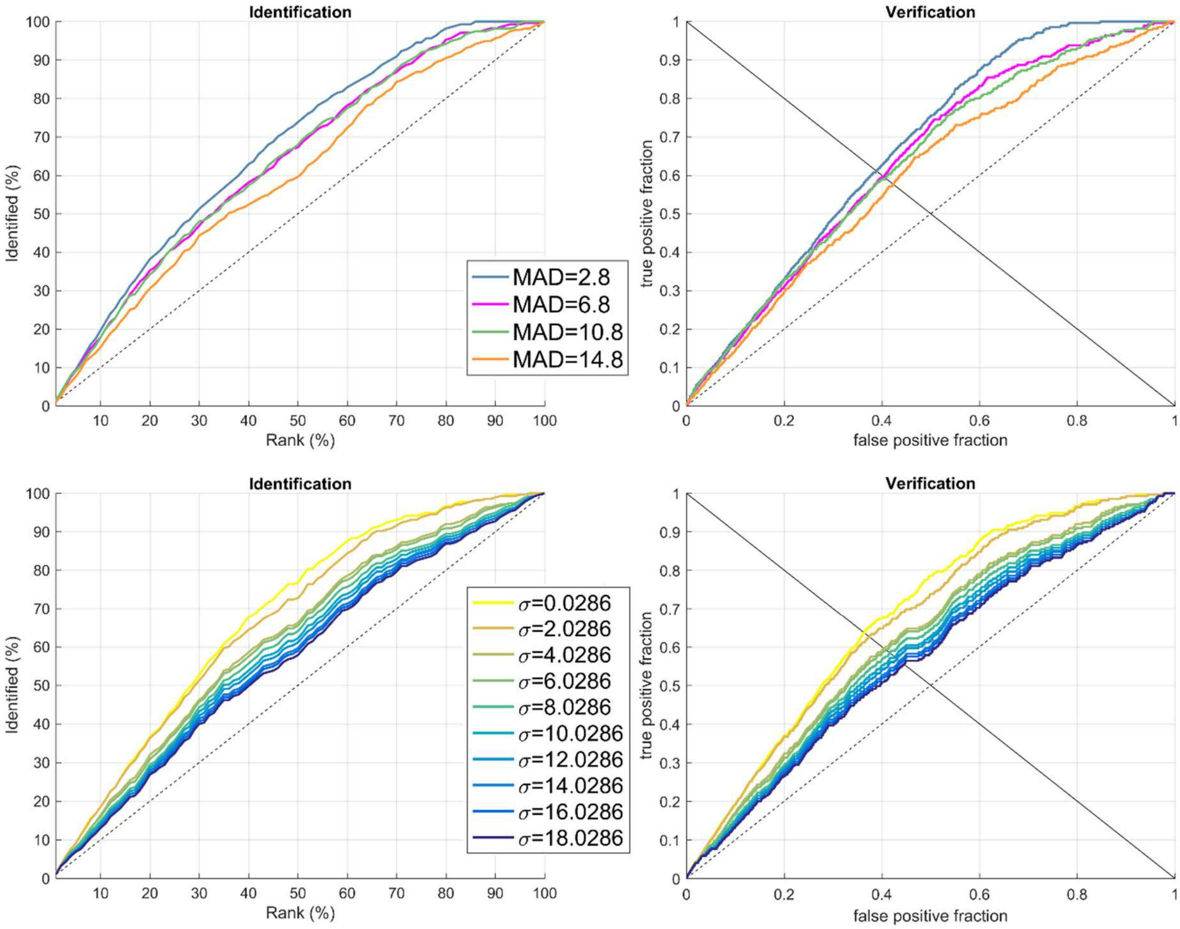

Supplementary Figure 13. Simulated effects on age and BMI. Self-reported age and BMI values were injected with increasing level of noise, starting from the prediction accuracies from DNA as found in the literature (MAD = 2.8 years for age and standard deviation (sigma) = 0.0286 for BMI). The results refer to one single data fold. For age (top), noise was added to self-reported age in the form of a standard normal distribution which was multiplied to a value within the interval [3.5 20] with a step = 5. For BMI (bottom), we multiplied a standard deviation (range = [0.0286 20], step = 2) to a standard random distribution and added it to BMI. For age, we observe a decline in performance when MAD = 6.8 and particularly accentuated for MAD = 14.8, where the curves also lose their desired rounded up shapes (instead clear dents and drop backs in the curves are seen). For BMI, we observe a decline in performance approaching chance results. Similar results were obtained on the EURO cohort (data not shown). BMI, body mass index; MAD, median absolute distance.

Supplementary Note 4: Forensic and criminal justice challenges

Methods like DNA facial phenotyping and matching faces directly to DNA raise undeniable risks of racial disparities in criminal justice that warrant caution against premature application of the techniques until proper safeguards are in place. Public debates on pending legislation would be enriched if they were to include consideration of explicit and implicit biases of those individuals involved[5] and algorithmic biases[6] in the computational techniques and training panel study designs. Understanding the biases of the fragmented databases used by local, state, and federal authorities is necessary to inform the development of pragmatic policies for how law enforcement could use such techniques responsibly in ways that would not infringe upon rights of innocent individuals under the US constitution or erode the public's trust.

Georgetown Law's Center on Privacy & Technology has devoted efforts to better understanding the use of face-to-face recognition technologies in the United States and has developed important online resources[7] to guide discussions regarding the ethical, legal, and social implications (ELSI), which are also relevant to the proof of concept face-from DNA recognition research reported here, including a review of law enforcement's use of facial recognition technologies by jurisdiction, model face recognition legislation, and a model face recognition use policy[7]. The Center issued 30 specific recommendations for legislatures, law enforcement, industry, and community leaders to establish responsible limits on face recognition. Among the recommendations are that Congress and state legislatures should pass measures that would require mug shot databases used for face recognition to exclude photos of those individuals who have been found not-guilty or against whom charges were dropped or dismissed, prohibit the use of driver's license databases for face recognition unless expressly allowed by state statutes, promote more diverse photo datasets for training, and condition state and federal financial assistance on transparency, oversight, and accountability[7].

In its 2009 report Strengthening Forensic Science in the United States: A Path Forward, the National Research Council detailed the many challenges facing forensic sciences and strongly advocated for the establishment of the National Institute of Forensic Science, which has not yet happened. In 2013 the National Commission on Forensic Science (NCFS) was created as a Federal Advisory Committee [https://www.gsa.gov/portal/content/100916] to "enhance the practice and improve the reliability of forensic science" and make policy recommendations to the U.S. Attorney General. The NCFS had notable breadth of expertise among its members, and the NCFS provided a much-needed independent forum where forensic science could be critiqued and improved and where policies could be developed for the appropriate use of forensic science by law enforcement and in courtroom settings. Unfortunately, the Trump Administration allowed the NCFS to expire on April 23, 2017. As summarized in its final report, the NCFS adopted 43 work products (ten of which focused on foundational issues to ensure the validity, accuracy, and effectiveness of forensic science) but considered its mission of "determining how to move forward in creating a more robust research culture" to be unfinished [https://www.justice.gov/archives/ncfs/page/file/959356/download]. This was met with outspoken criticism—including, for example, by U.S. Senators Richard Blumenthal and Cory A. Booker, in a letter submitted on June 9, 2017, responded to the Department of Justice's request for public comment [Docket No. OLP 160] by stating "We believe allowing this federal advisory committee to expire was a mistake, and that there is a very easy and simple answer as to how the Department ought to proceed; the Department ought to renew the Commission's charter" [regulations.gov tracking number 1k1-8wv9-qm9h]. John F. Holloway, Executive Director of the Quattrone Center

for the Fair Administration of Justice at the University of Pennsylvania Law School, stated the decision "creates a substantial void and reduces the quality of forensic science" and emphasizing the need for "independence, fairness, and transparency" in "Response of the Quattrone Center for the Fair Administration of Justice at the University of Pennsylvania Law School to the Department of Justice Notice of Public Comment Period on Advancing Forensic Science, Docket No. OLP 160," submitted June 8, 2017 [regulations.gov tracking number 1k1-8wug-s31c]. The American Association for the Advancement of Science, American Chemical Society, Federation of Associations in Behavioral and Brain Sciences, and Human Factors and Ergonomics Society letter in response to DOJ-LA-2017-0006-0001, submitted June 9, 2017, explained that the NCFS "has served a crucial role in bringing together all relevant stakeholders" and attributing its ability "to make progress on multiple fronts" to this broad stakeholder engagement [regulations.gov tracking number 1k1-8wv6-rc9c]. The Department of Justice later announced it would launch a Forensic Science Working Group; however, it is unclear whether this internal group will promote more reliable forensic science or policies for fairness in their practice and application [See, e.g., Alan Pyke. "Sessions relaunches Obama-era forensics review months after he shuttered it." Think Progress. August 8, 2017.]. The Innocence Project, for example, publicly expressed disappointment in this move "away from a public, transparent, and science-centered process..." [Pema Levy. "Sessions' New Forensic Science Advisor Has a History of Opposing Pro-Science Reforms." Mother Jones. August 10, 2017]. The proof of concept reported here for facial recognition from DNA underscores the need for rigorous scientific critique and transparent policy deliberations to ensure that any practice or application by law enforcement be delayed until there are robust foundational data for its validity and reliability, adequate operational safeguards in place for quality assurance, and sufficient guidance for communication and translation to achieve fairness.

Supplementary Note 5: Re-identification and privacy challenges in genomic research

The proof of concept reported here for facial recognition from DNA also underscores the critical and increasing importance of ethical, legal, and social implications (ELSI)-research related to the privacy challenges in genomic research. The National Human Genome Research Institute, for example, has articulated a diverse set of prioritized research domains, which include topics relevant to this proof of concept method, including (but not limited to) re-identification, security, and data privacy topics[8]. Before this proof of concept approach is implemented in any setting (forensic or otherwise), it should be preceded by a multifaceted ELSI risk assessment. The following is offered as an introduction to some of the pertinent issues that might serve as a starting point for relevant literature review and further scholarly inquiry.

Open access has served the genomics and molecular genetics fields well with rapid and relatively easy access to raw data and policies that include releasing data prior to publication. When genomic data on living persons is connected to sensitive data (such as data from health records, financial records, consumer transactions, education, employment, housing, and other societal contexts), the release of these data might challenge the participant's privacy and confidentiality. One solution that has been widely supported and implemented in human participants' data sharing protocols is to de-identify the participant data, separating the personally identifiable information (e.g., name, phone number, medical record number) from the sensitive and genotype data. However, in the context of this work, it is necessary to acknowledge that the identifiability of human genomes and reasonable expectations of genomic privacy have been the focus of extensive ELSI scholarship[9–12]. The importance of this privacy issue is boosted by the wide availability of facial images and widespread use of these images by companies who are all marketing facial applications (namely, Google, Amazon, IBM, Microsoft, and Facebook)—which, not coincidentally, have recently come under fierce criticism for the exploitation of those pictured, insufficient consent processes, and biases (racial, ethnic, and gender) [See, e.g., IBM using Flickr images[13–15] and Amazon's facial recognition doorbell plans[16,17]].

The ELSI community increasingly appreciates that DNA is "uniquely identifiable"[18] and "the ultimate digital identifier"[19] and that "de-identification" of genomes is a delusion [JK Wagner. "Re-Identification Is Not the Problem. The Delusion of De-Identification Is." Harvard Law Petrie-Flom Center Bill of Health online symposium on the Law, Ethics, and Science of Re-Identification Demonstrations, organized by Michelle Meyer. May 22, 2013]. Rodriguez et al.[20] (2013) acknowledged recent studies have "call[ed] into question whether the goal of complete de-identification of many types of human data is realistic in today's information-rich society". Some have suggested that governance efforts might be better spent focusing attention less on mitigation of risks and more on mitigation of actual harms[21], and several "privacy-preserving strategies" have been suggested, including minimizing risks of re-identification of individuals who have participated in genomic research through various forms of data access controls, data anonymization (using, for example, k-anonymity or differential privacy techniques), and cryptographic solutions[22]. Given the challenges of identifiability and the "rise and fall of de-identification,"[23] several scholars have been firm advocates for open consent practices[24] that emphasize veracity of consent through candid, honest disclosure of the risks of participation and ending the practice of making "potentially disingenuous promises of anonymity, privacy, and confidentiality."[25] Some have advocated a shift in attention from balancing data privacy and utility to enabling trust[23] or promoting solidarity[21]. Support for an open approach is not universal, with some warning of the negative consequences of a surveillance state and the challenges of an

informed consent approach for genomic research that remains focused on an individual, which fails to account for the probabilistic information that can be gleaned—and societal risks that accompany those insights—regarding unaware relatives or community members (see, e.g., Vayena et al.[26], Pereira et al.[27], Ram et al.[28,29], Clayton et al.[11], Wang et al.[9,30], Bloss et al.[31], Greenbaum et al.[32], Goodman et al.[33], Lemke et al.[34], Prictor et al.[35], Borry et al.[36], Fisher and Layman[37], Gabel Cino[38], Carrero et al.[39]). Again, others might advocate against expanding open access to and circulation of genetic information. No consensus solution to these and other complex issues has yet arisen from ELSI-research on policymakers.

Legal scholars have written extensively on some of the constitutional concerns related to increasing law enforcement use of DNA, facial recognition, and other emerging technologies (including the general use of Big Data, machine learning, and artificial intelligence) [See, e.g., Koops and Schellekens[40], Maclean[41], Wagner[42], Gabel Cino[38,43], Gusella[44], Hodge[45], Hirose[46], Pearlman and Lee[47], Nakar and Greenbaum[48], Simmons[49], Joh[50], Berman[51], Ferguson[52], Brown[53], Kohne[54], Reamay[55], Monajemi[56], Pope[57], Carrero[39], Cuador[58], Sklansky[59], Murphy[60], Kaye[61], Dedrickson[62], Ram[29,63], Guest[64], Logan[65], Strutin[66], Garrett[67], Ferguson[52,68], Froomkin[69]]. Biometric identifiers have been described as "one of the most unprotected areas of our personal identity"[57], and scholars have lamented the many ways in which the public is being "desensitized"[56] to "privacy-sacrificing technologies"[70] or "privacy piercing technology."[71]. Some[50] have underscored the importance in recognizing the public's acts of resistance to governmental surveillance in order to make sense of privacy in modern society. While some[52] argue that a "big data-infused reasonable suspicion standard" is possible, others[55] urge us to abandon a quest for a bright-line rule when setting the boundaries for governmental searches and seizures involving specific technological tools and instead focus on core principles of the Fourth Amendment as an "expression of shared values" that can be ascertained by courts using empiricism and social science to determine what those shared values are. Yet other scholars[72], with regard to facial recognition technology, have focused on a distinction between the right to be seen in public and the right to be recognized. Particularly relevant to this proof of concept, if it were to be applied by law enforcement, is the concern that some legal scholars have voiced regarding the need for oversight because privacy concerns will actually increase as the technology's accuracy improves[43]. Scholars have been divided[60–62] about whether universal databases could be preferable and even increase privacy[62] relative to known, current approaches. One[66] has even remarked that "the registry of human blueprints will be the never-ending battleground of privacy."

Efforts to strengthen privacy assurances continue, including shields to resist Freedom of Information Act (FOIA) requests for research data and NIH Certificates of Confidentiality as shields provided to all federally-funded researchers to resist compelled disclosures of research data in legal proceedings, both of which became law as part of the 21st Century Cures Act [21st Century Cures Act, Pub. L. No. 114-255 (2016)]. Notably, however, identifiability continues to lack consistent definition in federal policy or practice and remains an area in desperate need of harmonization[73]. Furthermore, medical biobanks and forensic databases continue to be treated distinctly in ELSI scholarship[74], despite early recognition that erosions of public trust in genomics in one social arena will influence trust in others and calls for ELSI researchers to anticipate also more on non-medical applications (such as forensics) of genome sciences and technologies[75]. Much ELSI research on privacy challenges in genomic research is focused on medical applications, which is also reflected by much of the citations using throughout this Supplementary Note. With regard to forensic contexts, there have been calls to protect privacy as well as enhance oversight of crime labs[67]. One particular target of concern has been the potential exemption of

forensic databases from the Privacy Act of 1974 (such as the concerns legal scholars have raised regarding the FBI's Next Generation Identification System), which would make it difficult not only to know if a specific individual's data is contained therein but also to control the agencies and parties with whom the data are shared without consent[39,57].

Forensically, in 2018 the use of non-law enforcement databases (namely, recreational genealogy platforms wherein users may upload their direct-to-consumer genomic profiles and compare their profiles with those of other users to identify potential genetic relatives) by law enforcement to solve cold cases also prompted renewed discussions about the adequacy of current privacy protections in the United States and questions regarding whether the sector-specific approach should be replaced with a uniform data privacy approach more similar to the European Union's General Data Protection Regulation 2016/679 (GDPR) or China's Cybersecurity Law and Information Security Technology – Personal Information Security Specification. While no consensus of opinions has yet emerged, the Golden Serial Killer, East Bay Rapist, and Visalia Ransacker investigation (that relied upon use of GEDmatch comparisons to generate new leads to be followed with traditional investigation techniques and resulted, ultimately, in an arrest) has sparked considerable discussion in the public, academia, and forensic community. Some have proposed legislative or regulatory solutions[63] while others have proposed technological solutions[76] to prevent over-reliance or abuse of these resources. Meanwhile, companies have begun dedicating services to this endeavor, and the number of cold cases investigations able to be solved in this way continues to grow[77,78]. Researchers will need to follow policy developments with these applications closely as well as the development of state-specific legislation on biometric data protections[54] [see also state statutes such as, e.g., California Consumer Privacy Act of 2018; Vermont's An Act Relating to Data Brokers and Consumer Protection, H.764, 2018 Sess. (VT 2018), Act 171 of 2018, 9 VSA §§ 2430, 2433, 2446 and 2447 (May 22, 2018); Colorado Act Concerning Strengthening Protections for Consumer Data Privacy HB18-1128 (May 29, 2018); Ohio's Data Protection Act, SB 220, Ohio Rev. Code §1354.01 et seq.; Illinois' Biometric Information Privacy Act (or BIPA), codified as 740 ILCS/14 and Public Act 095-994 (October 3, 2008)] in order to be able to inform prospective research participants adequately about risks and benefits of genomic research and potential uses of the information.

Supplementary Information References

1. Gibbs, R. A. *et al.* The International HapMap Project. *Nature* **426**, 789–796 (2003).
2. Lippert, C. *et al.* Identification of individuals by trait prediction using whole-genome sequencing data. *Proc. Natl. Acad. Sci.* **114**, 10166–10171 (2017).
3. Xu, C. *et al.* A novel strategy for forensic age prediction by DNA methylation and support vector regression model. *Sci. Rep.* **5**, 17788 (2016).
4. Guo, Y. *et al.* Genetically Predicted Body Mass Index and Breast Cancer Risk: Mendelian Randomization Analyses of Data from 145,000 Women of European Descent. *PLOS Med.* **13**, e1002105 (2016).
5. Atiba Goff, P. & Barsamian Kahn, K. Racial Bias in Policing: Why We Know Less Than We Should. *Soc. Issues Policy Rev.* **6**, 177–210 (2012).
6. Caliskan, A., Bryson, J. J. & Narayanan, A. Semantics derived automatically from language corpora contain human-like biases. *Science (80-.).* **356**, 183–186 (2017).
7. Garvie, C. Perpetual Line-Up: Unregulated Police Face Recognition in America. (2016). Available at: https://www.perpetuallineup.org/.
8. ELSI Research Domains - National Human Genome Research Institute (NHGRI). (2017). Available at: https://www.genome.gov/27543732/elsi-research-domains/.
9. Wang, S. *et al.* Genome privacy: challenges, technical approaches to mitigate risk, and ethical considerations in the United States. *Ann. N. Y. Acad. Sci.* **1387**, 73–83 (2017).
10. Shi, X. & Wu, X. An overview of human genetic privacy. *Ann. N. Y. Acad. Sci.* **1387**, 61–72 (2017).
11. Clayton, E. W., Halverson, C. M., Sathe, N. A. & Malin, B. A. A systematic literature review of individuals' perspectives on privacy and genetic information in the United States. *PLoS One* **13**, e0204417 (2018).
12. Majumder, M. A., Cook-Deegan, R. & McGuire, A. L. Beyond Our Borders? Public Resistance to Global Genomic Data Sharing. *PLOS Biol.* **14**, e2000206 (2016).
13. IBM used Flickr photos for facial-recognition project. (2019). Available at: https://www.bbc.com/news/technology-47555216.
14. Dans, E. The Day I Fed My Friends To An IBM Algorithm. (2019). Available at: https://www.forbes.com/sites/enriquedans/2019/03/13/the-day-i-fed-my-friends-to-an-ibm-algorithm/#647162de7233.
15. Jee, C. People's online photos are being used without consent to train face recognition AI - MIT Technology Review. (2019). Available at: https://www.technologyreview.com/the-download/613118/peoples-online-photos-are-being-used-without-consent-to-train-face-recognition/.
16. Snow, J. Amazon's Disturbing Plan to Add Face Surveillance to Your Front Door. (2019). Available at: https://www.aclu.org/blog/privacy-technology/surveillance-technologies/amazons-disturbing-plan-add-face-surveillance-yo-0.
17. Doffman, Z. Amazon Refuses To Quit Selling 'Flawed' And 'Racially Biased' Facial

Recognition. (2019). Available at: https://www.forbes.com/sites/zakdoffman/2019/01/28/amazon-hits-out-at-attackers-and-claims-were-not-racist/#7a92a92846e7. (Accessed: 10th April 2019)

18. McGuire, A. L. Identifiability of DNA data: the need for consistent federal policy. *Am. J. Bioeth.* **8**, 75–6 (2008).
19. Angrist, M. Eyes wide open: the personal genome project, citizen science and veracity in informed consent. *Per. Med.* **6**, 691–699 (2009).
20. Rodriguez, L. L., Brooks, L. D., Greenberg, J. H. & Green, E. D. The Complexities of Genomic Identifiability. *Science (80-.).* **339**, 275–276 (2013).
21. Prainsack, B. & Buyx, A. A solidarity-based approach to the governance of research biobanks. *Med. Law Rev.* **21**, 71–91 (2013).
22. Erlich, Y. & Narayanan, A. Routes for breaching and protecting genetic privacy. *Nat. Rev. Genet.* **15**, 409–421 (2014).
23. Erlich, Y. *et al.* Redefining Genomic Privacy: Trust and Empowerment. *PLoS Biol.* **12**, e1001983 (2014).
24. Lunshof, J. E., Chadwick, R., Vorhaus, D. B. & Church, G. M. From genetic privacy to open consent. *Nat. Rev. Genet.* **9**, 406–411 (2008).
25. Lunshof, J. E. *et al.* Personal genomes in progress: from the human genome project to the personal genome project. *Dialogues Clin. Neurosci.* **12**, 47–60 (2010).
26. Vayena, E. & Gasser, U. Between Openness and Privacy in Genomics. *PLOS Med.* **13**, e1001937 (2016).
27. Pereira, S., Gibbs, R. A. & McGuire, A. L. Open access data sharing in genomic research. *Genes (Basel).* **5**, 739–47 (2014).
28. Ram Natalie. DNA by the Entirety. *Columbia Law Rev.* **115**, 874–938
29. Ram Natalie. Incidental Informants Police Can Use Genealogy Databases to Help Identify Criminal Relatives-but Should They? *Md. B.J.* 8–9 (2018).
30. Wang, S. *et al.* A community effort to protect genomic data sharing, collaboration and outsourcing. *NPJ Genomic Med.* **2**, 33 (2017).
31. Bloss, C. S. Does family always matter? Public genomes and their effect on relatives. *Genome Med.* **5**, 1–3 (2013).
32. Greenbaum, D., Sboner, A., Mu, X. J. & Gerstein, M. Genomics and Privacy: Implications of the New Reality of Closed Data for the Field. *PLoS Comput. Biol.* **7**, e1002278 (2011).
33. Goodman, D. *et al.* A comparison of views regarding the use of de-identified data. *Transl. Behav. Med.* **8**, 113–118 (2018).
34. Lemke, A. A., Smith, M. E., Wolf, W. A., Trinidad, S. B. & GRRIP Consortium. Broad data sharing in genetic research: views of institutional review board professionals. *IRB* **33**, 1–5 (2011).
35. Prictor, M., Teare, H. J. A. & Kaye, J. Equitable Participation in Biobanks: The Risks and Benefits of a "Dynamic Consent" Approach. *Front. Public Heal.* **6**, 253 (2018).

36. Borry, P. *et al.* The challenges of the expanded availability of genomic information: an agenda-setting paper. *J. Community Genet.* **9**, 103–116 (2018).

37. Fisher, C. B. & Layman, D. M. Genomics, Big Data, and Broad Consent: a New Ethics Frontier for Prevention Science. *Prev. Sci.* **19**, 871–879 (2018).

38. Gabel Cino, J. Tackling Technical Debt: Managing Advances in DNA Technology that Outpace the Evolution of Law. *J. Civ. Leg. Sci.* **54**, 420–21 (2016).

39. Carrero, A. Biometrics and federal databases: could you be in it? *John Marshall Law Rev.* 1–21 (2019).

40. Koops, B.-J. & Schellekens, M. H. M. Forensic DNA Phenotyping: Regulatory Issues. *Ssrn* 1–38 (2007). doi:10.2139/ssrn.975032

41. Maclean, C. E. Creating a Wanted Poster from a Drop of Blood: Using DNA Phenotyping to Generate an Artist' s Rendering of an Offender Based Only on DNA Shed at the Crime Scene Part of the Civil Rights and Discrimination Commons, and the Criminal Law Commons Recommended. *Hamline Law Rev.* **36**, 1–26 (2014).

42. Wagner, J. K. & Brennan, J. Dna , Racial Disparities , and Biases in Criminal Justice : Searching for Solutions. *Albany Law J. Sci. Technol.* **27**, 95–138 (2017).

43. Cino, J. G. Deploying the Secret Police: the Use of Algorithms in the Criminal Justice System. *Ga. St. U. L. Rev.* **34**, 1093–94 and 1101 (2018).

44. Gusella, D. No Cilia Left Behind: Analyzing the Privacy Rights in Routinely Shed DNA Found at Crime Scenes. *L. Rev* **54**, 789 (2013).

45. Hodge, S. Current Controversies in the Use of DNA in Forensic Investigations. *Univ. Balt. Law Rev.* **48**, 65–66 (2018).

46. Hirose, M. Privacy in public spaces: the reasonable expectation of privacy against the dragnet use of facial recognition technology. *Conn. L. Rev.* **49**, (2017).

47. Pearlman, A. R. & Lee, E. S. National Security, Narcissism, Voyeurism, and Kyllo: How Intelligence Programs and Social Norms Are Affecting the Fourth Amendment. *Texas A&M Law Rev.* **2**, (2014).

48. Greenbaum, D. & Nakar, S. Now You See Me: Now You Still Do: Facial Recognition Technology and the Growing Lack of Privacy. *Bost. Univ. J. Sci. Technol. Law* **23**, 88–122 (2017).

49. Simmons, R. Quantifying Criminal Procedure: How to Unlock the Potential of Big Data in Our Criminal Justice System. *Ssrn* 1–51 (2016). doi:10.2139/ssrn.2816006

50. Joh, E. E. Privacy Protests: Surveillance Evasion and Fourth Amendment Suspicion. *Ariz. L. Rev.* **55**, 997–1029 (2013).

51. Berman, E. A government of laws and not machines. *B.U. L. Rev.* **98**, 1277–1355 (2018).

52. Ferguson, A. G. Big Data and Predictive Reasonable Suspicion. *U. Pa. L. Rev.* **163**, 327–336 (2015).

53. Brown, K. N. Anonymity, Faceprints, and the Constitution. *Geo. Mason L. Rev.* **21**, 409–466 (2014).

54. Kohne, N. & Salour, K. Biometric Privacy Litigation: Is Unique Personally Identifying Information Obtained from a Photograph Biometric Information? *Compet. J. Anti., UCL Priv. Sec. St. B. Cal.* **25**, (2016).
55. Reamey, G. S. Constitutional Shapeshifting: Giving the Fourth Amendment Substance in the Technology Driven World of Criminal Investigation. *Stanford J. Civ. Rights Civ. Lib.* **14**, 201–245 (2018).
56. Monajemi, M. Privacy Regulation in the Age of Biometrics That Deal With a New World Order of Information. *U. Miami Int'l Comp. L. Rev.* **25**, 407–08 (2018).
57. Pope, C. Biometric Data Collection in an Unprotected World: Exploring the Need for Federal Legislation Protecting Biometric Data. *J.L. Pol'y* **26**, 769, 770 (2018).
58. Cuador, C. From Street Photography To Face Recognition: Distinguishing Between The Right To Be Seen And The Right To Be Recognized. *Nova Law Rev.* **41**, (2017).
59. Sklansky, D. A. Two More Ways Not to Think about Privacy and the Fourth Amendment. *Univ. Chicago Law Rev.* **82**, 223–242 (2015).
60. Murphy, E. Relative Doubt: Familial Searches of DNA Databases. *Mich. Law Rev.* **109**, 329–30 (2010).
61. Kaye, D. H. & Smith, M. E. DNA identification databases: Legality, legitimacy, and the case for population-wide coverage. *Winsconsin Law Rev.* **3**, 414–459 (2003).
62. Dedrickson, K. Universal DNA databases: a way to improve privacy? *J. Law Biosci.* **4**, 637–647 (2017).
63. Ram, N., Guerrini, C. J. & McGuire, A. L. Genealogy databases and the future of criminal investigation. *Science* **360**, 1078–1079 (2018).
64. Guest Christine. DNA and Law Enforcement: How the Use of Open Source DNA Databases Violates Privacy Rights. *Am. Univ. Law Rev.* **68**, (2019).
65. Logan, W. A. Policing Police Access to Criminal Justice Data. *Iowa L. Rev.* **104**, (2019).
66. Strutin, K. DNA Without Warrant: Decoding Privacy, Probable Cause and Personhood. *Rich. J.L. Pub. Int.* **18**, 319–366 (2015).
67. Garrett, B. L. The Crime Lab in the Age of the Genetic Panopticon (Book Review). (2017).
68. Ferguson, A. G. Personal Curtilage: Fourth Amendment Security in Public. *Wm. Mary L. Rev.* **55**, 1283–1284 (2014).
69. Froomkin, A. M. Regulating Mass Surveillance as Privacy Pollution: Learning from Environmental Impact Statements. *U. Ill. L. Rev.* 1713–1716 (2015).
70. Pearlman, A. & Lee, E. National Security, Narcissism, Voyeurism, and Kyllo: How Intelligence Programs and Social Norms are Affecting the Fourth Amendment. *Tex. A&M L. Rev.* **2**, 776–778 (2015).
71. Nesbitt Cosby, T. The Expectation of Privacy: An Unreasonable Standard in an Era of Rapid Innovations in Technology. *Charlest. L. Rev.* **12**, (2018).
72. Cuador, C. From Street Photography to Face Recognition: Distinguishing Between the

Right to Be Seen and the Right to Be Recognized. *Nov. L. Rev.* **41**, 237–264 (2017).

73. Majumder, M. A., Guerrini, C. J., Bollinger, J. M., Cook-Deegan, R. & McGuire, A. L. Beyond Our Borders? Public Resistance to Global Genomic Data Sharing. *Genet. Med.* **19**, 1289–1294 (2017).

74. Machado, H. & Silva, S. Public participation in genetic databases: crossing the boundaries between biobanks and forensic DNA databases through the principle of solidarity. *J. Med. Ethics* **41**, 820–824 (2015).

75. Cho, M. K. & Sankar, P. Forensic genetics and ethical, legal and social implications beyond the clinic. *Nat. Genet.* **36**, S8–S12 (2004).

76. Erlich, Y., Shor, T., Carmi, S. & Pe'er, I. Re-identification of genomic data using long range familial searches. *bioRxiv* 350231 (2018). doi:10.1101/350231

77. 29th International Symposium on Human Identification, September 24-27, 2018. In: Phoenix, AZ.

78. Brooks A. (presenter) A DNA Database Helped Find A Suspected Serial Killer. How Is Your Privacy Affected? [Podcast], May 03, 2018.
