# Supplementary material for: Facial recognition from DNA using face-to-DNA classifiers
Source: Nat Commun. 2019 Jun 11;10:2557. doi: 10.1038/s41467-019-10617-y (PMC6560034; doi:10.1038/s41467-019-10617-y)
Supplement: Supplementary file 4 — Description of Additional Supplementary Files [file 41467_2019_10617_MOESM4_ESM.docx]

**Description of Additional Supplementary Files**

**File Name: Supplementary Data 1**

**Description:** Statistical facial associations to genomic principal components. Excel file with results on the statistical canonical correlation analyses of genomic principal components onto facial shape for both the EURO and the GLOBAL cohort.

**File Name: Supplementary Data 2**

**Description:** Results on alternative classifiers and parameter tuning. Excel file with additional results on the GLOBAL cohort using a range of alternative classifiers to support vector machines that are implemented and readably available in MatlabTM as well as different strategies in MatlabTM to set the hyper-parameters in support vector machines.
