# Supplementary material for: Facial recognition from DNA using face-to-DNA classifiers
Source: Nat Commun. 2019 Jun 11;10:2557. doi: 10.1038/s41467-019-10617-y (PMC6560034; doi:10.1038/s41467-019-10617-y)
Supplement: Supplementary file 3 — Peer Review File [file 41467_2019_10617_MOESM3_ESM.pdf]

Reviewers' comments:

Reviewer #1 (Remarks to the Author):

For the paper ‘Facial Recognition from DNA’, the authors describe a new methodology in the prediction of an unknown individuals face from DNA. The authors describe a very interesting alternative to phenotypic prediction from genotypic information, rather than describing a face entirely from DNA, the authors propose to use the backing of established biometrics to ‘compare’ a face to a set of faces (using only trained genetic identifiers) to see if a match is a) present in a group of faces, b) assign a strength of match to one face. Although there is merit to this approach and the amount of data in this paper is quite substantial, this reviewer feels that some of the data detracts from the overall message, i.e. there is too much going on while reading the paper that clouds the overall approach, which is the whole point of the article. In some instances, there is also not enough genetic data to back up some of the overloaded points in this paper, therefore the reviewer feels that if the paper was more structured and not as convoluted, it would get the message across much better. Below are suggested improvements for restructuring of the paper to reach the standard of publication in this journal as in its current state, it is not acceptable.

Abstract:

I think the overall abstract is an overstatement of the methodology when comparing to the prediction of unknown face from DNA, as they are different approaches. Even with this suggestive approach of using machine learning to train how segments of the face look with regards sex, genomic background and SNPs, one must have access to a group of faces to compare the unknown DNA to, if not, then this methodology will not work, this obvious drawback is missing from here. If one had a group of faces to compare to, regardless of methodology, it is obviously easier to ‘match’ a person.

Introduction:

The author’s suggestion of reframing the problem is commendable but perhaps rephrasing it would be better. Currently it says ‘instead of predicting an ungiven face from DNA, is it possible to match a given face to DNA using face-to-DNA mapping’ these are two different questions, it should read…instead of predicting an ungiven face from unknown DNA, is it possible to match unknown DNA to a given face using face-DNA mapping’, after all it is DNA you have at a crime scene etc. and that is the question on hand – can you get a face, or face match from DNA.

Next, the classification of faces by molecular aspects. For sex, did you actually look at genetic information to classify the DNA was male/female – this is not stated, it looks like you simply

accepted the m/f statement in questionnaire/images and trained from there. Obviously, if you had unknown DNA and a ‘group’ of images to compare to, straight away you can at least remove the opposite sex (from the DNA sample) from you line up list, nothing novel here. However you are suggesting that using certain segments of images on the face, some may be more masculine/feminine, what are the criteria for something more masculine/feminine, do you have data to back these up? You state ‘sex affected the masculinity/femininity of many features across the face, can you elaborate? How does the algorithm ‘see’ these masculine/feminine features, is this something you can better explain/categorize?

The nested approach to facial shape is wonderful and the method has great promise, however the authors seem to be missing the direct genetic connection and rely too much on machine learning (which will be affected by training sets). I understand you are trying to identify related molecular features to use as facial classifiers and that’s fine, but we know sex and genomic background influence the face, this (and you) have already shown this. What is novel here is using the bayes matching approach, if from DNA you know someone is male, they are European (based on the genomic PCs you describe), it is the segments of the face and their association (based on your training) that is more interesting to give a match to your ‘group’ line up of individuals and in essence give a score – that method is novel as it ties in a genetic test to a biometrics comparison/rank score. If you restructure the paper simplifying this, it would make for a much nicer read.

Also your sentence ‘this creates the novel ability to identify (who is this person?)….should be ‘who is this person from this group of people?’, you’re overstating here. I see you state it is a ‘group’ towards the end of the lengthy sentence but please shrink, it’s very convoluted. Remember it is not the same thing, making a face from DNA to give an identity (what you state as a version of DNA phenotyping) is different than identifying someone within a group with their DNA, different questions, not a reframe of the same thing.

The whole section on using two cohorts does not seem beneficial, at least shrink the GLOBAL section – you stated yourself in the discussion that ‘recognition in the GLOBAL cohort is limited to an individuals population background’ so perhaps this should be less lengthy as its been pretty much published before. This approach of using DNA to check the face of a group of people to see if there is a potential match or not, should only be on genetic information or ‘face snps’ you use with sex/background information (however generated) there to dictate which algorithm/training set (use GLOBAL or EURO) to use for the SNP data classifiers to enable the generation of a bayes score in that subset of people. I would suggest the authors streamline their approach based on what they would have, which is the DNA and how they can use their training data to match after they get the raw data from the DNA and not get so caught up in all the different approaches, associations, gwas hits on 100k snp data that may have nothing to do with the facial segment – could be chance etc. (although FDR is in place) that they have put into the paper.

I suggest avoiding too much focus on DNA-inferred classifiers right now (age and BMI) until there is more information and accuracy testing on their reliable prediction from DNA. One would hope that in time it will be a more stable trait to predict, however right now it may be best to cover your face match by correcting for multiple ages/weights instead of trying to build it into the model as a classifier – why – well look at your own passport, that image is not a current photo, so this all comes down to what group of face photos you are comparing your match DNA to. You are certainly missing pigment here and this should certainly be included as an obvious classifier as you have stated in the paper, due to current genetic information and data supporting its prediction from genetic markers.

I am glad to see the authors push for scientifically justified and peer-reviewed processes to be required for facial prediction/matching. Be careful to also include that forensic DNA samples are limited, and where other facial profiling companies produce array data with thousands of SNPs to produce their 'facial renditions' – which is basically sex and ancestry, this is not optimal. Clearly optimizing what can be generated genetically for the DNA probe for each of the classifiers is an important task. The choice of SNPs here will be paramount, as you are correct, it may be science fiction to produce a face from DNA and easier to match a face with known classifiers from DNA as your method proposes, however how do you see this being done, a random array as you have discussed that may not include the correct causal markers or a more specific design that tells the user, which (GLOBAL or EURO) training set to use for your facial matching algorithm (choice of classifiers to use) and score fusing.

It's a pity to see GWAS data lost in the context of this paper due to doing 'too much'. Also the choice of the peak SNP is not ideal, were there any other parameters examined other than lowest p value? Of course it is unlikely these are causal just by chance, and therefore makes this analyses disappointing. Perhaps comparing allele frequency between the snps in these loci would be more beneficial. At least something more than just lowest p value (after >0.5 LD inclusion), as it would drastically affect the association of the alleles (whether dominant or recessive) in the SNP classifier performance with the facial segments – which to me is the most interesting classifier. Even using regulomedb to propose/rank regulatory suspected variants (if the SNP were intronic/intergenic) would be useful rather than randomly choosing the highest associated SNP.

Lastly, when assessing the performance on your test set, what is your marker for sex from the DNA profiles of these individuals? Presence of Y SNPs? Can you explain? It would also be nice if a subset of individuals from the test dataset (both global and euro combined, n=571) were randomly left out, so that you could assess those samples without knowing which dataset they belonged, perhaps a 2-step classification score, one using the 382, the other using the 50 classifiers, for all 571 individuals DNA.

Minor comments:

As you use some 1k genomes for your population structure assessment, would be best to further describe this and reference the set. I could not find this, other than you used it for imputation, and you mention its use in Figure S2 (but didn't reference it).

Overall, the idea of using matching scores based on genetic data quite like biometric matching scores is appealing and therefore this novel idea has merit, however the structure and approach of the paper needs fixing. For example, there is lacking data on how the test data would actually be assessed with regards their DNA and not simply using questionnaire info (as seems to be the case for not only bmi and age, as they are inferred, but also for sex right now). If the whole point of the method is to give values/strength to a match, then that should be the focus of the paper, from DNA input, what and how, to match output, this is stifled by the many other components of the paper.

Reviewer #2 (Remarks to the Author):

The paper presents an approach for identification of individuals from DNA by predictive modeling for multiple phenotypes.

The problem is of importance, both for forensics, as well as in the light of personal privacy of individuals who share anonymized genomic and medical phenotype data for research.

The approach shares similarity to ref. 4, but uses a combination of binary classification instead of a combination of the regression models used in ref4. However, the limited technical details and comparison between the approaches provided in the manuscript make it hard to guess the exact differences and technical contributions of the paper. This lack of clarity and technical detail makes it hard to judge novelty and limits reproducibility.

The main reported finding in an evaluation on two different cohorts, one cohort of diverse ancestry, and a cohort of European ancestry, is that individual SNPs, which have been found significant in a GWAS, contribute to identification performance. However, while this finding would be exciting, in their analysis on the European cohort, the authors only take into account 4 PCs to model tagging of genome-wide genetic variation without showing empirical evidence that additional PCs do not tag phenotypic variation beyond the first 4 PCs.

Such empirical evidence is required, given that it has been found in multiple studies of heritable phenotypes that genome-wide genetic variation is more predictive of complex heritable traits than GWAS hits (see for example Meuwissen, Hayes and Goddard, 2001). Additionally, not accounting for genome-wide variation may hide subtle effects of stratification and confounding in the data.

From the limited description, it is unclear how the binary-classification approach would treat continuous traits and predictions. In the paper, this has been circumvented, by substituting the continuous age and BMI by simulated a binary classification. For this simulation, the strong assumprion has been made that it could be predicted at perfect accuracy, meaning that each of these values provides one bit of information. In this context, we would like to note that for example BMI is a trait with a large environmental component, meaning that it has limited predictability from the genome. The cited standard error for a genomic estimate actually refers to a standard error of a linear regression effect, not of a prediction residual. Age prediction by methylation would require a different experimental assay.

Additional points that should be addressed:

- State in the methods clearly on what data set the GWAS had been performed and using what model.

- Provide appropriate references to figures and supplemental material from the main text.

- Make all figure captions concise and self-contained.

Reviewer #3 (Remarks to the Author):

This paper reports research demonstrating the feasibility of combining analysis of 3D facial images with genotyping of facial features to provide a new tool for individual identification in forensic settings. Its results are novel and will be of broad interest.

Appreciating the potential power of a forensic tool that combines DNA analysis with imaging technology, which is increasingly sophisticated and prevalent, authors stress the need to proceed cautiously. Authors identify the limitations of their findings and declare (most clearly in the Supplemental materials) the still to be fulfilled need to assess the validity and reliability of the method. At the same time however other passages undercut this caution.

1. Comments here address ways to strengthen the message of caution: 1) The phrase proof of concept, used in supplementary materials to characterize the research, might be used also in the paper's introduction and possibly the abstract to clarify the stage of the research; 2) To reduce the potential for misunderstanding about the technology's readiness for crime-fighting uses, authors might consider replacing the words suspect and perpetrator, each of which appear only once, with more neutral terms, such as one the paper also uses: person of interest; 3) Authors might consider rephrasing the end of the sentence, which appears on both pp. 10 and 28: "Furthermore, these methods also raise undeniable risks of further racial disparities in criminal justice that warrant caution against premature application of the techniques until proper safeguards are in place…" As written, it might encourage a misreading that the technology has already reached the stage where details of the method's bureaucratic implementation demand attention. However, as lines 972-977 clarify, before any such move is possible, researchers have to further substantiate the method's reliability and validity; as well as iron out several other issues, such as to what populations can it be confidently generalized and, a point they have not mentioned but which seems pertinent, what elements of 3D imaging require trans-local standardization; and, 4) Lines 394-403 raise similar concerns to (3) and should be revised to clarify that the procedures they discuss become relevant only once efforts to demonstrate the methods' reliability and validity are successful.

My point in (3) and (4) is not that the authors should not alert interested parties about what implementation might require. Rather it is that by discussing implementation without qualifying it, the authors deflect attention away from the fact that the manuscript's positive findings are still preliminary, which inadvertently may contribute to the hype that genomic technologies habitually attract. The following sentence raises similar concerns. "Considering our results so far, we strongly advise against using the single "best" match as an exact image of the person of interest." This passage also raises an additional question: what's the basis for this advice? If based on observations (which have not been described), it suggests that such recommendations should be based on empirical inquiry, a process that presumably would take account of important differences between the behavior of the system under laboratory conditions and the rather different context of routine police use.

2. Similarly, the basis for the recommendations offered at lines 397-400 should be clarified. Maybe "generating and presenting multiple images" will communicate "variability in accuracy" which "might help avoid prematurely targeting a single individual… ." Arguably, however, absent research, likely by psychologists, it remains to be seen how potential users will manage hundreds of candidate images that differ from one another only in minute details.

3. Machine learning: In light of growing concerns about researchers inadvertently integrating racial bias into machine learning, authors might 1) explain in more detail how the training phase of their work handled this problem and reflect on any weaknesses in their strategy; 2) consider what biases other than racial researchers might unintentionally contribute to these algorithms; and, 3) reflect on the implications for their new method of the fact that problem of how to monitor and

adjust self-learning AI systems as they evolve remains daunting and is likely to be especially so when implemented in potentially resource-strapped local or state governments.

4. Lines 347-349 clarify an important aspect of the method's logic. Readers might benefit from a short addition here elaborating why "higher genomic diversity in the gallery… against which identity is sought, leads to an easier recognition task, but does not necessarily imply a better ability to recognize an individual.

5. The argument and purpose of "Text S2: Genomic research challenges" require clarification. A minor problem with this section is inattention to whether the cited research provides evidence about changes in public sentiment or changes in scholarly sentiment. More serious is the failure to make explicit that the account draws on research and commentary concerning the use of genomic information for medical research, not for forensics. Practically speaking this difference might become irrelevant; restricting genetic data to medical uses might prove as inadequate as de-identification was at protecting privacy. Nonetheless, at least for now it seems likely that the distinction will not be lost on the public. Are arguments about sharing genomic data based on public trust and solidarity – the success of which convincing people to share genomic information for medical research remains unproven—likely to convince them that their data should also be available for forensic identifications?

Minor points

1. Describing the human face as a trait while at the same time describing component features of the human face as traits is confusing.

2. Fusing and fuser are not terms I am familiar with in this context. Could authors clarify how they are using them?

3. Possible typos or wording errors:

i. Facial shape features, were obtained by first applying a generalized Procrustes analysis…

ii. This approach generates a nested series of facial shape features, which are then used in multivariate association studies to identify related molecular features and in this work as input for facial classifiers.

iii. The strongest statistical evidence, and this for all three aspects, was found in the lowest….

iv. It was anticipated and observed that only in the combination of multiple and different molecular features, strong increases in specificity were achieved.

v. Although many individuals are identified within a top 10% of a sorted gallery, only a few are identified so uniquely.

vi. Subsequently, face-to-DNA classifiers were built and each of them, essentially, labelled facial images into possible categories of a specific genetic feature….

Reviewer #4 (Remarks to the Author):

Although I am very impressed by the quality and innovatively of the study that is described in the manuscript Facial Recognition from DNA, submitted by Peter Claes et al., I am not convinced that this study gives the answer to the forensically most relevant question: whose DNA is this?

Confronted with a criminal offence from which we have (nearly always) very limited amounts of DNA that could belong to an unknown perpetrator, the ideal scenario would be one based on which we can reliably reconstruct the facial characteristics of the unknown perpetrator, while still retaining sufficient DNA for a DNA-to-DNA match.

It is against this context that I find this manuscript not very realistic and convincing. If we assume that there is an almost unlimited amount of facial variation, the method that is outlined here, is still based on reference data from a very limited amount of facial profiles (this variation). Furthermore, it requires a substantial amount of genome wide dispersed SNPs in order to be able to apply the model proposed by the authors.

I also find the manuscript (unavoidably, I understand) very theoretical and very speculative, and it does not convince me that it will lead to the very desired (in a criminalistic context) DNA-to-face prediction. In this respect, all the models, scores and numbers of PCA’s are of little value if they do not facilitate its final purpose: based on these SNPs we were able to reconstruct this face, which can only match to this individual.

REVISION NOTES

We thank the reviewers' constructive feedback that have helped improve and strengthen the manuscript. Below is a point-by-point response in blue to each of the reviewers, with the original reviewers' comments in italic. We also provided the original manuscript with changes highlighted.

We have gone through the entire manuscript carefully and adjusted every relevant sentence to clarify our meaning. In the submitted manuscript and in the responses to the reviewer, we replace the words "unknown DNA" with "unidentified DNA", where possible, as it can lead to misleading interpretations among geneticists: an "unknown DNA" means that it has not been genotyped and, therefore, the DNA profile is "unknown". Also, the original use of an "unknown DNA" was more related to biometrics as in unknown identity. Furthermore, we have replaced "face-to-DNA mapping" with "face-to-DNA classifier" to make more explicit the fact that our mappings are classifiers. We have added this information to the title as well.

Importantly, we would like to point out already a few important aspects with reference to the contextualization of our technique and its novelty. The aim of our method is not to replace, not to reject, and not to de-emphasize the benefits of forensic techniques, such DNA profiling and DNA phenotyping, whose successes have already been vastly validated and reported[1,2]; instead, our approach represents an additional and complementary tool that can be used as further support in DNA investigations. Given an unidentified DNA sample, what are the options to establish the identity of the individual? To lay out more clearly the positioning of our technique, Figure 1 has been expanded. Following Figure 1 from top to bottom, given the unidentified DNA profile, referred to as the probe DNA, investigations lead to two possible outcomes: the person is identified, or the identification fails. The first attempt is to match the probe DNA sample to the DNA sample of a person of interest. If this approach does not lead to identification, then the same probe DNA is matched against DNA profiles with known identities enrolled in a database (genetic database). However, it is often the case that the repository under enquiry does not include the DNA sample that exactly matches the probe DNA sample, and the identification effort fails again. At this point, our approach could be of help. In our pipeline, the probe DNA is matched against faces with known identity, without the need to predict the face first. Rather than matching the probe DNA against DNA profiles stored in a genetic database, the challenge is to match the probe DNA against 3D facial images in a phenotype database. Finally, as a last resort, DNA phenotyping ideally returns a profile to show to the public; however, the current state of DNA phenotyping has not achieved this ability yet: because eye color, hair color or ancestry alone are not enough for people to recognize.

Reviewer #1 (Remarks to the Author):

For the paper 'Facial Recognition from DNA', the authors describe a new methodology in the prediction of an unknown individuals face from DNA. The authors describe a very interesting alternative to phenotypic prediction from genotypic information, rather than describing a face entirely from DNA, the authors propose to use the backing of established biometrics ***to 'compare' a face to a set of faces (using only trained genetic identifiers)*** *to see if a match is a) present in a group of faces, b) assign a strength of match to one face.*

We thank the reviewer for the positive feedback on the methodology proposed.

With reference to the sentence in bold, we are proposing a system that can be used to identify or verify an individual's identity based on his or her DNA profile (e.g. from blood or a hair sample found at a crime scene); therefore, our system 'compares' an unidentified DNA sample directly to a set of faces and face-to-face matching is not involved in our pipeline. Essentially, it circumvents the need to predict a face from DNA. We reworked the introduction and Figure 1 to better situate the novelty of the work and how it fits with the existing approaches.

Although there is merit to this approach and the amount of data in this paper is quite substantial, this reviewer feels that some of the data detracts from the overall message, i.e. there is too much going on while reading the paper that clouds the overall approach, which is the whole point of the article. In some instances, there is also not enough genetic data to back up some of the overloaded points in this paper, therefore the reviewer feels that if the paper was more structured and not as convoluted, it would get the message across much better. Below are suggested improvements for restructuring of the paper to reach the standard of publication in this journal as in its current state, it is not acceptable.

We thank the reviewer and we agree on his/her constructive comment. With reference to the complexity of the text, the manuscript has been revised and extensively simplified. Also, Figure 1 has been restructured to contextualize more clearly the positioning of our method.

Abstract:
I think the overall abstract is an overstatement of the methodology when comparing to the prediction of unknown face from DNA, as they are different approaches.

As emphasized at the beginning of this review, we are conscious of the profound difference in methodology between our method and DNA phenotyping[1]; importantly, our aim is not to tone down the relevance of DNA phenotyping as investigative tool used by law enforcement. Instead we propose a method as complementary and additional forensic tool. By offering this approach as another option, we do not mean to imply that DNA phenotyping should be given less merit or be viewed as having less utility than is currently recognized. We have deleted "alleviating many complexities involved" from Line 34 to avoid misinterpretation of our intended meaning.

Even with this suggestive approach of using machine learning to train how segments of the face look with regards sex, genomic background and SNPs, one must have access to a group of faces to compare the unknown DNA to, if not, then this methodology will not work, this obvious drawback is missing from here. If one had a group of faces to compare to, regardless of methodology, it is obviously easier to 'match' a person.

Regarding the "access to a group of faces to compare the unknown DNA to", the reviewer is correct in emphasizing the need for databases of facial images. In general, as stated in the discussion, devices like the Microsoft Kinect camera, the latest iPhone, and Microsoft Surface, are providing the means to capture 3D facial data (see https://www.theverge.com/circuitbreaker/2018/4/11/17226434/bellus3d-face-scanningiphone-x-app-3d-image-selfie and https://www.youtube.com/watch?v=dLsax142_WA). These examples illustrate the increasing availability of 3D facial images. The days in which 3D facial imaging

remained a laboratory experiment using expensive and large 3D scanning equipment are over, as more and more interest of the lay public is pushing these new devices to be used in user authentication and distributed via social networks. Furthermore, a substantial body of research in computer vision is focused on the extraction of 3D information from 2D images, with some of the seminal work[3] dating back to 1999. Today, strong results on this endeavor are obtained, e.g. http://cvldemos.cs.nott.ac.uk/vrn/, basically converting any existing 2D input to 3D. We explicitly state in the discussion that future improvements are needed and that the practical limitations need to be more fully investigated.

Introduction:
The author's suggestion of reframing the problem is commendable but perhaps rephrasing it would be better. Currently it says 'instead of predicting an ungiven face from DNA, is it possible to match a given face to DNA using face-to-DNA mapping' these are two different questions, it should read…instead of predicting an ungiven face from unknown DNA, is it possible to match unknown DNA to a given face using face-DNA mapping', after all it is DNA you have at a crime scene etc. and that is the question on hand – can you get a face, or face match from DNA.

We agree with this comment. That paragraph has been revised and the mentioned sentence has been replaced with:" […] Instead of matching a probe DNA to a database of known DNA profiles, we propose to match it against a database of known facial profiles. […]".

Next, the classification of faces by molecular aspects. For sex, did you actually look at genetic information to classify the DNA was male/female – this is not stated, it looks like you simply accepted the m/f statement in questionnaire/images and trained from there.

We thank the reviewer for pointing this out; it was not clarified in the text, sex was X-chromosome homozygosity/heterozygosity based from the genome. We add this information in the Materials and Methods.

Obviously, if you had unknown DNA and a 'group' of images to compare to, straight away you can at least remove the opposite sex (from the DNA sample) from you line up list, nothing novel here. However you are suggesting that using certain segments of images on the face, some may be more masculine/feminine, what are the criteria for something more masculine/feminine, do you have data to back these up? You state 'sex affected the masculinity/femininity of many features across the face, can you elaborate?

It is important to note that the proposed methodology relies only on facial images in a dataset that does not need to be augmented with other meta data, such as sex, or even genetic data. I.e. all data required to match with the DNA profile, such as sex, genomic background, individual genetic loci, etc. are estimated from the facial images directly. Therefore, random image databases, as pulled from the internet e.g., are becoming of use. This implies that information like sex and the other molecular features used in the work are deducted from the facial images, and to this end, the parts in the face that are most sexually dysmorphic aid most in the deduction of the features from the images. Hence the identification of facial parts, most strongly associated first, followed by a classifier using these parts to estimate the molecular feature from the face. The fact that all information is deducted from facial images,

without the need for other meta or genetic data (which is also in contrast to the work of Lippert et al.) has been stated more firmly in the manuscript.

For both cohorts, sex effects on facial morphology are visualized as normal displacements; these are listed for each of the 63 segments in the **online** page (https://mirc.uzleuven.be/MedicalImagingCenter/ImagingGenetics/FacialRecognitionFromDNA/) which was included in the first submission. More precisely, the normal displacement plots are in **global cohort** and **euro cohort** files online, for the GLOBAL and EURO cohort, respectively. Our findings are in line with the literature on sexual dimorphism displayed on the human face[4,5].

How does the algorithm 'see' these masculine/feminine features, is this something you can better explain/categorize?

For each genetic aspect, the outcome of the association study is the significance p-value (p) for each of the 63 facial segments. The shape features from the segments with statistical evidence reaching the selective thresholds of p<=1x10^-3 and p<=5x10^-5 in the GLOBAL and EURO cohort, respectively, are concatenated and used as the predictors for the face-to-DNA classifiers. For sex, the p-values from each segment fall below the selective threshold; as such, the algorithm "sees these masculine/feminine features" as it uses the concatenation of the shape features from every segment to map sex from the face.

The nested approach to facial shape is wonderful and the method has great promise,

We are grateful to the reviewer for approving the hierarchical segmentation.

however the authors seem to be missing the direct genetic connection and rely too much on machine learning (which will be affected by training sets). I understand you are trying to identify related molecular features to use as facial classifiers and that's fine, but we know sex and genomic background influence the face, this (and you) have already shown this. What is novel here is using the bayes matching approach, if from DNA you know someone is male, they are European (based on the genomic PCs you describe), it is the segments of the face and their association (based on your training) that is more interesting to give a match to your 'group' line up of individuals and in essence give a score – that method is novel as it ties in a genetic test to a biometrics comparison/rank score. If you restructure the paper simplifying this, it would make for a much nicer read.

On the one hand, we are pleased to see this line of thinking by the reviewer, also stating the novelty behind it. On the other hand, we regret to see that is exactly what our approach is about, and therefore realized that we should indeed restructure and simplify the paper to make sure that further confusion is avoided. We have reworked the paper extensively to achieve this goal.

Also your sentence 'this creates the novel ability to identify (who is this person?)… should be 'who is this person from this group of people?', you're overstating here. I see you state it is a 'group' towards the end of the lengthy sentence but please shrink, it's very convoluted.

The intention behind this sentence was to introduce the standard identification setup used in biometrics to evaluate, together with the verification setup, the performance of

our recognition system. In order to handle the complexity of this sentence, we reduced this block.

Remember it is not the same thing, making a face from DNA to give an identity (what you state as a version of DNA phenotyping) is different than identifying someone within a group with their DNA, different questions, not a reframe of the same thing.

Although there is strong overlap with our approach to predicting a face from DNA, we agree with the reviewer and have reworked the introduction to our approach without "reframing" it as the same thing.

The whole section on using two cohorts does not seem beneficial, at least shrink the GLOBAL section – you stated yourself in the discussion that 'recognition in the GLOBAL cohort is limited to an individual's population background' so perhaps this should be less lengthy as its been pretty much published before.

We have reduced in length the paragraphs of both the GLOBAL and EURO cohort in the Results. However, we respectfully disagree with the reviewer. In light of the novelty of our approach and the potential areas for confusion, we strongly think the GLOBAL cohort has merit and deserves to be emphasized. It is true that genomic background and ancestry have been used extensively in forensics. However, in this work we deduct this information from facial images, so that it can be matched to what the forensics community is accustomed to using. Also, by including the section on the GLOBAL cohort, the findings from the EURO cohort are even more accentuated, with emphasis to individual SNP contribution.

This approach of using DNA to check the face of a group of people to see if there is a potential match or not, should only be on genetic information or 'face snps' you use with sex/background information (however generated) there to dictate which algorithm/training set (use GLOBAL or EURO) to use for the SNP data classifiers to enable the generation of a bayes score in that subset of people. I would suggest the authors streamline their approach based on what they would have, which is the DNA and how they can use their training data to match after they get the raw data from the DNA and not get so caught up in all the different approaches, associations, gwas hits on
100k snp data that may have nothing to do with the facial segment – could be chance etc. (although FDR is in place) that they have put into the paper.

We revised Figure 1 to contextualize better our pipeline in current DNA investigations. We also revised the introduction to streamline the method used, as well as the main text in general.

I suggest avoiding too much focus on DNA-inferred classifiers right now (age and BMI) until there is more information and accuracy testing on their reliable prediction from DNA. One would hope that in time it will be a more stable trait to predict, however right now it may be best to cover your face match by correcting for multiple ages/weights instead of trying to build it into the model as a classifier – why – well look at your own passport, that image is not a current photo, so this all comes down to what group of face photos you are comparing your match DNA to. You are certainly missing pigment here and this should certainly be included as an obvious classifier as you have stated in the paper, due to current genetic information and data supporting its prediction from genetic markers.

We agree with the reviewer that the current prediction of age[6] and BMI[7] from DNA is far from perfect. Moreover, we moved the section “Simulated DNA-inferred age and BMI” to the Supplementary Material to tone down the emphasis on age and BMI contributions. With respect, we think the contributions of age and BMI are still of some scientific value in contrast to practical value for both cohorts. Following Figure 4, Table 2, and Figure S8, the contribution of age and BMI is not as substantial as the 382 significantly associated genomic background components and the 32 significantly associated SNPs in the GLOBAL and EURO cohort, respectively, but still tangible. In Figure 4 and Table 2, the results for both cohorts are presented in a cumulating way, and BMI and age have been added at the very end. We would like to stress that the results for both cohorts are solid even if age and BMI are excluded. This is scientifically of interest, to show that we can get results without age and BMI, but, if available, to what extent do they contribute?
We agree with the reviewer that “correcting for multiple ages/weights” would be an interesting investigation, in particular to check how age affects identification, but this effort does not conciliate with the scope of the current work. We would like our face-to-age classifier to work on different age/BMI faces; correcting for age/BMI does not seem realistic, and even if one would like to, the age/BMI to correct for in practical random facial image datasets is unavailable (at least we would like to assume that this meta data is provided).
The reviewer is right in that skin pigmentation can also be incorporated when using facial image texture in addition to shape; we also discuss this in the Discussion and we envision an increase in performance.

I am glad to see the authors push for scientifically justified and peer-reviewed processes to be required for facial prediction/matching. Be careful to also include that forensic DNA samples are limited, and where other facial profiling companies produce array data with thousands of SNPs to produce their ‘facial renditions’ – which is basically sex and ancestry, this is not optimal. Clearly optimizing what can be generated genetically for the DNA probe for each of the classifiers is an important task. The choice of SNPs here will be paramount, as you are correct, it may be science fiction to produce a face from DNA and easier to match a face with known classifiers from DNA as your method proposes, however how do you see this being done, a random array as you have discussed that may not include the correct causal markers or a more specific design that tells the user, which (GLOBAL or EURO) training set to use for your facial matching algorithm (choice of classifiers to use) and score fusing.

We acknowledge that in forensics it is often difficult to work with the frequently limited amount of DNA available, and much more work is to be done in the future, even for our approach to properly define the information, amount of DNA and arrays needed. At this stage, we used full genome data. We have added some idea of moving forward on these issues in the Discussion.

It’s a pity to see GWAS data lost in the context of this paper due to doing ‘too much’.

The GWAS results are interesting, but these do not represent the core of the current paper; the GWAS paradigm was published in the work of Claes et al.[8], where functional support was also provided.

Also the choice of the peak SNP is not ideal, were there any other parameters examined other than lowest p value? Of course, it is unlikely these are causal just by chance, and therefore makes this analyses disappointing. Perhaps comparing allele frequency between the snps in these loci would be more beneficial. At least something more than just lowest p value (after >0.5 LD inclusion), as it would drastically affect the association of the alleles (whether dominant or recessive) in the SNP classifier performance with the facial segments – which to me is the most interesting classifier. Even using regulomedb to propose/rank regulatory suspected variants (if the SNP were intronic/intergenic) would be useful rather than randomly choosing the highest associated SNP.

This manuscript presents the first-generation output of the method. We agree that peak SNPs are not the ideal SNPs and that alternatives are possible; however, these SNPs are not randomly selected. We rely on the fact that these SNPs are in LD with causal variants and that these peak SNPs still contain information about the causal variants. We acknowledge that in GWAS, association does not necessarily imply that a marker SNP is a causal SNP. To arrive at the point in which causal variants are identified, it will take additional functional research and this is outside the scope of this paper. As stated by Edwards et al.[9] in 2013*: "The next major challenge lies in moving from associated tag SNPs to finding the strongest candidate causal variants and then identifying their target gene(s)."* However, the results based on selecting peak SNPs do show that these bear good signal—for the biometric challenge presented—where proper separation of data into discovery, training and testing has been addressed.

Lastly, when assessing the performance on your test set, what is your marker for sex from the DNA profiles of these individuals? Presence of Y SNPs? Can you explain?

For all participants, sex was X-chromosome homozygosity/heterozygosity based from the genome. This information was added to the Materials and Methods for both cohorts.

It would also be nice if a subset of individuals from the test dataset (both global and euro combined, n=571) were randomly left out, so that you could assess those samples without knowing which dataset they belonged, perhaps a 2-step classification score, one using the 382, the other using the 50 classifiers, for all 571 individuals DNA.

The EURO sample is part of the GLOBAL sample; any Europeans left out in the GLOBAL dataset are going to perform exactly the same as any Europeans in the EURO dataset. The other way around, we admit that the GWAS was run on the EURO sample and it is known that applying found SNPs across different populations is difficult and poorly understood; therefore, we expect a performance drop on the GLOBAL cohort for non-European descent in the EURO models.

Minor comments:
As you use some 1k genomes for your population structure assessment, would be best to further describe this and reference the set. I could not find this, other than you used it for imputation, and you mention its use in Figure S2 (but didn't reference it).

We have added this reference[10] in the caption of Figure S2. Importantly, in Figure S2, the 1000 Genome samples, are used solely to illustrate here and have not been used

throughout the analysis in the main manuscript. Instead the Hapmap 3 project was used for population structure assessment in the GLOBAL cohort.

Overall, the idea of using matching scores based on genetic data quite like biometric matching scores is appealing and therefore this novel idea has merit,

We appreciate the reviewer's positive consideration of our approach in general and its novelty.

however, the structure and approach of the paper needs fixing. For example, there is lacking data on how the test data would actually be assessed with regards their DNA and not simply using questionnaire info (as seems to be the case for not only bmi and age, as they are inferred, but also for sex right now). If the whole point of the method is to give values/strength to a match, then that should be the focus of the paper, from DNA input, what and how, to match output, this is stifled by the many other components of the paper.

As specified in previous replies, the manuscript has been revised and accessory details have been excluded. We hope this simplified version of the manuscript allows for a smoother and clearer read.

Reviewer #2 (Remarks to the Author):

The paper presents an approach for identification of individuals from DNA by predictive modeling for multiple phenotypes.
The problem is of importance, both for forensics, as well as in the light of personal privacy of individuals who share anonymized genomic and medical phenotype data for research.

We appreciate that the reviewer emphasizes the serious forensic and privacy challenges that our preliminary results bring to concern.

The approach shares similarity to ref. 4, but uses a combination of binary classification instead of a combination of the regression models used in ref4. However, the limited technical details and comparison between the approaches provided in the manuscript make it hard to guess the exact differences and technical contributions of the paper. This lack of clarity and technical detail makes it hard to judge novelty and limits reproducibility.

We would like to streamline the difference between our method and the work of Lippert et al.[11]:

1) following Figure 1, Lippert et al. predicts the face and then adopt the phenotype-vs-phenotype matching, while in our case the matching is at the DNA level. We do not predict any face, we reframe the computation challenge, meaning that we make the shift from "face extrapolation" to "face-to-DNA classifier" which is a classification problem in machine learning;
2) one important strength of our work is that we are not taking the conventional approach, since the recovery of facial shape from DNA remains unresolved and simply driven by sex and ancestry, as proven in preliminary studies[11,12].
3) Lippert and colleagues use a linear model to map the genotype to phenotype while these are highly non-linear phenomena; our classifiers are non-linear, so every non-linear delineation is considered and the way these classifiers are

combined is again non-linear and allows for some more complexity than just the linear prediction model.
4) Most importantly, the work of Lippert et al. still relies on other data besides facial images. In this work, facial images are the only input to the system, without the need for additional meta data or genetic data on the individuals in the gallery with known identity.

Therefore, if the reviewer is asking us to compare the outcomes and results, this cannot be done because the paper of Lippert et al. diverges in content to our proof of concept. Importantly, Lippert and colleagues[11] did not obtain the results in the EURO cohort because they did not use SNPs; one could refer to the results in the GLOBAL cohort in the best scenario.

The main reported finding in an evaluation on two different cohorts, one cohort of diverse ancestry, and a cohort of European ancestry, is that individual SNPs, which have been found significant in a GWAS, contribute to identification performance. However, while this finding would be exciting, in their analysis on the European cohort, the authors only take into account 4 PCs to model tagging of genome-wide genetic variation without showing empirical evidence that additional PCs do not tag phenotypic variation beyond the first 4 PCs. Such empirical evidence is required, given that it has been found in multiple studies of heritable phenotypes that genome-wide genetic variation is more predictive of complex heritable traits than GWAS hits (see for example Meuwissen, Hayes and Goddard, 2001). Additionally, not accounting for genome-wide variation may hide subtle effects of stratification and confounding in the data.

The main use for the PCs in the EURO cohort was to provide for a good stratification in a GWAS effort, and this following standard protocols in GWAS studies; by visually inspecting the PCs, we also saw that up to the 4th PC we handle this stratification. Furthermore, when testing the first 20 PCs against facial variation in the EURO cohort, no more than the first and the fourth PC were yielding associations (not even the second and the third). In our approach, an association of a molecular feature to facial variation, was a selector for building a related facial classifier. Therefore, only associated features were used to extract information from facial images to classify individuals.

From the limited description, it is unclear how the binary-classification approach would treat continuous traits and predictions. In the paper, this has been circumvented, by substituting the continuous age and BMI by simulated a binary classification. For this simulation, the strong assumption has been made that it could be predicted at perfect accuracy, meaning that each of these values provides one bit of information. In this context, we would like to note that for example BMI is a trait with a large environmental component, meaning that it has limited predictability from the genome. The cited standard error for a genomic estimate actually refers to a standard error of a linear regression effect, not of a prediction residual. Age prediction by methylation would require a different experimental assay.

We agree with the reviewer that the accuracy for DNA-based age[6] and BMI[7] predictions have not met yet accuracy values to be applied in forensics. We have deflected attention on simulated DNA-inferred age and BMI by shifting this section from the main text to the Supplementary Material. With respect, we did not circumvent the problem of continuous variables by implementing a binary

classification. Instead, our rationale was to use a simple model (SVM) which is available in every package of common programming languages (MATLAB, python, R). By using a simple and well performing classifier, we establish a benchmark face recognition pipeline from DNA predictable traits; from this solid baseline performance, more sophisticated predictive models can be explored. Also, by implementing SVM for binary classification, allows to use the posterior probabilities as matching scores; with regression models instead, the face-to-DNA classifier would output a continuous value and then the problem would be how to design the matching score. We acknowledge that the choice of a threshold T is "crude" (as we mention in the Materials and Methods), but our preliminary results indicate that this is a good way to incorporate continuous variables into our first-generation outcomes. In the methods, we mention this aspect and indicate that more advanced approaches are open for exploration.

Additional points that should be addressed:
- State in the methods clearly on what data set the GWAS had been performed and using what model.
- Provide appropriate references to figures and supplemental material from the main text.
- Make all figure captions concise and self-contained.

We thank the reviewer for addressing these adjustments. We have added in the methods that the GWAS had been performed in the EURO cohort under an additive genetic model (AA=0, Aa=1, aa=2), after correcting for confounding variables including sex, age, BMI, four genomic PCs, and the dataset identifier.
Because the initial submission contained mismatches between the references to the figures/texts in the Supplementary Material, we correct these in the new submission.
We have made all the figure captions concise and self-contained where possible.

Reviewer #3 (Remarks to the Author):

This paper reports research demonstrating the feasibility of combining analysis of 3D facial images with genotyping of facial features to provide a new tool for individual identification in forensic settings. Its results are novel and will be of broad interest.

We thank the reviewer for the positive feedback.

Appreciating the potential power of a forensic tool that combines DNA analysis with imaging technology, which is increasingly sophisticated and prevalent, authors stress the need to proceed cautiously. Authors identify the limitations of their findings and declare (most clearly in the Supplemental materials) the still to be fulfilled need to assess the validity and reliability of the method. At the same time however other passages undercut this caution.
1. Comments here address ways to strengthen the message of caution: 1) The phrase proof of concept, used in supplementary materials to characterize the research, might be used also in the paper's introduction and possibly the abstract to clarify the stage of the research;

The term "proof of concept" has been introduced in the abstract and introduction.

2) To reduce the potential for misunderstanding about the technology's readiness for crime-fighting uses, authors might consider replacing the words suspect and perpetrator, each of

which appear only once, with more neutral terms, such as one the paper also uses: person of interest;

> We agree with the reviewer's suggestion and our current submission adopts "person of interest" instead of "suspect" and "perpetrator".

3) Authors might consider rephrasing the end of the sentence, which appears on both pp. 10 and 28: "Furthermore, these methods also raise undeniable risks of further racial disparities in criminal justice that warrant caution against premature application of the techniques until proper safeguards are in place…" As written, it might encourage a misreading that the technology has already reached the stage where details of the method's bureaucratic implementation demand attention. However, as lines 972-977 clarify, before any such move is possible, researchers have to further substantiate the method's reliability and validity;

> For our pipeline, the reviewer is right in that our results are still preliminary and still far from the stage of harm, as declared in the Discussion ("[…] our results are preliminary and on well-defined data cohorts"). The underlying message is that if our pipeline is applied without proper safeguards, caution should be taken as described in the Discussion and in the Text S3 of the new submission. We have emphasized the terms "proof of concept" and "preliminary results" already in the abstract and introduction.
>
> We would like to point out that researchers are already using DNA-based face rendition even though it is not validated as in Parabon nanolabs (see https://snapshot.parabon-nanolabs.com/). As members of a scientific community, it is our responsibility to highlight potential harms in using technologies like these.

as well as iron out several other issues, such as to what populations can it be confidently generalized

> The reviewer is likely referring to the limitations that may arise from using the two cohorts. In the GLOBAL cohort, we are limited by the ancestry captured with our reference data (HapMap 3 Project). We suggest that different spaces can be used (intercontinental and more European data). Moreover, in our work the identification of an individual has been proven only on a European population and not on any other locally-scaled population. Investigating different genomic background spaces for the GLOBAL cohort and identify an individual in the context of another single homogeneous population are certainly of high value for future research but have not been addressed in this manuscript. However, we have added these limitations to the discussion

and, a point they have not mentioned but which seems pertinent, what elements of 3D imaging require trans-local standardization;

> A similar concern has been raised by Reviewer 1. We remind the reader that 3D imaging has been embedded more and more for user authentication and distributed via social networks. The latest technologies provide the means to capture easily 3D faces of the user and the 2D-to-3D imagery shift is tangible.

and, 4) Lines 394-403 raise similar concerns to (3) and should be revised to clarify that the procedures they discuss become relevant only once efforts to demonstrate the methods' reliability and validity are successful.

The results we refer to in those lines are depicted in Figure 4, where the best match (rank 1) gets always low recognition rates and better performances are observed only for rank 20. Therefore, we prefer deviating from the single best match mentality as a single best match is hardly achievable. We have added more clarification in the Discussion.

My point in (3) and (4) is not that the authors should not alert interested parties about what implementation might require. Rather it is that by discussing implementation without qualifying it, the authors deflect attention away from the fact that the manuscript's positive findings are still preliminary, which inadvertently may contribute to the hype that genomic technologies habitually attract. The following sentence raises similar concerns. "Considering our results so far, we strongly advise against using the single "best" match as an exact image of the person of interest." This passage also raises an additional question: what's the basis for this advice? If based on observations (which have not been described), it suggests that such recommendations should be based on empirical inquiry, a process that presumably would take account of important differences between the behavior of the system under laboratory conditions and the rather different context of routine police use.

We thank the reviewer for inviting us to clarify this important point. We agree that our results are "proof of concept" and that our study is a "proof of concept face-from DNA recognition research", as specified in the abstract, introduction and discussion. However, we emphasize that our approach pulls back the "best match" way of thinking. When looking at our cumulative curves in Figure 4 and performances per molecularly derived feature in Figure S8, rank 1 recognition rate is always low and good results are only observed at rank 20. We have made this explicit in the Discussion to make sure the reader knows to what we are referring and what needs to be improved in the future.

2. Similarly, the basis for the recommendations offered at lines 397-400 should be clarified. Maybe "generating and presenting multiple images" will communicate "variability in accuracy" which "might help avoid prematurely targeting a single individual… ." Arguably, however, absent research, likely by psychologists, it remains to be seen how potential users will manage hundreds of candidate images that differ from one another only in minute details.

We accepted the reviewer's suggestions and changed the mentioned lines accordingly in the new submission.
The reviewer points out an interesting aspect by referring to the human capacity to discriminate individuals based solely on minute differences among a vast group of faces. In the work of Phillips et al.[13], the authors compare the performances in face recognition between computers and humans (i.e., untrained people who had no professional experience with face recognition). In their verification task, humans and machines were given a pair of images or videos, with each image or video containing one face. The humans and machines had to respond how likely the two faces were of the same person. They acknowledge that the main difference between measuring performance of humans and machines is the number of face pairs that can be

compared. While a computer can handle millions of face pairs, the maximum number of face pairs that an individual can rate in an experiment is about 250. The analysis shows that for difficult (i.e. different ambient and lightening conditions) still face pairs, humans are superior. In our approach, each face pair was different from the other, so we agree that in the work of Phillips et al.[13] the problem is simplified when compared to identifying someone from a list of hundreds somewhat similar faces. However, the minute differences among the group of listed faces represents the variability of the system that is exposed. Therefore, it is only correct to pin down the common aspects in the group of listed faces. We have added a paragraph in the Discussion regarding this point.

3. Machine learning: In light of growing concerns about researchers inadvertently integrating racial bias into machine learning, authors might 1) explain in more detail how the training phase of their work handled this problem and reflect on any weaknesses in their strategy; 2) consider what biases other than racial researchers might unintentionally contribute to these algorithms; and, 3) reflect on the implications for their new method of the fact that problem of how to monitor and adjust self-learning AI systems as they evolve remains daunting and is likely to be especially so when implemented in potentially resource-strapped local or state governments.

We correct for racial bias by looking at the EURO cohort alone because we do not have majority and minority ancestry classes. For the GLOBAL cohort we use ancestry which is defined by a publicly available dataset (HapMap 3 Project) in which the populations are balanced. The balance refers to the sample size of each population (around 100) of the HapMap 3 Project. The fact that in the GLOBAL cohort we use publicly available data, allows for a more stable rate population diversity. However, the projection may not be that balanced, and we may still have a bias towards a more European separation. Also, in the EURO cohort we do not use a reference panel. Moreover, as will be discussed in the following comment, a higher genomic diversity in the gallery does not necessarily imply a better ability to recognize an individual. The establishment and update of an AI system is challenging in the best of circumstances and is compounded in resource-poor settings. However, AI contributions are already tangible in several fields like, for example, AI contribution to health[14] and to environmental causes[15]. Digital forensics is a concrete reality; in the case of resource-strapped settings, the use of AI implies a strong understanding of the infrastructure requirements, access to adequate training data and additional needs, including IT and platforms. It is also important to realize that machine learning applications require high-profile experts able to produce and work on high-quality datasets used to train machine learning algorithms. In addition the availability of large databases requires substantial upfront investment. We acknowledge these challenges and highlight these more clearly in the Discussion.

4. Lines 347-349 clarify an important aspect of the method's logic. Readers might benefit from a short addition here elaborating why "higher genomic diversity in the gallery… against which identity is sought, leads to an easier recognition task, but does not necessarily imply a better ability to recognize an individual".

We thank the reviewer for bringing this part to our attention. With the cited sentence, we would like to emphasize that an increase in recognition power can be obtained in two cases. In the first case, an atypical face is easier to identify[16]; the more a person

stands out, the more he or she is recognizable in a crowd. The second case includes diversity, which is implicitly going to increase the number of subgroups in a group of people living together. Therefore, diversity leads to an increased recognition power at the level of subgroup—not at the level of the individual. For example, if a European person compares herself/himself to a group of European faces, recognition is more challenging than comparing the same person to a diverse group of people. In alternative, this person could stand out among Europeans if she/he has some specific facial traits. We have included more details on this issue in the Discussion.

5. The argument and purpose of "Text S2: Genomic research challenges" require clarification. A minor problem with this section is inattention to whether the cited research provides evidence about changes in public sentiment or changes in scholarly sentiment. More serious is the failure to make explicit that the account draws on research and commentary concerning the use of genomic information for medical research, not for forensics. Practically speaking this difference might become irrelevant; restricting genetic data to medical uses might prove as inadequate as de-identification was at protecting privacy. Nonetheless, at least for now it seems likely that the distinction will not be lost on the public. Are arguments about sharing genomic data based on public trust and solidarity – the success of which convincing people to share genomic information for medical research remains unproven—likely to convince them that their data should also be available for forensic identifications?

We appreciate why the reviewer would like to confirm that we have engaged the appropriate scholarly literature for the method we propose in our Text S2 (which now appears as Text S3 in the revised manuscript). We are confident that we have done so without overburdening readers with too much information that would detract from the focus of this manuscript or that might suggest to readers that legal and policy discussions are not needed in response to this potential investigatory tool. We remind the reviewer that this section was specifically focused on "genomic *research*" rather than a broad discussion on challenges with *law enforcement application* or all law and policy aspects.

The reviewer has expressed interest in a distinction between public sentiment and scholarly sentiment. We provided references in support of both. We pause to note that we would caution against any over-generalization about the diverse needs, interests, and preferences that individuals have about their personal data for different purposes than originally generated and in different contexts, at different times, and by different users. Empirical studies of genetic exceptionalism and attitudes on public policy (such as regulations to restrain police powers or regulatory oversight of direct-to-consumer personal genomics industry) have been hampered by the ELSI literature's medical emphasis. While the medicalization of the genome (a conceptual framework that the scholarly community as contributed to rather than merely observed) has been a recognized problem, the ELSI (ethical, legal, and social implications) research community increasingly calls into question the distinctions between medical information technologies and other consumer technologies. There are growing calls for reform of the United States' current sectoral approach to data privacy so that it is able to address a Big Data-driven and globally connected world and so that it aligns more closely to the European Union's General Data Protection Regulation 2016/679 (GDPR) and China's Cybersecurity Law and Information Security Technology – Personal Information Security Specification (e.g., Tim Cook, Apple CEO, publicly

called for data privacy regulatory reforms on October 24, 2018; see, e.g., https://www.inc.com/guadalupe-gonzalez/apple-tim-cook-federal-privacy-law-regulation-gdpr-europe.html). In the United States, the boundaries for genetic privacy has not been clearly delineated, and the continued uncertainty (in light of the Supreme Court's June 2018 decision in Carpenter v. United States, 585 S. Ct. __ (2018)) about the Third Party Doctrine's applicability to a Fourth Amendment inquiry would require us—and anyone else— to speculate about how this proof of concept approach would be evaluated. We decline the opportunity to do so at this time but note that one of us (a licensed attorney with experience publishing in forensic journals) is planning future work in this area.

A recent anecdotal example, however, supports our understanding that large segments of the public *do* support use of information for investigatory purposes. When law enforcement turned to investigative genealogy and non-forensic database (GEDmatch) to find a lead in the Golden State Killer investigation (or, if you prefer, the East Bay rapist or Visalia ransacker investigations), there was scholarly concern about the appropriateness of law enforcement turning to a database established with other purposes in mind[17–19]. GEDmatch terms of service were edited to permit, explicitly, law enforcement use of the site to solve serious violent crimes. While some individuals with GEDmatch profiles discontinued their use of the site and pulled down their data in response to the news of law enforcement use, the number of GEDmatch users has actually reportedly increased (as per personal communication with CeCe Moore during ISHI29[20] in Phoenix, AZ on September 25, 2018) following the news that it was useful in solving this heinous crime and could be useful in bringing other cold cases to closure. Many individuals feel a sense that by making their profiles available they are performing a community service. While some have suggested a potential legislative solution[18] to limit law enforcement uses to particular contexts (such as limiting the use to a tool of last resort when all other options have been exhausted), others[21] have proposed technological solutions (such as a cryptographic signature to the data file uploaded) to protect the privacy of direct-to-consumer genealogical sites intended for those who wish to share their information for genealogical but not law enforcement purposes.

Minor points

1. Describing the human face as a trait while at the same time describing component features of the human face as traits is confusing.

We keep the word "trait" referred to the face when used in the context of "complex trait" and "multipartite trait". To avoid confusion, we use "features" instead of "traits" when referred to "DNA-inferable traits" and "soft biometric traits".

2. Fusing and fuser are not terms I am familiar with in this context. Could authors clarify how they are using them?

Biometric systems can be designed to recognize a person based on information acquired from multiple biometric sources[22]. A fusion scheme (i.e. the fuser) combines the information presented by multiple biometric sources to increase the overall recognition power of the system. In our context, multiple matching scores from an individual's face against several molecular features, were combined (or equivalently, fused) into a single overall matching score by using a classification-based score fuser

(that is, a “classifier” in machine learning). The word “fuser” is a synonym for “classification model” or “classifier”; the word “fusing” is synonym for “combining” and “merging”. We have added the explanation of score fusing in the Materials and Methods as well.

3. Possible typos or wording errors:

The following sentences were changed:

i. Facial shape features, were obtained by first applying a generalized Procrustes analysis…

By first applying a Generalized Procrustes analysis (GPA) separately to the quasi-landmarks comprising each facial segment, followed by PCA, facial shape features were obtained.

ii. This approach generates a nested series of facial shape features, which are then used in multivariate association studies to identify related molecular features and in this work as input for facial classifiers.

We restructured this paragraph to make the read easier and the abovementioned sentence was deleted and replaced with: “[…] Essentially, we first divide the facial shape into several segments by using a recently developed phenotyping approach[8] and then extract shape features from each segment. We then find the strongest associations between these facial shape features and the range of molecular aspects of interest to predict the significantly associated molecular features from the face. […]”

iii. The strongest statistical evidence, and this for all three aspects, was found in the lowest….

For all three aspects, the strongest statistical evidence was found in the lowest …

iv. It was anticipated and observed that only in the combination of multiple and different molecular features, strong increases in specificity were achieved.

Strong increases in specificity were achieved only in the combination of multiple and different molecular features.

v. Although many individuals are identified within a top 10% of a sorted gallery, only a few are identified so uniquely.

Although many individuals are identified within a top 10% of a sorted gallery, performances on the single best match (rank 1), which are needed for an accurate recognition, remain limited.

vi. Subsequently, face-to-DNA classifiers were built and each of them, essentially, labelled facial images into possible categories of a specific genetic feature….

Subsequently, face-to-DNA classifiers were built and each of them labelled facial images into possible categories of a specific genetic feature…

Reviewer #4 (Remarks to the Author):

Although I am very impressed by the quality and innovatively of the study that is described in the manuscript Facial Recognition from DNA, submitted by Peter Claes et al.,

> We appreciate the reviewer's words with respect to the quality and novelty of our work.

I am not convinced that this study gives the answer to the forensically most relevant question: whose DNA is this?
Confronted with a criminal offence from which we have (nearly always) very limited amounts of DNA that could belong to an unknown perpetrator, the ideal scenario would be one based on which we can reliably reconstruct the facial characteristics of the unknown perpetrator, while still retaining sufficient DNA for a DNA-to-DNA match.
It is against this context that I find this manuscript not very realistic and convincing. If we assume that there is an almost unlimited amount of facial variation, the method that is outlined here, is still based on reference data from a very limited amount of facial profiles (this variation). Furthermore, it requires a substantial amount of genome wide dispersed SNPs in order to be able to apply the model proposed by the authors.
I also find the manuscript (unavoidably, I understand) very theoretical and very speculative, and it does not convince me that it will lead to the very desired (in a criminalistic context) DNA-to-face prediction. In this respect, all the models, scores and numbers of PCA's are of little value if they do not facilitate its final purpose: based on these SNPs we were able to reconstruct this face, which can only match to this individual.

> Following Figure 1, forensic challenges are present at any of DNA investigation methodologies and our approach is not trying to solve any of these. The purpose of the proposed work is to create a methodological connection which is not present in forensics yet.
> Our preliminary results push us to deviate from generating a single face, as very poor recognition rates are obtained at rank 1; by exposing X amount of faces, which are matching with the DNA profile to a certain extent and where the person one looks for fall within, allows for a more powerful ability to objectively reject suspects. When further validated, our method could be of use as an investigative tool; as such, it cannot be used in court, exactly as DNA phenotyping.
> Regarding the limited datasets when compared to the vast amount of facial variation, the reviewer is right in that our cohorts are well-defined datasets; nevertheless, with all respect, the reader should not underestimate the facial variation present in some databases, such as driving licenses.
> As stated in a previous response, we acknowledge that, in forensics, it is difficult to work with the often-limited amount of biologic material available, but this is the case for any DNA investigation. It is also correct that, at this stage, we use full genome data; a future challenge in forensics involves the ability to use our paradigm based on often limited and contaminated DNA material.
> We have added some ideas of moving forward on these issues and we address our limitations in the Discussion. In general, our results are certainly preliminary, but still of scientific value.

REFERENCES

1. Kayser, M. Forensic DNA Phenotyping: Predicting human appearance from crime scene material for investigative purposes. *Forensic Sci. Int. Genet.* **18**, 33–48 (2015).
2. Roewer, L. DNA fingerprinting in forensics: past, present, future. *Investig. Genet.* **4**, 22 (2013).
3. Blanz, V. & Vetter, T. A morphable model for the synthesis of 3D faces. in *Proceedings of the 26th annual conference on Computer graphics and interactive techniques - SIGGRAPH '99* 187–194 (ACM Press, 1999). doi:10.1145/311535.311556
4. Claes, P. *et al.* Sexual dimorphism in multiple aspects of 3D facial symmetry and asymmetry defined by spatially dense geometric morphometrics. *J. Anat.* **221**, 97–114 (2012).
5. Matthews, H. S. *et al.* Modelling 3D craniofacial growth trajectories for population comparison and classification illustrated using sex-differences. *Sci. Rep.* **8**, 4771 (2018).
6. Bekaert, B., Kamalandua, A., Zapico, S. C., Van de Voorde, W. & Decorte, R. Improved age determination of blood and teeth samples using a selected set of DNA methylation markers. *Epigenetics* **10**, 922–930 (2015).
7. Guo, Y. *et al.* Genetically Predicted Body Mass Index and Breast Cancer Risk: Mendelian Randomization Analyses of Data from 145,000 Women of European Descent. *PLOS Med.* **13**, e1002105 (2016).
8. Claes, P. *et al.* Genome-wide mapping of global-to-local genetic effects on human facial shape. *Nat. Genet.* **50**, 414–423 (2018).
9. Edwards, S. L., Beesley, J., French, J. D. & Dunning, A. M. Beyond GWASs: Illuminating the Dark Road from Association to Function. *Am. J. Hum. Genet.* **93**, 779–797 (2013).
10. Gibbs, R. A. *et al.* A global reference for human genetic variation. *Nature* **526**, 68–74 (2015).
11. Lippert, C. *et al.* Identification of individuals by trait prediction using whole-genome sequencing data. *Proc. Natl. Acad. Sci.* **114**, 10166–10171 (2017).
12. Claes, P., Hill, H. & Shriver, M. D. Toward DNA-based facial composites: Preliminary results and validation. *Forensic Sci. Int. Genet.* **13**, 208–216 (2014).
13. Phillips, P. J. & O'toole, A. J. Comparison of human and computer performance across face recognition experiments. (2013). doi:10.1016/j.imavis.2013.12.002
14. Wahl, B., Cossy-Gantner, A., Germann, S. & Schwalbe, N. R. Artificial intelligence (AI) and global health: how can AI contribute to health in resource-poor settings? *BMJ Glob. Heal.* **3**, e000798 (2018).
15. Hino, M., Benami, E. & Brooks, N. Machine learning for environmental monitoring. *Nat. Sustain.* **1**, 583–588 (2018).
16. Hill, H. *et al.* How Different is Different? Criterion and Sensitivity in Face-Space. *Front. Psychol.* **2**, 41 (2011).
17. Berkman, B. E., Miller, W. K. & Grady, C. Is It Ethical to Use Genealogy Data to Solve Crimes? *Ann. Intern. Med.* **169**, 333 (2018).
18. Ram, N., Guerrini, C. J. & McGuire, A. L. Genealogy databases and the future of criminal investigation. *Science* **360**, 1078–1079 (2018).
19. Syndercombe Court, D. Forensic genealogy: Some serious concerns. *Forensic Sci. Int. Genet.* **36**, 203–204 (2018).
20. 29 th International Symposium on Human Identification, September 24-27, 2018. In: Phoenix, AZ. in

21. Erlich, Y., Shor, T., Carmi, S. & Pe’er, I. Re-identification of genomic data using long range familial searches. *bioRxiv* 350231 (2018). doi:10.1101/350231
22. Ross, A. A., Jain, A. K. & Nandakumar. *Handbook of Multibiometrics*. (Springer-Verlag, 2006).

Reviewers' comments:

Reviewer #1 (Remarks to the Author):

I would like to thank the authors for taking into consideration my recommendations, and for providing constructive comments and amendments in their reply and the edited article submission. I believe the re-structuring of the paper is very well done, and the flow of text for a new and highly interesting method is excellent, well done. Below are some minor comments to fix/clarify but overall this manuscript is nearly ready for publication in this reviewer's eyes and I think the community will benefit immensely from its publication.

Minor edits:

1. Biological runs off the tongue a little nicer than biologic for the entire text, but this is up to the author to change.

2. I would edit the line to "DNA based prediction of phenotypes (hair color, eye color), and ancestry can be used to narrow down the pool of candidates onto which to perform further investigations." Ancestry is seen as independent to phenotyping classifications (not collective) in this context as it is not an externally visible trait per say.

3. I don't think the line 'for the first time and in contrast to DNA phenotyping,' is needed, there is no prediction model for facial morphology yet in FDP so why say in contrast to this. Yes you have shown each snps affect in your method which is novel but you are not exactly predicting specifically an individuals or subgroup as you indicated earlier in the text that phenotyping does. Best to change it to, 'for the first time by individual genetic loci …'

4. change to "We discuss how this work contributes to the development of new applications…"

5. change to "We used a recently published phenotyping approach for partitioning 3D facial shape, such that facial shape was divided into a nested series of global-to-local facial segments, for each cohort independently."

6. “In total, 382 and 50 classifiers were trained in the GLOBAL and EURO cohort, respectively.” – where is 50 from, shouldn’t this be 34 – the 32 snps and the 2 significant GB PC’s? or if you include sex, age and bmi to this it would be 37 (if you don’t include GB PC 2 and 3) so I think this is a typo?

7. really liked this sentence – “Doing so should more clearly expose variability (and thus system error) in the matches achieved, and, thus inform investigators regarding the performance of the algorithm on a case by case basis.

8. “….our problem is amplified in that identity has to be established from a list of hundreds of somewhat similar….”

9. Does POPRES have higher resolution European ancestry info, I didn’t think so…might be better to ref a different euro resource – perhaps the genographic project, or a merge of the two perhaps.

10. sentence needs finishing on page 16, “The performance was evaluated using cumulative match characteristic (CMC) curves which….?

Reviewer #2 (Remarks to the Author):

I'd like to thank the authors for their revised manuscript, which tries to better highlight the contributions.

However, while the authors now explicitly claim multiple differences between their approach and related work, including the very similar Lippert et al. paper, the authors overstate the novelty of their approach. Obviously, both mathods for matching images and genomes are quite complex and thus differ in many technical details, but the overall approach to matching of genomes and facial images are conceptually very similar and thus not significant, as will be detailed further below.

The main novelty claimed by the current manuscript are contributions by individual SNPs over genomic PCs that the authors claim to find in theit Europe cohort. Lippert et al., whose experimental setup is comparable to the one on the GLOBAL cohort, have thouroughly evaluated the effect of individual SNPs, finding them not predicitve on top of the use of a complete set of PCs on their particular cohort. This is in line with the current manuscripts findings on the GLOBAL cohort.

As written in my initial review, the empirical evaluation of individual SNPs on the Europe cohort is not entirely convincing. It would be more convincing if the authors evaluated the effect of SNPs on top of more than 4 PCs. The current evaluation using only 4 PCs may be anti-conservative. I would have wished that the authors would have provided additional evidence in a more conservative experiment in their revision, for example by using more than only 4 genomic PCs.

In sum, most of the claimed differences are not significant. The difference that promises to be very significant and worth being published is not evaluated sufficiently, despite recommendation to do so in my original review.

Details regarding other claimed differences in their approach:

Matching vs. face prediction - direct comparison instead of first prediction of a face and then comparing.

While the Lippert et al. paper does perform face prediction, this is not an integral part of the matching algorithm, which is based on mapping both facial images and genomes into a common continuous space and measuring distances in this space (See Materials and Methods in Lippert et al.). In particular, displaying predicted faces is not a neccessity of the Lippert et al. approach. Yet, displaing images may be considered a useful way to assess the quality of the algorithm.

In the rebuttal the authors highlight that they compare a DNA sample directly to a set of faces, without prediction of faces.

Note, that in order to compare these, the DNA and the faces need to be mapped into a common space. Whether this is by predicting DNA markers from a face (as done in Shriver et al.), a facial image embedding from DNA (as done in Lippert et al. amongst others), or a third variable (e.g., ancestry, age, sex etc. as in Lippert et al.) from both is not quite relevant.

The paper at hand is fusing multiple weak models to improve joint matching

This is done using a different, yet similar algorithm (i.e., Maximum Entropy prediction or Yasmet) to fuse multiple matching models in Lippert et al.

Using a classification algorithm instead of regression methods for prediction

While it is a difference between the approaches used, it is unclear, what the upside of a classification algorithm is, over a regression method. To demonstrate significance of classification vs. regression, the authors should further elaborate in their paper and provide empirical evidence that classification is an improvement. Intuitively, I only see the downside of a classification method that highly

informative continuous variables, such as genomic PCs, have to be discretized and thus will lose discriminative information by having to bin together multiple individuals.

linear vs. non-linear prediction models

The regression models in Lippert et al. are linear. However, most of the work in Lippert et al. is in deriving the embedding spaces. This involves quite complex pipelines and multiple very non-linear steps, making the approach as a whole quite non-linear. The current paper does not provide empirical evidence that their non-linear method is an improvement over a linear approach to show significance of this difference. Moreover, Lippert et al. have compared their linear regression methods against non-linear regression methods and have not found much difference in performance (See Supplement p.10 in Lippert et al.).

matching based on face only, not additional variables.

It is true that Lippert et al. have added additional variables and phenotypes to their analysis. However, they also have evaluated use of facial images alone (see "All Face" in Table 2 in Lippert et al). Thus this is not a novelty of this manuscript.

Reviewer #3 (Remarks to the Author):

1. It might be advisable to substitute excels with a more accurate verb. It’s not clear that machine learning excels (to be exceptionally good at or proficient in an activity or subject) in this area. While the articles cited report important successes, some of the most publicly prominent cases are the failures. See: Turner Lee, N. (2018). Detecting racial bias in algorithms and machine learning. Journal of Information, Communication and Ethics in Society, 16(3), 252-260; Broad, Ellen. "Who gets held accountable when a facial recognition algorithm fails?." IQ: The RIM Quarterly 34.4 (2018): 18.

258 Computationally, in contrast to the challenging task of genome-based phenotype prediction, this

259 new paradigm is embedded in facial image classification, an area in which machine learning

260 currently excels (i.e. 3D face based prediction of sex15, age16, ancestry17, and sexual

2. Could the authors please explain this passage in their response?

377 However, the minute differences among the group of listed faces represents the variability of the system that is exposed. Therefore, it is only correct to pin down the common aspects in the group of listed faces.

3. Could authors indicate where in the Discussion they addressed my comments (see below), which concerned the difference between what seems possible and likely in a laboratory setting and what seems possible and likely in the real world. It was not a question about what humans are technically capable of but about how these systems are likely to be used in the real world and what constraints will be introduced that are not apparent during development. (I am not asking authors to explain literally how such systems might be used or what novel constraints will be introduced in the move to real world use so much as to note that the two settings differ and that knowledge gained in the laboratory setting will go only so far predicting real worlds issues.)

My comment:

Maybe “generating and presenting multiple images” will communicate “variability in accuracy” which “might help avoid prematurely targeting a single individual… .” Arguably, however, absent research, likely by psychologists, it remains to be seen how potential users will manage hundreds of candidate images that differ from one another only in minute details.

4. Could the authors please indicate where the revised manuscript addresses my comments about machine learning? I see the phrase “resource poor” in the revised manuscript but the passage in authors’ “REVISION NOTES” (copied below) seems to contradict rather than recognize my point, which is that we know very little about what happens when complex systems based on (unsupervised) machine learning move into routine use. The development of AI might imply “a strong understanding of the infrastructure requirements, access to adequate training data and additional needs, including IT and platforms” but its use does not. By the same token, the development of “ machine learning applications require high-profile experts able to produce and work on high-quality datasets used to train machine learning algorithms” but their use does not. Arguably that’s the point—to provide expertise that is locally (or practically) unavailable. The reference to AI applications in healthcare does not eliminate this concern because it does not address the situation (which barely exists, but which is much anticipated) of unsupervised machine learning taking over large domains of healthcare decision-making.

5. I am not sure that the authors understood my comments concerning S2 (now S3). I requested and still would like to have the " argument and purpose of "Text S2 [S3] : Genomic research challenges" clarified.

Presumably the section "Genomic research challenges" addresses a subset of challenges in genomic research, not all of them. And that subset seems to be those related to identifiability, privacy, informed consent and to ways of opening up access to genomic information. While of course these topics are related to the authors' manuscript, whether this is the "appropriate scholarly literature for the method we propose," as the authors' response suggests, is unclear. In part this depends on the function of the section. Is it to point out that practices, beliefs, regulations about genomic information are in flux? Is it to suggest that public or scholarly sentiment seems to be moving in the direction where the proposed technology would be welcome? Or is it something else?

Could the authors please indicate where they have "provided references in support of both" public sentiment and scholarly sentiment. The quote about shifting public sentiment is taken from an opinion piece written by senior NHGRI administrators, and the citation relied on by that article to say public sentiment is shifting is the Patients Like Me website. Many of the other articles cited in this section are brief commentary or ethical analyses, focused largely on medical uses of genetics.

While the authors demur from speculating about how "this proof of concept approach would be evaluated" (not something I requested), they might consider whether by citing literature that "advocates for open consent" [1073], poses the possibility of achieving a "veracity of consent through candid, honest disclosure of the risks of participation [in genomic research] [1074] or that that advocates " a shift in attention from balancing data privacy and utility to enabling trust or promoting solidarity" [1076; 1077] they have not in essence at least suggested the basis for one version of a favorable reception.

Minor points:

1. Substitute raise for rise.

369 Another point that may rise concern here,

2. Individuate needs a direct object, such as faces, people, individuals.

a. 265 Any feature that gives insufficient information to individuate….

3. Consider substituting DNA-based investigations for DNA investigations.

a. This approach represents an additional and 257 complementary venue that can be used as further support in DNA investigations.

Reviewer #4 (Remarks to the Author):

The authors have provided a detailed reply to most of the critical comments. What remains is the, for the time being, too large gap between this methodology in a controlled ideal (in terms of DNA availability) environment and the current forensics practice (where there is a chronic shortage in the amount of DNA available).

This renders the method proposed here very interesting BUT NOT for a forensics application. This promise should be carefully phrased where relevant throughout this manuscript.

Otherwise, this reviewer has no further comments.

REVISION NOTES

We thank the reviewers for their constructive comments and efforts to help improve our manuscript. Below is a point-by-point response in blue to each of the reviewers, with the original reviewers' comments in italic. Also, we provided the original manuscript with changes highlighted. Some valuable discussions and experimental results are reported here, but are outside the scope of the manuscript. Therefore, we will go for the option, provided by Nature Communications, to have the review on our work published alongside the manuscript.

Reviewer #1 (Remarks to the Author):

I would like to thank the authors for taking into consideration my recommendations, and for providing constructive comments and amendments in their reply and the edited article submission. I believe the re-structuring of the paper is very well done, and the flow of text for a new and highly interesting method is excellent, well done. Below are some minor comments to fix/clarify but overall this manuscript is nearly ready for publication in this reviewer's eyes and I think the community will benefit immensely from its publication.

We appreciate the reviewer's positive evaluation of our submitted manuscript and we thank him/her for acknowledging the impact of our proof of concept into the scientific community.

Minor edits:
1. Biological runs off the tongue a little nicer than biologic for the entire text, but this is up to the author to change.

We have changed 'biologic' into 'biological' throughout the text.

2. I would edit the line to "DNA based prediction of phenotypes (hair color, eye color), and ancestry can be used to narrow down the pool of candidates onto which to perform further investigations." Ancestry is seen as independent to phenotyping classifications (not collective) in this context as it is not an externally visible trait per say.

The abovementioned sentence has been changed as suggested.

3. I don't think the line 'for the first time and in contrast to DNA phenotyping,' is needed, there is no prediction model for facial morphology yet in FDP so why say in contrast to this. Yes you have shown each snps affect in your method which is novel but you are not exactly predicting specifically an individuals or subgroup as you indicated earlier in the text that phenotyping does. Best to change it to, 'for the first time by individual genetic loci …'

We agree with this comment and have changed this sentence.

4. change to "We discuss how this work contributes to the development of new applications…"
In co-consideration, of the comment from reviewer 4, to downscale forensic implications, we have changed this sentence into: "We discuss how this work provides the user with powerful tools to establish human facial identity from DNA while avoiding some pitfalls of directly making DNA-to-face predictions."

5. change to "We used a recently published phenotyping approach for partitioning 3D facial shape, such that facial shape was divided into a nested series of global-to-local facial segments, for each cohort independently."

This sentence has been modified according to this comment.

6. "In total, 382 and 50 classifiers were trained in the GLOBAL and EURO cohort, respectively." – where is 50 from, shouldn't this be 34 – the 32 snps and the 2 significant GB PC's? or if you include sex, age and bmi to this it would be 37 (if you don't include GB PC 2 and 3) so I think this is a typo?

This was not a typo, since we have implemented 32 classifiers for SNPs under the dominant model of inheritance, 13 classifiers for SNPs under the recessive model of inheritance, and another five classifiers for sex, age, BMI, and for the two genomic PCs, for an overall number of 50 classifiers for the EURO cohort.

7. really liked this sentence – "Doing so should more clearly expose variability (and thus system error) in the matches achieved, and, thus inform investigators regarding the performance of the algorithm on a case by case basis.

We thank the reviewer for this positive comment.

8. "….our problem is amplified in that identity has to be established from a list of hundreds of somewhat similar…."

We have added 'of' before 'somewhat similar'.

9. Does POPRES have higher resolution European ancestry info, I didn't think so…might be better to ref a different euro resource – perhaps the genographic project, or a merge of the two perhaps.

We have added the reference to the Genographic Project[1] in the main text.

10. sentence needs finishing on page 16, "The performance was evaluated using cumulative match characteristic (CMC) curves which….?

This block now states: The performance was evaluated using cumulative match characteristic (CMC) curves which plot the cumulative identification rate as a function of rank, which is simply the position of the true candidate in the sorted gallery list. Identification performance is typically summarized with rank x% identification rate, reflecting the percentage of recognition results that are within the top x% of the sorted gallery. High identification rates and rapid relative increases in the CMC indicate better performance.

The next explanation on verification setup has also been expanded: "[…] For a range of thresholds on the overall matching score, the true positive fraction (TPF) is plotted against the false positive fraction (FPF). Performance measures that are typically reported are the area under the curve (AUC) and the equal error rate (EER), which is the point on the ROC where the fractions of false accept and reject are equal. Lower EER and higher AUC scores indicate better performance."

Reviewer #2 (Remarks to the Author):

The comments raised by the reviewer involve two main more detailed investigations. The first involves the influence of individual SNPs when using more than the first 4 genomic PCs in the EURO cohort. The second involves a better comparison of the algorithmic steps involved in this work compared to the work of Lippert et al.[2] We respectfully agree with the importance of these investigations and therefore we have performed a series of additional analyses, including the implementation and investigation of a lasso regression based facial prediction following the work of Lippert et al., the results of which are also incorporated into the revised manuscript. Additionally, we added a paragraph to the discussion in comparison with the work of Lippert et al. We invite the reviewer to first read the additional supplement provided in the manuscript, followed by our point by point responses to the comments raised below.

I'd like to thank the authors for their revised manuscript, which tries to better highlight the contributions.

We thank the reviewer for appreciating our efforts in improving the revised manuscript.

However, while the authors now explicitly claim multiple differences between their approach and related work, including the very similar Lippert et al. paper, the authors overstate the novelty of their approach. Obviously, both methods for matching images and genomes are quite complex and thus differ in many technical details, but the overall approach to matching of genomes and facial images are conceptually very similar and thus not significant, as will be detailed further below.

This comment is certainly justified, in the previous version of the manuscript, the claimed differences were stated mainly in the revision notes. We agree that without further data analysis these differences cannot be claimed. Therefore, in this update of the manuscript, we have implemented a facial prediction from DNA regression followed by a face-to-face matching as reported in the work of Lippert et al. In essence, Lippert et al. implemented and compared a series of regression models including ridge regression, lasso regression, ridge regression with stability selection, extreme boosted trees, support vector regression, feed-forward neural networks, and k-nearest neighbor regression, and concluded that the choice of regression model had little impact on their results. Therefore, in this update we opted for a lasso regression, since its implementation in Matlab is more advanced than the ridge regression, allowing, for example, an easy parameter tuning through cross-validation during function call.

The main novelty claimed by the current manuscript are contributions by individual SNPs over genomic PCs that the authors claim to find in their Europe cohort. Lippert et al., whose experimental setup is comparable to the one on the GLOBAL cohort, have thoroughly evaluated the effect of individual SNPs, finding them not predictive on top of the use of a complete set of PCs on their particular cohort. This is in line with the current manuscripts findings on the GLOBAL cohort. As written in my initial review, the empirical evaluation of individual SNPs on the Europe cohort is not entirely convincing. It would be more convincing if the authors evaluated the effect of SNPs on top of more than 4 PCs. The current evaluation using only 4 PCs may be anti-conservative. I would have wished that the authors would have provided additional evidence in a more conservative experiment in their revision, for example by using more than only 4 genomic PCs.

It is true that the main novelty of this manuscript is about the successful incorporation of individuals SNPs in the EURO cohort. We do understand the concern of the anti-conservative nature

of using only the first 4 PCs in the EURO cohort, which were selected following the standard paradigms of a GWAS in a single homogenous population. Therefore, we have performed a series of additional investigations as requested.

In a first instance, we tested how the statistical evidence of the 32 Peak SNPs against facial shape changes when adding more genomic PCs as conditioning variables (See Table below). In general, it is noted that the statistical evidence for association per SNP does not change substantially. When using 100 genomic PCs, 87% of the SNPs stay well below the FDRd threshold (7.7 x 10-8) and all 32 SNPs stay below the threshold of $5x10^{-5}$ that was deployed in the EURO cohort, as criterion to build a face-to-DNA classifier.

SNP	PC1-4	PC1-20	PC1-100
'rs2977562:128106267:A:G'	1.67E-15	2.40E-15	1.83E-14
'rs2821107:197343950:T:A'	2.90E-22	9.66E-22	2.71E-21
'rs10238953'	1.62E-31	2.33E-30	3.47E-28
'rs227832'	8.19E-14	3.28E-13	3.45E-13
'rs4916071:61020499:G:A'	2.26E-16	2.10E-15	6.98E-15
'rs61808932:119643820:T:C'	5.36E-22	3.42E-23	2.59E-21
'rs72866756:69128981:G:A'	2.63E-14	2.54E-14	7.33E-14
'rs200100774:119564215:G:A'	1.54E-10	7.22E-10	2.47E-11
'rs9395084:45220175:T:C'	5.19E-09	5.81E-09	2.10E-08
'rs73735344:45256286:A:G'	2.85E-12	1.74E-12	1.11E-11
'rs7966105:85577001:G:A'	7.48E-09	1.45E-08	2.65E-08
'rs949977:197343295:C:G'	2.36E-09	5.07E-09	1.25E-09
'rs11871949:70029448:C:T'	4.63E-13	2.85E-13	5.68E-12
'rs2272224:96308943:T:C'	1.16E-11	5.12E-12	2.54E-12
'rs1059045:174462975:T:C'	6.34E-13	1.97E-12	1.72E-10
'rs2424392:21628942:T:C'	7.15E-09	7.32E-09	3.13E-08
'rs402020:133609328:T:C'	5.05E-12	1.25E-11	5.36E-11
'rs1370926'	1.41E-11	1.12E-11	4.86E-10
'rs10020603:154820806:C:T'	4.50E-18	1.27E-17	2.09E-16
'rs2404983:57048961:G:A'	2.96E-08	6.67E-08	3.93E-08
'rs17299889:154831619:G:A'	4.87E-09	3.50E-09	3.64E-09
'rs970797'	4.48E-11	4.07E-11	3.65E-10
'rs2955084:127961305:T:A'	3.52E-10	1.61E-09	5.42E-10
'rs1178103'	1.02E-08	3.73E-08	1.19E-07
'rs150863859:47385400:C:G'	4.53E-08	3.94E-08	4.54E-08
'rs7930466:103900016:A:G'	5.48E-08	3.52E-08	1.48E-07
'rs7925936:113875575:T:C'	9.56E-09	1.27E-08	6.16E-09
'rs6035946:21758674:T:A'	1.49E-08	4.84E-08	4.95E-08
'rs2980419:8114141:A:T'	4.95E-08	7.27E-05	1.94E-05
'rs13290470'	2.89E-08	1.17E-07	3.37E-06
'rs2985662:22449588:A:C'	5.40E-08	1.52E-07	2.37E-07
'rs143974562:30426467:C:T'	3.18E-08	1.22E-08	1.17E-08

In a second instance, we investigated the contribution of additional genomic PCs in the EURO cohort, each time using the genomic PCs only and using the genomic PCs augmented with the 32 SNPs to

see if the contribution of the SNPs is lost when adding more genomic PC based face-to-DNA classifiers. Additional genomic PCs were selected by 1) looking beyond the first 4 genomic PCs, and 2) lowering the selection threshold from $5x10^{-5}$ to $5x10^{-4}$, $5x10^{-3}$, and $5x10^{-2}$. The biometric performances are listed in the Table below.

Genomic PCs	Threshold	Nr	EER	σ	AUC	σ	R1 (%)	σ	R10 (%)	σ	R20 (%)	σ
1 to 4	5,E-05	2	0.427	0.003	0.607	0.005	1.354	0.003	17.046	2.526	31.153	1.407
1 to 1000	5,E-05	5	0.427	0.008	0.615	0.005	1.580	0.706	17.157	1.582	32.844	0.622
1 to 1000	5,E-04	5	0.427	0.009	0.615	0.005	1.580	0.706	17.271	1.721	32.731	0.471
1 to 1000	5,E-03	204	0.445	0.018	0.577	0.014	2.032	0.590	14.785	1.188	27.537	2.878
1 to 1000	5,E-02	204	0.446	0.028	0.577	0.014	2.032	0.590	14.446	2.400	27.537	2.333
Genomic PCs + SNPs												
1 to 4	5,E-05	2	0.375	0.010	0.671	0.008	2.709	0.898	25.623	3.350	40.972	1.802
1 to 1000	5,E-05	5	0.373	0.015	0.673	0.008	2.935	0.707	25.848	2.017	41.875	1.097
1 to 1000	5,E-04	5	0.374	0.014	0.673	0.008	2.935	0.707	25.848	2.017	41.762	1.257
1 to 1000	5,E-03	204	0.395	0.004	0.645	0.008	3.386	0.344	22.686	0.301	36.457	1.947
1 to 1000	5,E-02	204	0.395	0.004	0.644	0.008	3.048	0.682	22.460	1.256	36.682	1.699

Table: Average identification and verification results over the three test runs for different selections of genomic PCs with or without SNPs in the EURO cohort. Genomic PCs, the amount of genomic PCs investigated; Threshold, the threshold applied to select genomic PCs; Nr, the amount of genomic PCs selected; EER, verification equal error rate; AUC, verification area under the curve; R1, rank 1% identification rate; R10 rank 10% identification rate; R20 rank 20% identification rate; σ standard deviation. Random performance is given as EER=0.5, AUC=0.5, R1=1%, R10 = 10%, R20 = 20%. % refers to the percentage of individuals in the gallery (EURO = 275, the test datasets).

A couple of observations are made from these results. First, at the threshold of $5x10^{-5}$ (as applied in the manuscript) the results are close to the same when investigating the first four genomic PCs only. Second, the incorporation of additional genomic PCs, by lowering the selection threshold decreases the performance notably. This also confirms that sufficient statistical association of molecular features to facial shape is required in our pipeline. Finally, in each scenario of different genomic PCs, the added SNPs improve the performances positively and consistently.

In a third instance, we refer to the results generated in the supplementary analysis using DNA-to-face regression-based predictions for the EURO cohort. We observed that facial predictions using 1000 genomic PCs did not improve on facial predictions based on the first and fourth genomic PC. The same was observed for the GLOBAL cohort, where predictions using the full set of genomic PCs did not outperform predictions using selected (statistically associated to facial shape) genomic PCs.

In conclusion, our additional analyses do not indicate any added value of using the full set of genomic PCs in the EURO cohort. Furthermore, since individual genetic loci are generally identified using a GWAS paradigm in the related scientific literature, we do prefer to do the same in this work.

In sum, most of the claimed differences are not significant. The difference that promises to be very significant and worth being published is not evaluated sufficiently, despite recommendation to do so in my original review.

This revision provides the necessary details to compare the two works and to emphasize more clearly the novelty of our method.

# Details regarding other claimed differences in their approach:

## Matching vs. face prediction - direct comparison instead of first prediction of a face and then

comparing.
While the Lippert et al. paper does perform face prediction, this is not an integral part of the matching algorithm, which is based on mapping both facial images and genomes into a common continuous space and measuring distances in this space (See Materials and Methods in Lippert et al.). In particular, displaying predicted faces is not a neccessity of the Lippert et al. approach. Yet, displaing images may be considered a useful way to assess the quality of the algorithm.
In the rebuttal the authors highlight that they compare a DNA sample directly to a set of faces, without prediction of faces.
Note, that in order to compare these, the DNA and the faces need to be mapped into a common space. Whether this is by predicting DNA markers from a face (as done in Shriver et al.), a facial image embedding from DNA (as done in Lippert et al. amongst others), or a third variable (e.g., ancestry, age, sex etc. as in Lippert et al.) from both is not quite relevant.

This is an interesting point, in the work of Lippert et al. the embedding (common space) is mainly at the level of (multiple) phenotypes. In contrast the embedding in this work is at the level of molecular features and genotypes, from a single phenotype. Once created, distances in both embeddings are defined to perform identification. Through the implementation of a regression based facial prediction from DNA, along the work of Lippert et al., we were now able to compare the results of both types of embeddings more objectively, on the same datasets and using the same biometric evaluations. From our results reported, we can see that 1) the prediction of facial shape PCs from molecular features performs less compared to the classification into molecular features from facial shape, and 2) that using face-to-DNA classifiers allows for the incorporation of individual genetic loci to further enhance the identification of an individual within a single population.

## The paper at hand is fusing multiple weak models to improve joint matching This is done using a different, yet similar algorithm (i.e., Maximum Entropy prediction or Yasmet) to fuse multiple matching models in Lippert et al.

This is correct, the main difference remains the level at which the fusing is performed, either between phenotypic matches after DNA-to-face regression or genotypic matches after face-to-DNA classification.

## Using a classification algorithm instead of regression methods for prediction
While it is a difference between the approaches used, it is unclear, what the upside of a classification algorithm is, over a regression method. To demonstrate significance of classification vs. regression, the authors should further elaborate in their paper and provide empirical evidence that classification is an improvement. Intuitively, I only see the downside of a classification method that highly informative continuous variables, such as genomic PCs, have to be discretized and thus will lose discriminative information by having to bin together multiple individuals.

We agree that more empirical evidence was required to illustrate the difference of regression in comparison to classification. Therefore, we did perform regression based facial predictions in this revision, for which any continuous variable was not converted to binary variables (as we do for the classifiers). Overall, our results show that regression only performs less than our classifier results. Furthermore, using classifiers, in contrast to regression, we were able to show the contribution of individual genetic loci.

## linear vs. non-linear prediction models
The regression models in Lippert et al. are linear. However, most of the work in Lippert et al. is in

deriving the embedding spaces. This involves quite complex pipelines and multiple very non-linear steps, making the approach as a whole quite non-linear. The current paper does not provide empirical evidence that their non-linear method is an improvement over a linear approach to show significance of this difference. Moreover, Lippert et al. have compared their linear regression methods against non-linear regression methods and have not found much difference in performance (See Supplement p.10 in Lippert et al.).

As pointed out by the reviewer, the regression model (with a focus on facial shape) in the work of Lippert et al. is linear. The more complex step in the work of Lippert et al. involves metric learning to define a weighted distance (formula 2 on page 28 of the supplement in Lippert et al.) between facial shape PCs predicted and facial shape PCs observed in a gallery of faces with known identity. The weights for the distance are obtained using maximum entropy prediction or Yasmet, which indeed involves a complex possibly non-linear optimization. However, once the weights are optimized, the distance in formula 2 on page 28 of the supplement in Lippert et al. is a weighted linear combination of differences in shape PCs from predicted to observed faces. In our supplementary analysis, we tested several alternatives to Yasmet (by lack of a Matlab implementation) to obtain the weights in formula 2. We did so, such that supervised genuine from imposter face-to-face matches were optimally separated (which is similar to using the supervised lineup optimization as in Lippert et al.). LDA based weight optimization performed the best on our data. However, in our supplementary analysis we have noted that when using facial shape PCs only, the (non-optimized) cosine distance on shape PCs represented as vectors performs better than the optimized weighted distance. Unfortunately, a similar test setup using shape PCs only, was not reported in the work of Lippert et al. Instead in the work of Lippert et al., shape PCs were augmented with color PCs and estimations of sex, age, and ancestry from faces. This restricts a further comparison of our observations.

matching based on face only, not additional variables.
It is true that Lippert et al. have added additional variables and phenotypes to their analysis. However, they also have evaluated use of facial images alone (see "All Face" in Table 2 in Lippert et al). Thus this is not a novelty of this manuscript.

We agree, and in light of the previous comments in methodological overlap we have incorporated results following a DNA-to-face regression strategy and a new paragraph in the discussion that provides a better contextualization of our work against that of Lippert et al.

Reviewer #3 (Remarks to the Author):

1. It might be advisable to substitute excels with a more accurate verb. It's not clear that machine learning excels (to be exceptionally good at or proficient in an activity or subject) in this area. While the articles cited report important successes, some of the most publicly prominent cases are the failures. See: Turner Lee, N. (2018). Detecting racial bias in algorithms and machine learning. Journal of Information, Communication and Ethics in Society, 16(3), 252-260; Broad, Ellen. "Who gets held accountable when a facial recognition algorithm fails?." IQ: The RIM Quarterly 34.4 (2018): 18.
258 Computationally, in contrast to the challenging task of genome-based phenotype prediction, this
259 new paradigm is embedded in facial image classification, an area in which machine learning

260 currently excels (i.e. 3D face based prediction of sex15, age16, ancestry17, and sexual

The verb "excels" has been substituted with the sentence "[...] which is an active area of research in machine learning [...]".

2. Could the authors please explain this passage in their response?
377 However, the minute differences among the group of listed faces represents the variability of the system that is exposed. Therefore, it is only correct to pin down the common aspects in the group of listed faces.

The proposed proof of concept allows the user to narrow down the pool of facial images by concentrating on similar facial traits among the listed individuals. The top listed individuals, are individuals that all match equally (to an extent) with the given probe-DNA. Therefore the differences between the listed individuals, are differences that were not resolved to match better or worse given the current system's ability. The common aspects on the other hand, are aspects or traits for which all listed individuals should match to the same extent with the given probe-DNA. Non-listed individuals, did not match on these common aspects. We hope this helps to understand the passage referred to by the reviewer.

3. Could authors indicate where in the Discussion they addressed my comments (see below), which concerned the difference between what seems possible and likely in a laboratory setting and what seems possible and likely in the real world. It was not a question about what humans are technically capable of but about how these systems are likely to be used in the real world and what constraints will be introduced that are not apparent during development. (I am not asking authors to explain literally how such systems might be used or what novel constraints will be introduced in the move to real world use so much as to note that the two settings differ and that knowledge gained in the laboratory setting will go only so far predicting real worlds issues.)
My comment:
Maybe "generating and presenting multiple images" will communicate "variability in accuracy" which "might help avoid prematurely targeting a single individual… ." Arguably, however, absent research, likely by psychologists, it remains to be seen how potential users will manage hundreds of candidate images that differ from one another only in minute details.
We thank the Reviewer for clarifying this point. We have now noted this issue and have added the following sentence to the manuscript: "Arguably, however, absent research, likely by psychologists, it remains to be seen how the use of such a system outside the current laboratory setting can be potentially transferred into the real world."

4. Could the authors please indicate where the revised manuscript addresses my comments about machine learning? I see the phrase "resource poor" in the revised manuscript but the passage in authors' "REVISION NOTES" (copied below) seems to contradict rather than recognize my point, which is that we know very little about what happens when complex systems based on (unsupervised) machine learning move into routine use. The development of AI might imply "a strong understanding of the infrastructure requirements, access to adequate training data and additional needs, including IT and platforms" but its use does not. By the same token, the development of "machine learning applications require high-profile experts able to produce and work on high-quality datasets used to train machine learning algorithms" but their use does not.

Arguably that's the point—to provide expertise that is locally (or practically) unavailable. The reference to AI applications in healthcare does not eliminate this concern because it does not address the situation (which barely exists, but which is much anticipated) of unsupervised machine learning taking over large domains of healthcare decision-making.

We thank again the Reviewer for clarifying his/her meaning; the following sentence was included: "Despite the growing interest and developments in AI by experts on high-quality datasets, the use and understanding of AI in practice remains daunting and challenging."

5. I am not sure that the authors understood my comments concerning S2 (now S3). I requested and still would like to have the " argument and purpose of "Text S2 [S3] : Genomic research challenges" clarified.

Presumably the section "Genomic research challenges" addresses a subset of challenges in genomic research, not all of them. And that subset seems to be those related to identifiability, privacy, informed consent and to ways of opening up access to genomic information. While of course these topics are related to the authors' manuscript, whether this is the "appropriate scholarly literature for the method we propose," as the authors' response suggests, is unclear. In part this depends on the function of the section. Is it to point out that practices, beliefs, regulations about genomic information are in flux? Is it to suggest that public or scholarly sentiment seems to be moving in the direction where the proposed technology would be welcome? Or is it something else?

Could the authors please indicate where they have "provided references in support of both" public sentiment and scholarly sentiment. The quote about shifting public sentiment is taken from an opinion piece written by senior NHGRI administrators, and the citation relied on by that article to say public sentiment is shifting is the Patients Like Me website. Many of the other articles cited in this section are brief commentary or ethical analyses, focused largely on medical uses of genetics.

While the authors demur from speculating about how "this proof of concept approach would be evaluated" (not something I requested), they might consider whether by citing literature that "advocates for open consent" [1073], poses the possibility of achieving a "veracity of consent through candid, honest disclosure of the risks of participation [in genomic research] [1074] or that that advocates " a shift in attention from balancing data privacy and utility to enabling trust or promoting solidarity" [1076; 1077] they have not in essence at least suggested the basis for one version of a favorable reception.

We agree that the section about genomic research challenges is indeed focused on privacy and identifiability, as these are specific challenges related to our work. Therefore we have changed the heading to "Re-identification and privacy challenges in genomic research", to make this more explicit.

We thank the reviewer for giving significant attention to the ethical, legal, and social issues that are related to the proof of concept method we propose here. We thoroughly agree that these issues are of tremendous importance and that attention (including significant time, effort, and public research funding resources) should be dedicated to assessing the magnitude of these issues and generating, implementing, and studying the efficacy of potential solutions. However, we strongly believe that adequately addressing these vast and complex issues, is not possible within the scope of the current manuscript. The supplemental text and references cited therein were never

offered as a comprehensive literature review or presented in a way to suggest that we covered all possible topics or provided citation to all relevant references. We selected pieces to provide a starting point for readers who want to contextualize this proof of concept approach.

The reviewer's comments underscore why there are such a diverse set of ELSI research domains prioritized by the National Human Genome Research Institute today (See https://www.genome.gov/27543732/elsi-research-domains/), including re-identification, security, and data privacy topics such as "potential identifiability of genomic information and approaches for minimizing re-identification" and "public tolerance for genomic privacy risks." The critical importance of rigorous ELSI research is increasingly recognized beyond genomics to include other data sources and technologies. The EU-funded SIENNA Project, for example, focuses on genomics, AI/robotics/human-machine-interaction, and human enhancement (see, e.g., http://www.sienna-project.eu) and involves partnerships with members of the public, experts (including researchers from 10 universities), and policy stakeholders in multiple countries.

It is unclear whether the Reviewer is suggesting that because we are not trying to do all things or be exhaustive in this supplemental section we should remove it entirely or whether there are specific lines of scholarship and public sentiment to which the Reviewer is personally aware and thinks we have inexcusably omitted. We supplied a number of references that relate to the general notion that privacy interests and preferences are not static and are context-specific throughout that section. The reference to Rodriguez et al.[3] does internally cite Patients Like Me, as the reviewer noted; however, Patients Like Me is not merely a website, and this reference does connect expert and public sentiments. Patients Like Me is a network of >600,000 people who have come together to share data for the possibility of advancing scientific discovery despite the personal risks and the size of it has been growing. We also had cited to Majumder et al.'s work[4] on public resistance to global data sharing. A number of other options could be cited (empirical and anecdotal; public or academic). For example, we could have mentioned the emergence of the PEER Platform by Genetic Alliance, the growing number of people aggregating individual-level data at Open Humans, etc. We could have pointed readers to public sentiments on privacy generally (not specific to genomics) that have always been shifting and continue to do so, as evinced by PEW survey findings (e.g.,http://www.pewresearch.org/fact-tank/2016/09/21/the-state-of-privacy-in-america/) and summarized by privacy advocates like EPIC (See, e.g., https://epic.org/privacy/survey/). Tech giants including Tim Cook at Apple have begun to advocate for the US privacy regulatory oversight to be changed dramatically, signaling a general shift in sentiment regarding the sector-by-sector approach to privacy protections and a desire to harmonize with a blanket approach as envisioned by the GDPR in Europe (https://arstechnica.com/tech-policy/2018/10/tim-cook-calls-for-strong-us-privacy-law-rips-data-industrial-complex/). We think a supplemental section in this manuscript is not an effective venue for a law review outlining the legal, regulatory, and practical reasons why such a universal approach in the United States is not readily feasible or outlining the diversity of opinions as to the utility or efficacy of a blanket approach to privacy such as that of the GDPR (e.g., Cornell professor Helen Nissenbaum has voiced strong criticism of a consent-approach to data privacy, and Laura Noren of the NYU Center for Data Science has similarly criticized GDPR's approach as an ineffective "delete and retreat" model). The majority of us are not ELSI researchers or experts. Accordingly, we believe it would be inappropriate for this manuscript to delve too much into that space: such a nuanced ELSI discussion is better handled as the subject of a law review article. We have added a reference specifically to the recently published and, in our opinion, very well-done systematic literature review on individuals' perspectives of genomic privacy authored by Clayton et al. (2018)[5].

Again, in the main text we had explained that this section is offered to underscore the importance of continued deliberation and additional ELSI in this area. We reiterate here that our purpose is to provide a starting point by which readers can contextualize the proof of concept

approach and begin to consider some, but admittedly not all, of the implications of such a proof of concept approach. Privacy is, in the United States, not a legal right protected uniformly, and so any application (whether in or out of law enforcement or health care contexts) of the method we discuss here would need to have its own risk assessment. To make our purpose even clearer, we have added additional text to the beginning of this supplemental section. We also have added some additional citations and toned down the positive light in which we discuss open access.

Minor points:

We agree on these minor points and have adjusted the text accordingly.

1. Substitute raise for rise.
369 Another point that may rise concern here,
2. Individuate needs a direct object, such as faces, people, individuals.
a. 265 Any feature that gives insufficient information to individuate….
3. Consider substituting DNA-based investigations for DNA investigations.
a. This approach represents an additional and 257 complementary venue that can be used as further support in DNA investigations.

Reviewer #4 (Remarks to the Author):

The authors have provided a detailed reply to most of the critical comments. What remains is the, for the time being, too large gap between this methodology in a controlled ideal (in terms of DNA availability) environment and the current forensics practice (where there is a chronic shortage in the amount of DNA available).
This renders the method proposed here very interesting BUT NOT for a forensics application. This promise should be carefully phrased where relevant throughout this manuscript.
Otherwise, this reviewer has no further comments.

We thank the reviewer for acknowledging that critical issues were explained in detail in the previous rebuttal. We agree with him/her and acknowledge the gap between our proof of concept and current forensic practice. We made changes to the main text to downscale the use in forensics today given our proof of concept and the need for future efforts.

In the abstract: We clearly state that future efforts are needed before forensic applications can be tackled.

In the introduction: To avoid referencing to concrete forensic applicability of our method, we substitute "new applications in forensics" with "the user" in the following sentence: "We discuss how this work provides us with powerful tools to establish human facial identity from DNA […]."

In the discussion: We removed "In current forensic practice" at the start of the Discussion.

In the discussion: To highlight the challenges in practical forensic settings we have added "important" to "challenge in forensics" within the following sentence: "[…] Finally, a future and important challenge in forensics involves the ability to use our paradigm based on often limited and contaminated DNA material. […]".

In the discussion: In order to take out the potential suggestions that the results are forensically useful, we have substituted the word "investigators" with "the user" in the following sentence: "[…] Doing so should more clearly expose variability (and thus system error) in the matches achieved,

and, thus inform the user regarding the performance of the algorithm on a case by case basis. […] ".

In the discussion: We removed the following section: "Investigators might also be interested in the verification of a match between a particular known person and the probe DNA under investigation. Depending on the question being addressed, an appropriately stringent operating point in the ROC analysis should be chosen. A confirmative exclusion of a person of interest can be obtained when the face-to-DNA overall matching score is below a low threshold, and making sure that the number of false conclusions converges to zero at the chosen threshold."

In the final concluding paragraph we avoid referring to forensic applications and emphasize that our results are preliminary and on well-defined data cohorts.

REFERENCES

1. Behar, D. M. *et al.* The Genographic Project Public Participation Mitochondrial DNA Database. *PLoS Genet.* **3**, e104 (2007).
2. Lippert, C. *et al.* Identification of individuals by trait prediction using whole-genome sequencing data. *Proc. Natl. Acad. Sci.* **114**, 10166–10171 (2017).
3. Rodriguez, L. L., Brooks, L. D., Greenberg, J. H. & Green, E. D. The Complexities of Genomic Identifiability. *Science (80-.).* **339**, 275–276 (2013).
4. Majumder, M. A., Cook-Deegan, R. & McGuire, A. L. Beyond Our Borders? Public Resistance to Global Genomic Data Sharing. *PLOS Biol.* **14**, e2000206 (2016).
5. Clayton, E. W., Halverson, C. M., Sathe, N. A. & Malin, B. A. A systematic literature review of individuals' perspectives on privacy and genetic information in the United States. *PLoS One* **13**, e0204417 (2018).

Reviewers' comments:

Reviewer #2 (Remarks to the Author):

I thank the authors for providing substantial data to address my review comments and demonstrate that individual SNPs improve identification in their approach. In my view this is the most important contribution of the paper and therefore demonstrates significance.

Here are my detailed comments regarding the new information provided:

To address my concerns regarding the choice of number of PCs used in the EURO cohort (=4), the authors present two experiments:

First, they show that the P-values obtained for 31 lead SNPs do not change much. This experiment alone is not a convincing argument for inclusion of 4 PCs only, as it is hard to draw coclusions about the null distribution (i.e. P-value inflation in SNPs that are not associated with the trait) from a small number of variants that are not drawn i.i.d. from the null distribution, but rather are ascertained based on small P-values. A more convincing experiment would compare the genome-wide distribution of P-values, for example using the genomic control statistic (Devlin & Roeder 1999).

Second, the authors compare identification results for models that include 4 and 1000 genomic PCs, respectively, with and without the inclusion of SNPs. The results indicate that a model that includes 1000 genomic PCs still improves, when including SNPs. This seems like an important result that should be mentioned in the main text to better motivate the choice of 4 PCs and therefore should at least included in the SI. Further, the experiment suggests that a model with 4 PCs outperforms a model with 1000 PCs (independent of inclusion of SNPs).

In their supplement, the authors present a comparison against a phenotype prediction approach similar to Lippert et al. based on their phenotype embeddings. The differences between the methods are the use of lasso regression instead of ridge regression and LDA instead of Yasmet for metric learning.

From the experiments, the authors conclude a) that cosine distance outperforms metric learning in the context of phenotype regression Figures S1.1, S1.2 and Table S1.1.

Using cosine and Lasso on their embeddings, the authors then compare phenotype regression (1) on multiple phenotypes from the genome with classification (2) of genomic info from multiple phenotypes. The results indicate that (1) outperforms (2).

While the result of this comparison are valuable information, the experiment for a) indicates overfitting of the metric learning approach implemented by the authors. LDA should be able to recover cosine as a special case (by outputting equal weights for all features). Such an effect could for example happen, when the algorithm is using a large number of shape PCs compared to the number of training lineups used to optimize the metric and could likely be avoided by appropriate use of regularization techniques. Therefore, the authors should not draw conclusions regarding the metric learning approach implemented in Lippert et al. based on their experiment.

There seems to have been a misunderstanding regarding my comments on non-linearities in Lippert et al. that I would like to clarify: My comment regarding non-linearities of the pipeline did not refer to the maximum entropy approach (i.e. Yasmet), which indeed is linear, but rather to feature generation (i.e. obtaining the face PCs from raw image data). The features (i.e. face PCs) that go into a linear regression are generated using non-linear pipelines applied to the raw image data (i.e. the 3D voxel data obtained from the stereo camera). Optimizing these engineered pipelines, which involve multiple data transforms from the computer graphics literature to obtain a good set of face PCs, will heavily influence the results of the whole identification method. Therefore, it is hard to argue that the approach is really linear, even though the actual regression algorithm that is applied to these face PCs and the method for combination of similarities/distances are linear.

Reviewer #3 (Remarks to the Author):

Claes et al "Facial Recognition from DNA using face-to-DNA classifiers."

1. I appreciate the authors' explanation. I was hoping however that they would revise rather than only explain the sentence, which is awkward and confusing. Is this an accurate revision?

However, the minute differences among the group of listed faces in this group represent [no "s"]the variability of the system. that is exposed. Therefore, it is only correct to pin down important to describe what these faces share. the common aspects in the group of listed faces.

If so, could the sentence be expressed as follows:

However, because the minute differences among these faces represent the variability of the system, it's important to describe their commonalities.

If the latter version is accurate, however, I am not sure I understand how the second clause (or in the original version, the second sentence: Therefore, it is only correct to pin down the common

aspects in the group of listed faces. is a consequence of the first. If the authors choose to address this point, please do not explain the theory behind your claims. My concern is with the language.

2. 3. 4. These revisions are fine.

5. The length and content of author's response to Comment #5 is puzzling. Comment #5 did not ask authors provide an analysis of the "vast and complex" "ethical, legal and social issues" this manuscript raises. Nor does the reviewer assume that S4 was offered " as a comprehensive literature review or presented in a way to suggest that we covered all possible topics or provided citation to all relevant references." (However as noted, the function of the section was unclear, which the authors have addressed by providing a more descriptive title.)

However while authors do not need to provide a comprehensive review, they do need to provide a balanced one. The current one falls short in three ways. Please do not over interpret these remarks or assume that they raise broad conceptual issues with S4.

The account provided by S4 is imbalanced because 1) it refers to public sentiment but is based almost entirely on expert commentary; 2) it restricts its account to scholarship that implicitly or explicitly endorses expanding access to and circulation of genetic information. Because of (1) this confers on these proposals a sense of acceptance or inevitability and risks substituting what is perhaps the authors' views for broader or empirically based ones. For example, based on what do we know that "genetic exceptionalism continues to be discouraged?" By whom and in what ways?; and, 3) While the proof of concept at issue in the manuscript concerns forensic uses of genetics, S4 relies almost entirely on work concerning medical uses. The difference is unlikely to go unnoticed by the public; it should go unremarked here.

REVISION NOTES

As a general revision note, in order to comply with the formatting checklist provided by the editors, we have reduced the amount of words in the abstract to <= 150 words and the main text (introduction, results and discussion) to <=5000 words. We have done this, by simplifying sentences where possible without compromising to any additions or changes made as requested during the whole review process. Furthermore, we have corrected some colors and references to Figures and Supplementary material according to guidelines. Below we provide specific revision notes in response to the few remaining comments raised by the reviewers.

Reviewer #2 (Remarks to the Author):

I thank the authors for providing substantial data to address my review comments and demonstrate that individual SNPs improve identification in their approach. In my view this is the most important contribution of the paper and therefore demonstrates significance.

We thank the reviewer for the positive comment and the acknowledgement that the contribution of SNPs improve identification in the EURO cohort.

Here are my detailed comments regarding the new information provided:

To address my concerns regarding the choice of number of PCs used in the EURO cohort (=4), the authors present two experiments:

First, they show that the P-values obtained for 31 lead SNPs do not change much. This experiment alone is not a convincing argument for inclusion of 4 PCs only, as it is hard to draw conclusions about the null distribution (i.e. P-value inflation in SNPs that are not associated with the trait) from a small number of variants that are not drawn i.i.d. from the null distribution, but rather are ascertained based on small P-values. A more convincing experiment would compare the genome-wide distribution of P-values, for example using the genomic control statistic (Devlin & Roeder 1999).

Second, the authors compare identification results for models that include 4 and 1000 genomic PCs, respectively, with and without the inclusion of SNPs. The results indicate that a model that includes 1000 genomic PCs still improves, when including SNPs. This seems like an important result that should be mentioned in the main text to better motivate the choice of 4 PCs and therefore should at least included in the SI. Further, the experiment suggests that a model with 4 PCs outperforms a model with 1000 PCs (independent of inclusion of SNPs).

We respectfully agree with the reviewer that the first experiment alone is not conclusive. Therefore, we previously ran different analyses, such as the second experiment described here by the reviewer. We also agree that the outcomes of this particular experiment are of interest, and have incorporated it in the Supplementary Material and have mentioned it in the main text, as suggested.

The suggestion of using the genomic control statistic is valued, however, since we wanted to test if our individual SNPs were not false positives due to population stratification (i.e. words whether they added to the information of genomic PCs or not) we followed the later work of Price et al. [1]. We further consulted other colleagues from statistical genetics, and although considered an alternative, the genomic control statistic is less used in practice today and not considered to be better in adjusting for population stratification. Nevertheless, ascertaining small p-values alone is not conclusive as mentioned by the reviewer, therefore we have not included this particular experiment in the supplementary materials and instead focus more on the other (now included) experiments.

In their supplement, the authors present a comparison against a phenotype prediction approach similar to Lippert et al. based on their phenotype embeddings. The differences between the methods are the use of lasso regression instead of ridge regression and LDA instead of Yasmet for metric learning.

From the experiments, the authors conclude a) that cosine distance outperforms metric learning in the context of phenotype regression Figures S1.1, S1.2 and Table S1.1.

Using cosine and Lasso on their embeddings, the authors then compare phenotype regression (1) on multiple phenotypes from the genome with classification (2) of genomic info from multiple phenotypes. The results indicate that (1) outperforms (2).

While the result of this comparison are valuable information, the experiment for a) indicates overfitting of the metric learning approach implemented by the authors. LDA should be able to recover cosine as a special case (by outputting equal weights for all features). Such an effect could for example happen, when the algorithm is using a large number of shape PCs compared to the number of training lineups used to optimize the metric and could likely be avoided by appropriate use of regularization techniques. Therefore, the authors should not draw conclusions regarding the metric learning approach implemented in Lippert et al. based on their experiment.

This particular analysis was not intended to draw any conclusions regarding the implementation in Lippert et al. It was intended to investigate the use of a cosine distance versus (LDA based) metric learning applied to our particular datasets and biometric evaluators. Therefore, we agree with the reviewer and have removed sentences and specific wording that potentially lead to drawing conclusions regarding the implementations in Lippert et al.

With respect, we disagree that the results in a) are likely due to overfitting. The reviewer is right in stating that using a large number of PCs compared to the number of training samples can lead to overfitting. However, after further investigation, this is not the case in our setup:

1) We have 50 PCs and n=591 samples in the validation set of our cohort (that generates 591 genuine matches (true positives) and an even more imposter matches (true negatives)) to train the LDA that is subsequently applied to the non-overlapping test set (n=295). The Vapnik–Chervonenkis (VC) dimension, as a measure of model complexity, for our LDA classifier is 51 (the number of dimensions d+1) [2]*. As quoted from* [3]*, "In practice, good generalization performance is expected if the number of training samples is a few times the VC dimension. A good rule of thumb is to choose n to be of the order of 10 times the VC dimension". In our setup, the number of learning samples is larger than 10 times the VC dimension of the LDA classifier.*

2) We have tested models with a lower VC dimension (by gradually increasing the number of PCs included from 1 to 50). We did not find a model with lower complexity (fewer PCs) that outperformed the model based on 50 PCs, which otherwise would have been a clear indicator of overfitting. We did notice that the first 15 to 20 PCs only, were enough to obtain the results as reported in the supplementary material, but again, increasing the amount of PCs up to 50 did not decrease performance, which would have been a sign of overfitting.

3) In the situation of equal weights, the distance as formulated in the work of Lippert et al. becomes the Manhattan Distance or the L1 norm of the differences between two multidimensional data points. We have tried the situation of equal weights, and observed a much lower performance (EER=0.47) in a), which is close to random performance (EER=0.5). Therefore, we believe that LDA to determine the weights in the distance, is still learning "something useful" in a).

4) The VC dimension of the follow-up experiment, adding the estimated features of facial sex, age, BMI and GB is equal to 56, but here the metric learning was successful and better than the previous model with a lower complexity (VC=51). This is somewhat counterintuitive if the results in a) were due to overfitting.

However, as stated earlier, our aim is not to draw any conclusions outside our own work, and we have changed the supplementary materials related as suggested by the reviewer.

There seems to have been a misunderstanding regarding my comments on non-linearities in Lippert et al. that I would like to clarify: My comment regarding non-linearities of the pipeline did not refer to the maximum entropy approach (i.e. Yasmet), which indeed is linear, but rather to feature generation (i.e. obtaining the face PCs from raw image data). The features (i.e. face PCs) that go into a linear regression are generated using non-linear pipelines applied to the raw image data (i.e. the 3D voxel data obtained from the stereo camera). Optimizing these engineered pipelines, which involve multiple data transforms from the computer graphics literature to obtain a good set of face PCs, will heavily influence the results of the whole identification method. Therefore, it is hard to argue that the approach is really linear, even though the actual regression algorithm that is applied to these face PCs and the method for combination of similarities/distances are linear.

We thank the reviewer for this clarification. We have double checked that we avoid referring to the work of Lippert et al. as being linear in the main manuscript and supplementary materials.

Reviewer #3 (Remarks to the Author):

1. I appreciate the authors' explanation. I was hoping however that they would revise rather than only explain the sentence, which is awkward and confusing. Is this an accurate revision?
However, the minute differences among the group of listed faces in this group represent [no "s"] the variability of the system. that is exposed. Therefore, it is only correct to pin down important to describe what these faces share. the common aspects in the group of listed faces.
If so, could the sentence be expressed as follows:
However, because the minute differences among these faces represent the variability of the system, it's important to describe their commonalities.
If the latter version is accurate, however, I am not sure I understand how the second clause (or in the original version, the second sentence: Therefore, it is only correct to pin down the common aspects in the group of listed faces. is a consequence of the first. If the authors choose to address this point, please do not explain the theory behind your claims. My concern is with the language.

We acknowledge the difficulty of being precise in language on this aspect, and appreciate the input from the reviewer. We have therefore changed the sentences that caused confusion, with the sentence suggested.

2. 3. 4. These revisions are fine.

We thank the reviewer.

5. The length and content of author's response to Comment #5 is puzzling. Comment #5 did not ask authors provide an analysis of the "vast and complex" "ethical, legal and social issues" this manuscript raises. Nor does the reviewer assume that S4 was offered "as a comprehensive literature review or presented in a way to suggest that we covered all possible topics or provided citation to all relevant references." (However as noted, the function of the section was unclear,

which the authors have addressed by providing a more descriptive title.) However, while authors do not need to provide a comprehensive review, they do need to provide a balanced one. The current one falls short in three ways. Please do not over interpret these remarks or assume that they raise broad conceptual issues with S4. The account provided by S4 is imbalanced because

1) it refers to public sentiment but is based almost entirely on expert commentary;

We have removed any reference to public sentiment.

2) it restricts its account to scholarship that implicitly or explicitly endorses expanding access to and circulation of genetic information.

We have added the following sentences to avoid the impression that open-access is universally accepted: "Support for an open approach is not universal, with some warning of the negative consequences of a surveillance state and the challenges of an informed consent approach for genomic research that remains focused on an individual, which fails to account for the probabilistic information that can be gleaned—and societal risks that accompany those insights—regarding unaware relatives or community members. Again others might advocate against expanding open access to and circulation of genetic information. No consensus solution to these and other complex issues has yet arisen from ELSI-research on policymakers."

Because of (1) this confers on these proposals a sense of acceptance or inevitability and risks substituting what is perhaps the authors' views for broader or empirically based ones. For example, based on what do we know that "genetic exceptionalism continues to be discouraged?" By whom and in what ways?

We have removed the following sentence: "Yet identifiability is not a problem limited to the sphere of genomics, and genetic exceptionalism continues to be discouraged".

3) While the proof of concept at issue in the manuscript concerns forensic uses of genetics, S4 relies almost entirely on work concerning medical uses. The difference is unlikely to go unnoticed by the public; it should go unremarked here.

We have taken the following actions to address this and to make sure the difference does not go unnoticed.

1) We have removed statements that were too restrictive in referring to medical use only, or have expanded these outside the medical use.

2) We have added a paragraph on concerns related to increasing law enforcement use of DNA, facial recognition, and other emerging technologies and that is not focused on medical use. This in order provide a better balance throughout the whole section.

"Legal scholars have written extensively on some of the constitutional concerns related to increasing law enforcement use of DNA, facial recognition, and other emerging technologies (including the general use of Big Data, machine learning, and artificial intelligence) [See, e.g., Koops and Schellekens[4], Maclean[5], Wagner[6], Gabel Cino[7,8], Gusella[9], Hodge[10], Hirose[11], Pearlman and Lee[12], Nakar and Greenbaum[13], Simmons[14], Joh[15], Berman[16], Ferguson[17], Brown[18], Kohne[19], Reamay[20], Monajemi[21], Pope[22], Carrero[23], Cuador[24], Sklansky[25], Murphy[26], Kaye[27], Dedrickson[28], Ram[29,30], Guest[31], Logan[32], Strutin[33], Garrett[34], Ferguson[17,35], Froomkin[36]]. Biometric identifiers have been described as "one of the most unprotected areas of our personal identity"[22], and scholars have lamented the many ways in which the public is being "desensitized"[21] to "privacy-sacrificing technologies"[37] or "privacy piercing technology."[38]. Some[15] have underscored the importance in recognizing the public's acts of resistance to governmental surveillance in order to make sense of privacy in modern society. While some[17] argue that a "big data-infused reasonable suspicion standard" is possible, others[20] urge us to abandon a quest for a bright-line rule when setting the

boundaries for governmental searches and seizures involving specific technological tools and instead focus on core principles of the Fourth Amendment as an "expression of shared values" that can be ascertained by courts using empiricism and social science to determine what those shared values are. Yet other scholars[39], with regard to facial recognition technology, have focused on a distinction between the right to be seen in public and the right to be recognized. Particularly relevant to this proof of concept, if it were to be applied by law enforcement, is the concern that some legal scholars have voiced regarding the need for oversight because privacy concerns will actually increase as the technology's accuracy improves[8]. Scholars have been divided[26–28] about whether universal databases could be preferable and even increase privacy[28] relative to known, current approaches. One[33] has even remarked that "the registry of human blueprints will be the never-ending battleground of privacy."

3) We have explicitly mentioned that much ELSI research cited in the section is focused on medical applications and refocused more towards forensics:

"...Much ELSI research on privacy challenges in genomic research is focused on medical applications, which is also reflected by much of the citations using throughout this Supplementary Note. With regard to forensic contexts, there have been calls to protect privacy as well as enhance oversight of crime labs[34]. One particular target of concern has been the potential exemption of forensic databases from the Privacy Act of 1974 (such as the concerns legal scholars have raised regarding the FBI's Next Generation Identification System), which would make it difficult not only to know if a specific individual's data is contained therein but also to control the agencies and parties with whom the data are shared without consent[22,23]."

References

1. *Price, A. L. et al. Principal components analysis corrects for stratification in genome-wide association studies. Nat. Genet.* ***38****, 904–909 (2006).*

2. *Neto, U. M. B. & Dougherty, E. R. Error Estimation for Pattern Recognition. (John Wiley & Sons, 2015).*

3. *Theodoridis, S. & Koutroumbas, K. Pattern Recognition. (Academic Press, 2008).*

4. *Koops, B.-J. & Schellekens, M. H. M. Forensic DNA Phenotyping: Regulatory Issues. Ssrn 1–38 (2007). doi:10.2139/ssrn.975032*

5. *Maclean, C. E. Creating a Wanted Poster from a Drop of Blood: Using DNA Phenotyping to Generate an Artist' s Rendering of an Offender Based Only on DNA Shed at the Crime Scene Part of the Civil Rights and Discrimination Commons, and the Criminal Law Commons Recommended. Hamline Law Rev.* ***36****, 1–26 (2014).*

6. *Wagner, J. K. & Brennan, J. Dna , Racial Disparities , and Biases in Criminal Justice : Searching for Solutions. Albany Law J. Sci. Technol.* ***27****, 95–138 (2017).*

7. *Gabel Cino, J. Tackling Technical Debt: Managing Advances in DNA Technology that Outpace the Evolution of Law. J. Civ. Leg. Sci.* ***54****, 420–21 (2016).*

8. *Cino, J. G. Deploying the Secret Police: the Use of Algorithms in the Criminal Justice System. Ga. St. U. L. Rev.* ***34****, 1093–94 and 1101 (2018).*

9. *Gusella, D. No Cilia Left Behind: Analyzing the Privacy Rights in Routinely Shed DNA Found at Crime Scenes. L. Rev* ***54****, 789 (2013).*

10. *Hodge, S. Current Controversies in the Use of DNA in Forensic Investigations. Univ. Balt. Law Rev.* ***48****, 65–66 (2018).*

11. *Hirose, M. Privacy in public spaces: the reasonable expectation of privacy against the dragnet use of facial recognition technology. Conn. L. Rev.* ***49****, (2017).*

12. *Pearlman, A. R. & Lee, E. S. National Security, Narcissism, Voyeurism, and Kyllo: How Intelligence Programs and Social Norms Are Affecting the Fourth Amendment. Texas A&M Law Rev.* ***2****, (2014).*

13. *Greenbaum, D. & Nakar, S. Now You See Me: Now You Still Do: Facial Recognition Technology and the Growing Lack of Privacy. Bost. Univ. J. Sci. Technol. Law* ***23****, 88–122 (2017).*

14. *Simmons, R. Quantifying Criminal Procedure: How to Unlock the Potential of Big Data in Our Criminal*

Justice System. Ssrn 1–51 (2016). doi:10.2139/ssrn.2816006

15. *Joh, E. E. Privacy Protests: Surveillance Evasion and Fourth Amendment Suspicion. Ariz. L. Rev.* ***55****, 997–1029 (2013).*

16. *Berman, E. A government of laws and not machines. B.U. L. Rev.* ***98****, 1277–1355 (2018).*

17. *Ferguson, A. G. Big Data and Predictive Reasonable Suspicion. U. Pa. L. Rev.* ***163****, 327–336 (2015).*

18. *Brown, K. N. Anonymity, Faceprints, and the Constitution. Geo. Mason L. Rev.* ***21****, 409–466 (2014).*

19. *Kohne, N. & Salour, K. Biometric Privacy Litigation: Is Unique Personally Identifying Information Obtained from a Photograph Biometric Information? Compet. J. Anti., UCL Priv. Sec. St. B. Cal.* ***25****, (2016).*

20. *Reamey, G. S. Constitutional Shapeshifting: Giving the Fourth Amendment Substance in the Technology Driven World of Criminal Investigation. Stanford J. Civ. Rights Civ. Lib.* ***14****, 201–245 (2018).*

21. *Monajemi, M. Privacy Regulation in the Age of Biometrics That Deal With a New World Order of Information. U. Miami Int'l Comp. L. Rev.* ***25****, 407–08 (2018).*

22. *Pope, C. Biometric Data Collection in an Unprotected World: Exploring the Need for Federal Legislation Protecting Biometric Data. J.L. Pol'y* ***26****, 769, 770 (2018).*

23. *Carrero, A. Biometrics and federal databases: could you be in it? John Marshall Law Rev. 1–21 (2019).*

24. *Cuador, C. From Street Photography To Face Recognition: Distinguishing Between The Right To Be Seen And The Right To Be Recognized. Nova Law Rev.* ***41****, (2017).*

25. *Sklansky, D. A. Two More Ways Not to Think about Privacy and the Fourth Amendment. Univ. Chicago Law Rev.* ***82****, 223–242 (2015).*

26. *Murphy, E. Relative Doubt: Familial Searches of DNA Databases. Mich. Law Rev.* ***109****, 329–30 (2010).*

27. *Kaye, D. H. & Smith, M. E. DNA identification databases: Legality, legitimacy, and the case for population-wide coverage. Winsconsin Law Rev.* ***3****, 414–459 (2003).*

28. *Dedrickson, K. Universal DNA databases: a way to improve privacy? J. Law Biosci.* ***4****, 637–647 (2017).*

29. *Ram Natalie. Incidental Informants Police Can Use Genealogy Databases to Help Identify Criminal Relatives-but Should They? Md. B.J. 8–9 (2018).*

30. *Ram, N., Guerrini, C. J. & McGuire, A. L. Genealogy databases and the future of criminal investigation. Science* ***360****, 1078–1079 (2018).*

31. *Guest Christine. DNA and Law Enforcement: How the Use of Open Source DNA Databases Violates Privacy Rights. Am. Univ. Law Rev.* ***68****, (2019).*

32. *Logan, W. A. Policing Police Access to Criminal Justice Data. Iowa L. Rev.* ***104****, (2019).*

33. *Strutin, K. DNA Without Warrant: Decoding Privacy, Probable Cause and Personhood. Rich. J.L. Pub. Int.* ***18****, 319–366 (2015).*

34. *Garrett, B. L. The Crime Lab in the Age of the Genetic Panopticon (Book Review). (2017).*

35. *Ferguson, A. G. Personal Curtilage: Fourth Amendment Security in Public. Wm. Mary L. Rev.* ***55****, 1283–1284 (2014).*

36. *Froomkin, A. M. Regulating Mass Surveillance as Privacy Pollution: Learning from Environmental Impact Statements. U. Ill. L. Rev. 1713–1716 (2015).*

37. *Pearlman, A. & Lee, E. National Security, Narcissism, Voyeurism, and Kyllo: How Intelligence Programs and Social Norms are Affecting the Fourth Amendment. Tex. A&M L. Rev.* ***2****, 776–778 (2015).*

38. *Nesbitt Cosby, T. The Expectation of Privacy: An Unreasonable Standard in an Era of Rapid Innovations in Technology. Charlest. L. Rev.* ***12****, (2018).*

39. *Cuador, C. From Street Photography to Face Recognition: Distinguishing Between the Right to Be Seen and the Right to Be Recognized. Nov. L. Rev.* ***41****, 237–264 (2017).*
